# Targeted sequencing of *NOTCH* signaling pathway genes and association analysis of variants correlated with mandibular prognathism

**DOI:** 10.1186/s13005-021-00268-0

**Published:** 2021-05-26

**Authors:** Xianzhuo Han, Xueyan Xiong, Xiujuan Shi, Fengshan Chen, Yongming Li

**Affiliations:** 1 Department of Orthodontics, School and Hospital of Stomatology, Shanghai Engineering Research Center of Tooth Restoration and Regeneration, Tongji University, Middle Yanchang Road, 399 Shanghai, P.R. China; 2 Department of Stomatology, Shanghai East Hospital Affiliated to Tongji University, Shanghai, China; 3 Tongji University School of Medicine, Shanghai, China

**Keywords:** Targeted sequencing, *NOTCH* signaling pathway, Association analysis, Mandibular prognathism

## Abstract

**Introduction:**

The purpose of this study was to systematically identify variants in *NOTCH* signaling pathway genes that correlate with mandibular prognathism (MP) in the general Chinese population.

**Methods:**

Targeted sequencing of *NOTCH* signaling pathway genes was conducted in 199 MP individuals and 197 class I malocclusion control individuals. The associations of common and rare variants with MP, cephalometric parameters, and continuous cephalometric phenotypes were analyzed by principal component (PC) analysis. The associations between rare variants and MP were tested for each gene.

**Results:**

Six SNPs, including rs415929, rs520688, and rs423023 in an exonic region of *NOTCH4*; rs1044006 in an exonic region of *NOTCH3;* rs1051415 in an exonic region of *JAG1*; and rs75236173 in the 3′-untranslated region (3′-UTR) of *NUMB* were associated with MP (*P* < 0.05). One common variant, rs1051415, in an exonic region of *JAG1* was significantly related to PC1 (*P*  = 3.608 × 10^− 4^), which explained 24.3% of the overall phenotypic variation observed and corresponded to the sagittal mandibular position towards the maxilla, ranging from a posterior positioned mandible to an anterior positioned mandible. Additionally, 41 other variants were associated with PC1–5 (*P*  <  0.05). With respect to rare variant analysis, variants within the *EP300*, *NCOR2*, and *PSEN2* gene showed an association with MP (*t*   < 0 .05).

**Conclusions:**

An association between *NOTCH* signaling pathway genes and MP has been identified.

**Supplementary Information:**

The online version contains supplementary material available at 10.1186/s13005-021-00268-0.

## Background

Mandibular prognathism (MP) is characterized by excessive mandibular growth with or without deficient maxillary growth [1]. It causes an unpleasant facial profile and decreases masticatory efficiency [2, 3]. Prevalence of MP varies according to population; it is higher in Asians (approximately up to 15%) and lower in Caucasians (approximately 1%) [4]. Environmental factors have been found to contribute to the development of MP, such as enlarged tonsils, difficulty in nasal breathing, congenital anatomic defects, and diseases of the pituitary gland [5]. A familial aggregation phenomenon suggests that heredity plays a major role in the etiology of MP. The inheritance pattern of MP is complex, and controversial findings have been reported suggesting an autosomal recessive inheritance, an autosomal dominant inheritance, a dominant inheritance pattern with incomplete penetrance, or a polygenic threshold model for inheritance [6].

Genetic studies have focused on MP. A few genome linkage scans and a genome wide association study (GWAS) identified some loci related to MP, including the first susceptibility loci,1p36, 6q25, and 19p13.2 in Korean and Japanese families [6]; 11q22, 12q13.3, and 12q23 in Hispanic families; and 14q24.3–31.2 in Han Chinese pedigrees [7, 8]. Cartilage matrix protein (*Matrilin-1*) and erythrocyte membrane protein band 4.1 (*EPB41*) within the 1p36 locus were identified as potential genes underlying MP [9, 10]. Myosin 1H (*MYO1H*) located on 12q24.11, which is near 12q23, was suggested to be related to MP [11]. Rio de Janeiro et al. demonstrated that *MYO1H* (rs10850110 A\G) was associated with an increasing risk for the mandibular prognathism phenotype in a Brazilian population [12]. A microsatellite genome-wide association study in a Japanese population suggested that 2 loci--1q32.2 and 1p22.3, and two genes--*PLXNA2* and *SSX2IP,* were associated with MP [13]. Recently, our group has been conducting genetic studies of MP and has identified a novel mutation in *FGF23*, c.35C > A, that is strongly associated with MP [14]. Even considering these findings, the genetic factors associated with MP are not fully understood, leading to motivation to search for new candidate genes.

MP is a developmental malformation underling a craniofacial osteogenesis disorder. The genetic mechanisms of craniofacial development have been elucidated, and the *FGF, BMP, SHH,* and *NOTCH* signaling pathways as well as many other signal pathways play critical roles [15]. The mandibular condyle, a growth center of the mandible, undergoes endochondral bone formation that is controlled by regulatory factors. Cells in its proliferative layer express the transcription factor Sox9. Sox9 then regulates chondrocytes to synthesize type II collagen, and then, chondrocytes progress towards hypertrophy and secrete type X collagen for the hypertrophic cartilage destined for endochondral ossification [16].

The *NOTCH* signaling pathway plays a key role in skeletal development and bone remodeling. It regulates morphogenesis during development by establishing and maintaining cellular boundaries that subdivide an originally homogeneous tissue field into distinct cell populations. In vertebrates, *NOTCH* signal establishes the anterior–posterior polarity of each somite [17–19]. In mandibular condylar cartilage (MCC), *NOTCH* receptors and ligands are localized to the chondroprogenitor and perichondrial layers. *NOTCH* signaling promotes angiogenesis in the bone endothelium, which involves the paracrine release of Sox9 and VEGF by endothelial cells that are required for chondrocyte maturation [20]. Lower expression of *NOTCH* in the MCC decreases the proliferation of chondrocytes and promotes differentiation (Sox9 expression) [21]. Overexpression of the Notch1 intracellular domain (N1-ICD) significantly increases *BMP2*-mediated induction of alkaline phosphatase (ALP) activity and calcification of human aortic smooth muscle cells [22]. Other genes of the *NOTCH* signaling pathway, including *Lfng, Hey1,* and *Hes1,* can be regulated by *BMP-2* and *TGF-β* as well [23]. Forward reposition of the mandible with functional appliances can trigger the expression of *Ihh* and *Pthlh*, which promote mesenchymal cell differentiation and proliferation. Meanwhile, the IHH and PTHLH proteins act as mediators of mechanotransduction to promote growth of the cartilage [24]. Mice with cartilage-specific deletion of *Notch* display a strong impairment in columnar zone chondrocyte responsiveness and significant incensement of perichondrial osteoblast responsiveness to *IHH*, which coincided with an advanced osteoblast differentiation and bone formation phenotype [25, 26].

Gain- or loss-of-function mutations in *NOTCH* signaling pathway genes result in different types of skeletal diseases. These mutations are associated with spondylocostal dysostosis, spondylothoracic dysostosis and recessive brachydactyly, diseases characterized by skeletal patterning defects. *Notch* is also highly expressed in osteosarcoma and in breast cancer cells that form osteolytic bone metastases. *Wnt1Cre;Rosa*^*Notch*^ embryos exhibitneural tube closure defects, along with exencephaly and micrognathia [27]. Hajdu-Cheney syndrome, which is driven by the production of a stabilized *NOTCH2* lacking a functional PEST (peptide sequence that is rich in proline (P), glutamic acid (E), serine (S), and threonine (T)) degradation domain, is caused by gain-of-function mutations in *NOTCH2* [28]. This disorder is characterized by short stature, bowing of the long bones, vertebral anomalies and facial features including hypertelorism, bushy eyebrows, micrognathia, small mouth with dental anomalies and low-set ears. Alagille syndrome is a genetic disorder clinically defined by hepatic bile duct paucity, cholestasis, cardiac, skeletal, and ophthalmologic manifestations [28]. It is caused by haploinsufficiency of *JAG1* (94% of patients) or by mutations in *NOTCH2* (2% of patients) and is considered to be a *Notch* loss-of-function phenotype [29]. Adams-Oliver syndrome is diagnosed based on terminal transverse limb malformations, an absence of skin and a partial absence of skull bones. This rare genetic disorder can be autosomal dominant, autosomal recessive or caused by de novo mutations. The autosomal dominant forms are caused by mutations in *NOTCH1, RBPJ* or *DLL4*, all of which are *NOTCH* pathway components [30, 31]. It is reasonable to hypothesize that the variants of genes in the *NOTCH* signaling pathway play an important role in MP pathogenesis.

Rare genes causing complex diseases provide wedges of understanding to crack open whole metabolic pathways and uncovering new candidate genes for further genetic disease study [32]. Genes that are linked to rare syndromes can provide insight into the comprehension of isolated traits [12].Rare diseases can serve as models for genetic susceptibility of more common traits in the population [33],which gave our group the idea that there is a relationship between the *NOTCH* signaling pathway and maxillofacial malformation. The purpose of this study was to systematically identify variants of the genes in the *NOTCH* signaling pathway that predispose one to MP in the general Chinese population.

## Materials and methods

### Participants

This case-control study included 199 MP patients (mean age, 23.6 ± 3.2 yrs.; 86 males) and 197control individuals with Class I occlusion (

mean age, 26.8 ± 2.6 yrs.; 86 males). All subjects were recruited from registered patients who underwent orthodontic treatment from January 2015 to September 2016, This study was approved by the Human Ethics Committee and was conducted according to Declaration of Helsinki principles, and all participants gave written informed consent. The statistical power was computed using Piface (Version 1.76, https://homepage.divms.uiowa.edu/~rlenth/Power/). A positive allele can be detected when the odds ratio (OR) is greater than 3 with the sample size in this study, under the assumption of 1% (minor allele frequency) MAF, 5% type I error rate (α), and 80% statistical power.

The occlusal relationship of individuals was evaluated with a dental study model or visual inspection and was confirmed by digital tracing of a lateral cephalogram. Patients with facial trauma, congenital abnormalities (such as cleft lip and palate), or endocrinological diseases were not included in the study. Those that had undergone previous orthodontic treatment were also excluded from the present study. All the participants were of unrelated Chinese Han ethnicity.

The assessment of the eligible subjects consisted of diagnosing by digital tracings of lateral cephalograms, which were taken using dental X-ray equipment (Veraviewepocs X550, Kyoto, Japan).

The inclusion criteria for MP were defined as a cephalometric ANB angle (Point A-nasion- Point B) of centric jaw relationship less than 0° [6] and a negative Wits appraisal greater than − 2.0 mm [11]. The inclusion criteria for normalskeletal Class I was an ANB angle range from 0.3 to 4.8 degrees and a Wits appraisal between − 1.3 and 2.4 mm (Table 1). All participants provided a blood sample from which DNA could be extracted. Genomic DNA from EDTA-anticoagulated peripheral blood was extracted using a QIAamp DNA Blood Kit (QIAGENE GmbH, Hilden, Germany) according to the manufacturer’s instructions. All the samples were stored at <− 80 °C until analysis [34].

**Table 1:** Demographical characteristics of the cases and controls

	Male/Female	Mean age (SD)	Mean ANB(°, SD)	Mean wits appraisal (mm, SD)
Case	39/57	20.49 ± 6.02	− 3.14 ± 2.23	3.95 ± 2.89
Control	44/59	21.24 ± 6.19	2.93 ± 1.32	−4.24 ± 3.89

*ANB* angle point A-Nasion-point B, *SD* standard deviation

### Cephalometric analysis

Two independent orthodontists performed cephalometric tracing using NemoCeph NX software (version 6.0, Nemotec, Madrid, Spain) at 2-week intervals. Twenty-seven skeletal landmarks and 9 soft landmarks were traced on a lateral cephalogram of each participant (Table S1). Then, sixty-one cephalometric parameters were digitally generated and used for phenotyping study (Table S2). The interrater and intrarater agreement was then tested by an intraclass correlation method as described previously [35].

### Targeted region sequencing and data analysis

In this study, the coding and flanking regions of the 27 genes (total length: approximately 151,344 bp) in the *NOTCH/Delta1* signaling pathway were selected and sequenced (Table S3). A customized NimbleGen capture array (Roche-NimbleGen Inc. Madison, WI, Custom probes details are in Table S4) was used to capture the targeted regions according to the manufactory’s protocols. Then, the sequencing was performed using an Illumina Hiseq2000 platform (Illumina Inc., San Diego, CA). The original reads were then aligned to the human reference genome (hg19) using the Burrows-Wheeler Alignment tool v0.7.1 (http://maq.sourceforge.net) to generate a binary sequence alignment/mapping file with various mapping information. Then, duplicate reads were removed, and alignments were processed with Picard v1.137 (Https://github.com/broadinstitute/picard/releases) and the Genome Analysis Toolkit v3.4–46. The coverage, average quality, and global depth of the alignment read according to the stack file were generated with SAMtools v1.2 (*P* < 0.05). Variants were called using SNPTools and annotated using the ANNOVAR software package (http://www.openbioinformatics.org/annovar/). Possible pathogenic effects of the missense mutations were evaluated using MutationTaster software (http://www.mutationtaster.org). Indels (insertion/deletion) were verified manually. Six randomized samples (3 cases and 3 controls) were analyzed (Figure S1), and Sanger sequencing of the positive SNPs in this study was also carried out.

### Statistical analysis

Each SNP was evaluated independently in the cases and the controls for Hardy–Weinberg equilibrium (HWE) analysis (http://www.oege.org/software/hwe). For the common variation (MAF ≥ 1%), the genotyping distributions and allele frequencies of the SNPs between the cases and controls were compared using a Pearson chi-square test or Fisher’s exact test (when the expected count was less than 5). The effect of variants on the MP odds ratio (OR) and 95% confidence interval (CI) was tested by logistic regression analysis. After age and gender adjustments, a linear regression analysis was performed to determine the association between each cephalometric parameter and common variants. Principle component analysis was used to decrease the dimensions of the phenotypes. Principle components (PCs) explaining more than 5% of the total variance of the cephalometric parameters data were used to test the association with variants by linear regression. For rare variants (MAF < 1%), a *t*-test was performed to compare cumulative exonic variants in each gene region of cases and controls. In addition, the Combined Annotation Dependent Depletion (CADD) score was used as a weight factor in this test. All statistical analyses were performed using SAS version 9.2 (SAS Institute Inc., Cary, NC) with a double-headed *P*-value < 0.05 considered statistically significant.

## Resultss

In our study, 27 skeletal landmarks and 9 soft landmarks were traced on a lateral cephalogram of each participant at least 2 weeks apart (by one of the co-first authors) to assess the intrarater reliability. A subsample of 15 cephalometric radiographs was chosen randomly and traced by the other rater (the other co-first author) to assess interrater reliability. The reliability of the landmark location was determined by intraclass correlation methods (ICC). Our results showed that the intrarater reliability ranged from ICC = 89.21% to ICC = 99.98%, while the interrater reliability ranged from ICC = 86.33% to ICC =99.57%. Both values were generally acceptable (≥85%) [35].

### Targeted sequencing data

The average sequencing coverage was 67× (interquartile range 43–87×). The concordance of the variants called in duplicate samples was more than 99%. The variants with calling rates less than 95% or inconsistent with Hardy–Weinberg equilibrium (*P* < 0.01) in the control group were removed. Based on the MAF of the variants tested in the control group, the retained variants were then classified into 2 groups: common variants (MAF ≥ 0.01) and rare variants (MAF < 0.01). Overall, 1520 variants were identified across all sequenced individuals in the targeted regions, including 337 common variants and 1183 rare variants.

### Association analysis of common variants

**Fig. 1:**
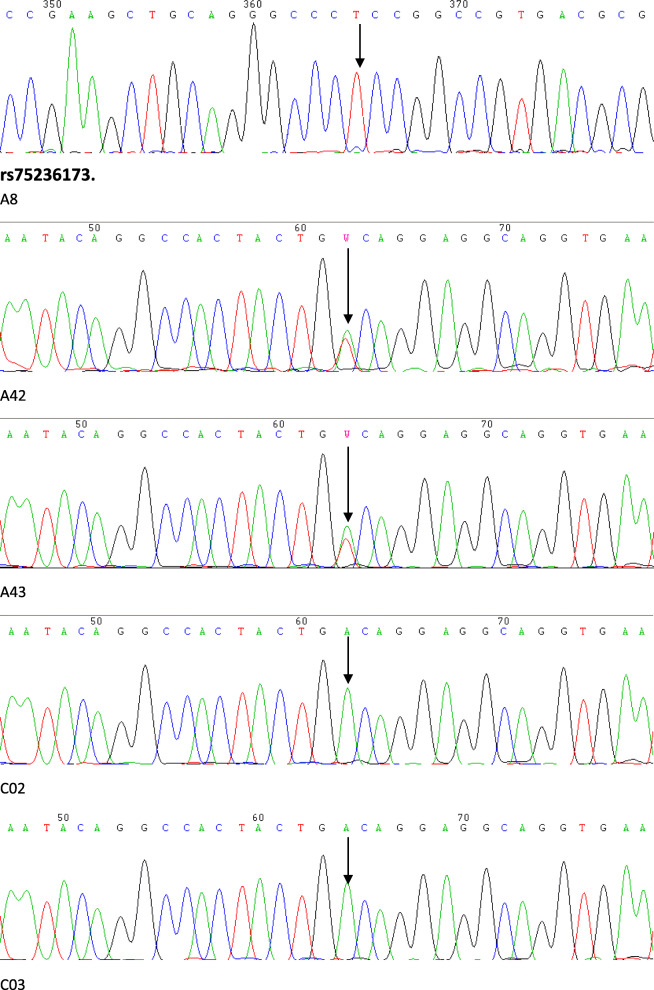
Three SNPs (rs1051415 in an exonic region of *JAG1*; rs75236173 in the 3’-UTR of *NUMB*; rs1044006 in an exonic region of *NOTCH3*) in six randomly selecting samples (including three cases and three controls) were validated by Sanger sequencing. The three cases were A8, A42 and A43, while the three control samples were C02, C03 and C8. The black arrows represent the polymorphism sites

Among the 337 common variants, we presented only those variants significantly associated with MP and PC that explained more than 5% of the facial variation. The Sanger sequencing results for the positive SNPs in this study were in accordance with the raw results (Fig. 1). No common variants within the 27 genes were significantly associated with MP after Bonferroni correction for multiple testing (cut-off *P* value = 0.05/337 = 1.48 × 10^− 4^). Only 6 SNPs reached nominal significance (*P* < 0.05), including rs415929, rs520688, and rs423023 in an exonic region of *NOTCH4*; rs1044006 in an exonic region of *NOTCH3*; rs1051415 in an exonic region of *JAG1;* and rs75236173 in the 3′-UTR of *NUMB.* The genotypic and allelic frequencies at rs415929, rs423023, rs520688, rs1044006, rs1051415, and rs75236173 were significantly different between the case and control groups. The C allele of rs415929, the C allele of rs423023, the C allele of rs520688, the C allele of rs1044006, the T allele of rs1051415, and the T allele of rs75236173, increased the risk of MP, with OR ratios of 1.503(1.039, 2.176), 1.480(1.022, 2.144), 1.445(1.001, 2.085), 0.711(0.506, 0.9989), 1.708(1.044, 2.784), and 1.417(1.007, 1.995), respectively (Table 2).

**Table 2:** The associations between SNPs identified in NOTCH signaling pathway genes and MP

SNP-Gene-Function	Genotype/allele				Logistic regression		Statistical Power(%)
		Cases	Controls	*P*	OR(95% CI)	*P*	
rs415929-*NOTCH4*-EXON	TT	126 (63.3)	142 (72.1)	0.075	1.503 (1.039–2.176)	0.030	39.8
	TC	64 (32.2)	52 (26.4)				
	CC	9 (4.5)	3 (1.5)				
	T:C	79.4:20.6	85.3:14.7	0.280			
rs105415-*JAG1*-EXON	CC	156 (78.4)	171 (86.8)	0.087	1.708 (1.044–2.784)	0.031	33.91
	CT	40 (20.1)	24 (12.2)				
	TT	3 (1.5)	2 (1.0)				
	C:T	88.4:11.6	92.9:7.1	0.280			
rs423023-*NOTCH4*-EXON	GG	126 (63.3)	142 (72.1)	0.097	1.480 (1.022–2.144)	0.037	32.2
	GC	65 (32.7)	52 (26.4)				
	CC	8 (4.0)	8 (4.1)				
	G:C	79.7:20.4	85.3:14.7	0.290			
rs7523617-*NUMB*-UTR3	AA	111 (55.8)	132 (67.0)	0.066	1.417 (1.007–1.995)	0.045	30.05
	AT	79 (39.7)	57 (28.9)				
	TT	9 (4.5)	8 (4.1)				
	A:T	75.6:24.4	81.5:18.5	0.310			
rs520688-*NOTCH4*-EXON	TT	126 (63.3)	141 (71.6)	0.140	1.445 (1.001–2.085)	0.049	29.06
	TC	64 (32.2)	52 (26.4)				
	CC	9 (4.5)	4 (2)				
	T:C	79.4:20.6	84.8:15.2	0.320			
rs1044006-*NOTCH3*-EXON	CC	134 (67.3)	116 (58.9)	0.170	0.711 (0.506–0.999)	0.049	29.01
	CT	55 (27.6)	65 (33.0)				
	TT	10 (5.1)	16 (8.1)				
	C:T	81.2:18.8	75.4:24.6	0.320			

Some associations between common variants and each cephalometric parameter were also detected. rs3125001 in *NOTCH1* was negatively associated with inferior facial height (*P* < 0.01). rs372504208 in *NOTCH2* is a frameshift deletion (c.17_18delCC). It was found to be negatively associated with articular angle (*P* < 0.05). rs1044009 in *NOTCH3* was associated with the APDI index (NP-AB) and the ANB angle (*P* < 0.05). rs386591752 in *NOTCH4* was associated with the ANB angle, wits appraisal, APDI index, facial convexity and overjet (*P* < 0.01).rs1051415 in *JAG1* was associated with the anterior-posterior facial height ratio (*P* < 0.01). rs2272591 and rs10149229 in *JAG2* were negatively correlated with mandibular body length (*P* < 0.01). rs1057744 in*JAG2* was negatively correlated with facial angle and the Y axis *(P* < 0.05). rs2304223 in *DLL3* was negatively associated with facial angle (*P* < 0.05). rs20551 in *EP300* was negatively associated with mid-face length (*P* < 0.05) (Table 3).

**Table 3:** Association between common variants and cephalometric parameter

SNP-Gene-Function	A1/A2	Phenotype parameters	BETA	SE	*P*
rs1051415-JAG1-exon	C/T	Anterior-posterior facial height ratio (%)	2.50	0.86	0.0041
rs2272591-JAG2-exon	A/G	Mandibular body length (mm)	−2.12	0.81	0.0093
rs10149229-JAG2-exon	A/G	Mandibular body length (mm)	−2.80	0.82	0.00074
rs1057744-JAG2-exon	C/T	Facial angle (°)	34.08	15.41	0.028
		y axis (°)	1.06	0.45	0.020
rs386591752-NOTCH4-exon	T/C	ANB angle (°)	−1.58	0.48	0.0012
		Wits appraisal (mm)	−2.42	0.72	0.0010
		APDI index(°)	1.79	0.64	0.0054
		Facial convexity(°)	−3.65	1.10	0.0011
		Overjet (mm)	−1.69	0.53	0.0017
rs372504208-NOTCH2-exon	CGG/C	Articularangle (S-Ar-Go) (°)	−1.89	0.83	0.025
rs1044009-NOTCH3- exon	G/A	APDI index(°)	−1.25	0.54	0.022
rs2304223-DLL3-intron	C/G	ANB angle (°)	−0.73	0.34	0.033
		Facial angle (°)	−51.70	24.83	0.039
rs20551-EP300-exon	A/G	Midface length (mm)	−2.91	1.44	0.045

**Fig. 2:**
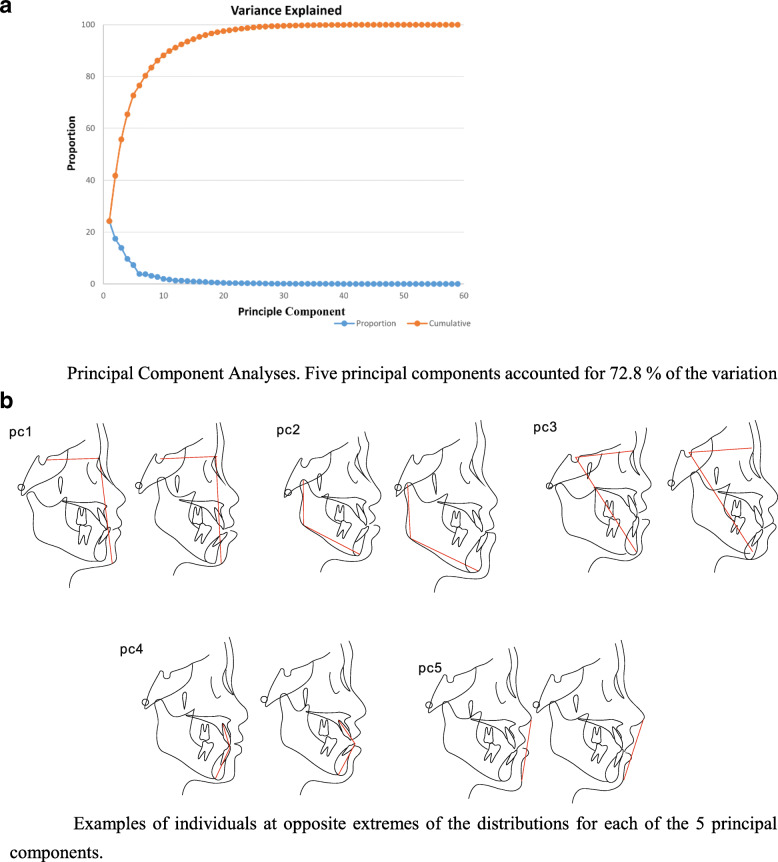
Examples of individuals at opposite extremes of the distributions for each of the 5 principal components. PC1 explained 24.3% of the observed facial variation and corresponded to variations within the sagittal mandibular position toward maxilla. PC2 explained 17.5% of the observed facial variation and corresponded to mandibular shape and size. PC3 corresponded to the vertical and sagittal positions of the mandible relative to the cranial base. PC4 corresponded to the protrusion and inclination of the lower and upper incisors and explained 10% of the observed facial variation. PC5 corresponded to the protrusion of facial soft tissue and accounted for 7.3% of the variation

The results of the PCA revealed that 5 PCs accounted for 72.8% of the total variance, and each of them represented 24.3, 17.5, 14, 9.7, and 7.3% of the total variance, respectively (Fig. 2a, Table S5). A common variant (rs1051415; *P*  < 0.01) in an exonic region of the *JAG1* gene associated with PC1 was highly suggestive of MP. This component correlated with the sagittal mandibular position toward maxilla ranging from a posterior positioned mandible to an anterior positioned mandible. Another common variant (rs10149229; *P* < 0.01) in an exonic region of *JAG2* was associated with PC4, which captured the protrusion and inclination of the lower and upper incisor. This variant was also associated with PC2, which mainly captured mandibular shape and size ranging from a short mandibular length, a short middle and posterior facial height to a long mandibular length, and a long middle and posterior facial height, although the associations were not significant after multiple corrections. rs915894 (*P* < 0.01) in an exonic region of *NOTCH4*, which is a nonsynonymous SNV, was associated with PC3 and mainly referred to the vertical and sagittal positions of the mandible relative to the cranial base. (Fig. 2b).

We also detected 4 other SNPs associated with PC1, 11 other SNPs associated with PC2, 6 other SNPs associated with PC3, and 16 other SNPs associated with PC4, with nominal significance (*P* < 0.05). In addition, 6 other SNPs associated with PC5, which captured the protrusion of facial soft tissue, were also identified at a *P*  <  0.05 significance level (Table 4).

**Table 4:** Association between common variants and 5 PCs

Trait	SNP	A1/A2	Gene	Fun	Beta	SE	*P*
PC1	rs1051415	C/T	JAG1	Exon/syn	-0.7298	0.1986	0.0003608
PC1	rs7104987	A/G	MAML2	UTR3	0.2938	0.1219	0.0175
PC1	rs8383	C/AGT	PSEN2	Intronic	0.2925	0.1303	0.02666
PC1	rs7930268	T/CG	MAML2	UTR3	0.2802	0.1254	0.02742
PC1	rs2304214	C/GT	DLL3	Exon/syn	-0.4739	0.2129	0.02793
PC1	rs2304223	C/G	DLL3	Intronic	-0.4739	0.2129	0.02793
PC2	rs1044009	G/AC	NOTCH3	Exon/nonsyn	-0.2354	0.1136	0.0404
PC2	rs741859	C/T	JAG2	UTR3	-0.3261	0.1231	0.009212
PC2	rs957578869	G/T	JAG2	UTR3	0.4257	0.1489	0.005034
PC2	rs1177358515	G/T	EP300	Intronic	0.4909	0.1887	0.01049
PC2	rs10149229	A/CG	JAG2	Intronic	-0.3172	0.1222	0.01068
PC2	rs2272591	A/GT	JAG2	Intronic	-0.3083	0.1211	0.01223
PC2	rs6705408	C/T	ADAM17	UTR3	-0.4671	0.1949	0.01812
PC2	rs2274185	C/G	NCSTN	Intronic	-0.3322	0.156	0.03528
PC2	rs1106317	A/GT	NCOR2	Intronic	0.2442	0.1159	0.03726
PC2	rs9972231	C/AGT	JAG2	Intronic	0.3294	0.1565	0.03748
PC2	rs117649295	G/A	LFNG	UTR3	-0.4966	0.2413	0.04184
PC2	rs28386899	G/A	RFNG	UTR5	0.3577	0.1753	0.04354
PC3	rs165935	C/T	PSEN1	UTR3	-0.3067	0.1388	0.02915
PC3	rs6563	A/CG	NOTCH1	UTR3	0.3215	0.1519	0.03641
PC3	rs1055834488	G/AC	JAG1	UTR5	-0.3348	0.1637	0.04304
PC3	rs10423702	T/C	NOTCH3	Intronic	0.3721	0.1822	0.0434
PC3	rs1044009	G/AC	NOTCH3	Exon/nonsyn	0.3327	0.1496	0.02802
PC3	rs1043996	G/A	NOTCH3	Exon/syn	0.3543	0.1314	0.008049
PC3	rs915894	T/G	NOTCH4	Exon/nonsyn	0.3462	0.1313	0.009514
PC4	rs1033583	T/G	DLL1	UTR3	-0.4583	0.1541	0.003572
PC4	rs2272591 *[*	A/GT	JAG2	Intronic	-0.3459	0.1345	0.01134
PC4	rs3734776	C/T	DLL1	Intronic	-0.3278	0.1281	0.01176
PC4	rs3741513	T/A	NCOR2	Exon/syn	-0.4064	0.1646	0.01499
PC4	rs3134942	G/T	NOTCH4	Exon/syn	-0.7543	0.3105	0.01664
PC4	rs3134930	C/T	NOTCH4	Intronic	-0.2993	0.1259	0.01907
PC4	rs741859	C/T	JAG2	UTR3	-0.321	0.1376	0.02139
PC4	rs1044507	A/C	NOTCH4	Exon/syn	-0.7504	0.3249	0.02266
PC4	rs7931870	A/G	MAML2	Exon/syn	-0.3707	0.1676	0.02891
PC4	rs2243396	C/GT	DTX1	Intronic	-0.3267	0.149	0.03034
PC4	rs3134798	G/ACT	NOTCH4	Intronic	0.4612	0.2113	0.03109
PC4	rs114763	C/GT	SNW1	Exon/syn	-0.2856	0.1331	0.03401
PC4	rs422951	T/C	NOTCH4	Exon/nonsyn	-0.423	0.1976	0.03435
PC4	rs443198	A/GT	NOTCH4	Exon/syn	0.2815	0.1332	0.03666
PC4	rs3823301	C/T	DLL1	UTR5	-0.3524	0.127	0.006446
PC4	rs10149229	A/G	JAG2	Exon/syn	-0.3708	0.1353	0.007108
PC4	rs915894	T/G	NOTCH4	Exon/nonsyn	0.2567	0.1277	0.04663
PC5	rs79129905	G/A	LFNG	UTR3	-0.4519	0.1827	0.01481
PC5	rs5758235	T/C	EP300	Intronic	-0.3857	0.1426	0.007857
PC5	rs753573114	C/AT	EP300	Intronic	-0.4273	0.1431	0.003452
PC5	rs3818120	G/A	EP300	intronic	-0.3576	0.1395	0.01162
PC5	rs20552	T/ACG	EP300	Exon/syn	0.3761	0.1474	0.01203
PC5	rs17002316	T/C	EP300	intronic	-0.3857	0.1426	0.007857

### Association analysis of rare variants

Compared with the controls, rare variants within the *EP300, NCOR2, PSEN2* genes showed association with MP (*t*   < 0 .05) (Table S6).

## Discussion

It is widely believed that genetic components play an important role in MP. To date, numerous chromosomal loci implicated in MP pathogenesis have been reported, and also a host of genes that predispose MP, such as *EPB41, MATN1, COL2A1, MYO1H, TGFB3, LTBP2, ADAMTS1, DUSP6, FGFR2,* and *FGF23*. Most of these studies were based on family linkage studies. However, the polygenic nature of MP makes it possible to study its genetic mechanism using a case-control design.

Although genetic linkage analysis and association studies have identified many genes and loci associated with MP, the genes underlying the risk of MP in the general population remain elusive, prompting our search for new candidate genes. The *NOTCH* signaling pathway has been suggested to participate in craniofacial development and the regulation of *TGFB3, FGFR2,* and *FGF23*. We speculate that it is also involved in the etiology of MP. In the current study, we aimed to identify the association between variants in *NOTCH* signaling pathway genes and MP in MP cases and controls using a targeted sequencing strategy. We found some suspicious variants in these genes associated with MP.

According to association analysis of common variants, only 6 SNPs reached nominal significance (*P* < 0.05), including rs415929, rs520688, and rs423023 in an exonic region of *NOTCH4*; rs1051415 in an exonic region of *JAG1;* and rs1044006 in an exonic region of *NOTCH3*. As Bonferroni adjustment was not applied to most of the pairwise comparison, we acknowledge that the current significant findings are only suggestive and may be affected by Type I error.

Human JAGGED1 is the ligand for the NOTCH1 receptor. In a Mexican population, rs1051415 in the *JAG1* gene was associated with Alagille syndrome (OMIM#118450),which presents characteristic facial features including a pointed chin [36]. In this study, rs1051415 in an exonic region of *JAG1* was associated with MP (*P* < 0.05). It was also associated with the anterior-posterior facial height ratio (*P* < 0.01). *jagged-notch* signaling contributes to the dorsal mandibular arch domain by repressing expression of genes associated with ossification. Deleting *Jagged1* in the cranial neural crest (CNC) causes (*Wnt1-cre, Jag1 Flox/Flox)* mice die at postnatal day 30 due to an inability to masticate, owing to jaw misalignment and poor occlusion, recapitulating the midfacial hypoplasia phenotype of Alagille syndrome [37]. Conditional inactivation of *Jag1* in mouse NCCs leads to the development of a shortened maxilla [38]. Our results are consistent with these findings suggesting that *JAG1* is associated with MP. The mutations in *JAG1*were associated with unilateral coronal craniosynostosis in humans. As the temporal and spatial patterns of *NOTCH* signaling expression are markedly different in the posterofrontal and sagittal sutures, Notch may contribute to craniosynostosis and then to the craniomaxillofacial growth [39].

rs10149229 in exon 26 of *JAG2* was correlated with PC4, which captured the protrusion and inclination of the lower and upper incisor (*P* < 0.01). Four other SNPs in *JAG2* were also identified: rs145952626 and rs741859in the 3′-UTR of *JAG2* were correlated with PC2 (*P* < 0.01), which mainly correlated with a longer mandible length and a longer middle and posterior facial height. In a Brazil population, a strong association was observed by haplotype analyses containing rs1057744 polymorphism in cleft lip with or without cleft palate [40]. Mice with inactivated *Jagged2* exhibit craniofacial defects including cleft palate [41]. Although mutations in the *JAG2* gene have not been linked to any specific human diseases, homozygous *Jag2* null mice display severe craniofacial defects, including cleft palates and fusion of the tongue with the palatal shelves. The potential function of the *JAG2* gene in maxillofacial development needs to be further studied.

rs10521 in an exonic region of *NOTCH1* was associated with PC1 *(P* < 0.05). High levels of activated *Notch1* were observed in the differentiating oral periderm and the lateral mandibular and maxillary processes [42]. *NOTCH1* is localized primarily in the chondroprogenitor layer of the mandibular condylar cartilage (MCC). Disruption of *NOTCH* signaling in MCC explants decreased proliferation and increased chondrocyte differentiation, and the actions of *FGF-2* in MCC are mediated in part by *NOTCH* signaling [43, 44].

rs372504208 in exon 1 of *NOTCH2* is a frameshift deletion (c.17_18delCC) that was predicted to be disease-causing according to MutationTaster [45]. It was negatively associated with articular angle (*P* < 0.05). *NOTCH2* is known to be important for vertebrate cranial morphogenesis, especially mandible and tooth development [28]. Mutations in *NOTCH2* are responsible for Hadju–Cheney syndrome (OMIM #102500) and Alagille syndrome [46]. The contribution of *NOTCH2* to MP needs to be elucidated in the future.

rs1044006 in an exonic region of *NOTCH3* was associated with MP(*P* < 0.05)*.* rs1044009 in an exonic region of *NOTCH3* was associated with PC2 (*P* < 0.05), which mainly indicated a longer mandible length and a longer middle and posterior facial height. This SNP was also negatively associated with APDI index, ANB and SNB angle (*P* < 0.05). Low masticatory loading inhibits the development of condylar cartilage and decreases expression of *notch-1, notch-3, jagged-1* and *delta-like-1* in rabbits [47]. Thus, there may be a relationship between condyle development and the *NOTCH* signaling pathway. In the mandibular torus, increased osteogenic differentiation of mesenchymal stem cells (MSCs) was associated with the suppression of *NOTCH3* signaling and its downstream target genes, which may contribute partly to bone outgrowth in mandibular torus [48]. As MP is an overgrowth of bone, it is reasonable to speculate *NOTCH3*participates in the etiology of MP [49]. The potential function of the *NOTCH3* gene in maxillofacial development needs to be studied further.

rs415929, rs520688, and rs423023 in an exonic region of *NOTCH4* were associated with MP(*P* < 0.05). rs915894 in *NOTCH4* was associated with PC3,which mainly referred to the position of the mandible relative to the cranial base. rs386591752 in exon 6 of *NOTCH4* was negatively associated with the relative relationship between the maxilla and the mandible, including the ANB angle, wits appraisal, APDI, facial convexity and overjet (*P* < 0.01), which are important clinical diagnosis standards for Class I, Class II, and Class III skeletal facial patterns [50]. *NOTCH4* is a transmembrane protein that regulates interactions between adjacent neurons. Multiple genetic association studies have associated *NOTCH4* with rheumatoid arthritis (RA) [51]. rs915894 in exon 3 of *NOTCH4* is modestly significantly associated with RA (*P* < 0.01) [52]. The temporomandibular joint (TMJ) is a synovial joint. Being a target of RA, TMJ shows aggrecan degradation in the cartilage when RA occurs [53, 54]. As it contributes to cartilage injury and rebuilding, *NOTCH4* may play a role in condyle cartilage metabolism, which needs further research. While *NOTCH4* has been explored in regards to angiogenesis [55] and tumorigenesis [56], we think it is necessary to verify the function of *NOTCH4* in craniofacial development. In addition, our results showed rs2304223 in an intron of *DLL3* was negatively associated with facialangle (*P* < 0.05). It was also associated with PC1 (*P* < 0.05), which depicted the sagittal mandibular position toward maxilla ranging from posterior positioned mandible to anterior positioned mandible. *DLL3* encodes a member of the delta protein ligand family. *Dll3-Notch1* double heterozygous mice display remarkable reduction of mandibular height and elongation of the maxillary hard palate [57]. Mutations in *DLL3* cause autosomal recessive SCDO1 (OMIM**#**277300).

A frameshift deletion in exon 2 of *LFNG* (c.135_138del:p.W45fs) was predicted to be disease causing by MutationTaster [58]. This mutation was negatively associated with facial taper (*P* < 0.05). It was also associated with PC3 (*P* < 0.05), which mainly referred to the vertical and sagittal positions of the mandible relative to the cranial base. *LFNG* is a member of the fringe gene family that encodes evolutionarily conserved glycosyltransferases that act in the *NOTCH* signaling pathway to define boundaries during embryonic development. While their genomic structure is distinct from other glycosyltransferases, these proteins lead to the elongation of O-linked fucose residues on the NOTCH protein, which alters *NOTCH* signaling. An *LFNG* product is predicted to be a single-pass type II Golgi membrane protein. Mutations in *LFNG* have been associated with autosomal recessive spondylocostal dysostosis type 3 (SCDO3) (OMIM**#**609813). In SCDO3 patients, all vertebral bodies appear to show more severe segmentation defects [59]. As *LFNG* may contribute to bone development, further research would be needed to identify the effect on craniofacial morphogenesis.

There are common variants with moderate effects and rare variants that have a great influence on the complex traits of genes. In the case of a moderate effect size with an MAF of less than 0.5%, an association study using total “mutation load” composite tests that compare the frequency of mutation to potentially similar functional effects in cases and controls is needed. Low frequency variants could have a substantial effective without showing clear Mendelian isolation and could substantially contribute to missing heritability [12]. In this research, to explore the effect of rare variants of genes in the *NOTCH* signaling pathway in MP pathogenesis, burden tests were performed to compare the cumulative exonic variants in each gene region of cases and controls. Combined Annotation Dependent Depletion (CADD) is a quantitative score integrating many diverse annotations together, which relates to diversity of alleles, functionality, pathogenicity, and the severity of diseases. The CADD score power weight was also included with our burden tests to verify different effects of each variant. This was the first time that burden testing was combined with CADD score, which was an effective method for evaluating the association between genes and the etiology of mandibular prognathism. Then the summing over the cumulative score in all loci of each gene in cases and controls was compared by independent-samples T test.

“association analysis of rare variants” may also be affected by Type I error. In this study, rare variants in *EP300* were related to MP (*P* < 0.05). In addition, rs20551 in exon 15 of *EP300* was negatively associated with mid-face length (*P* < 0.05). *EP300* is vital in cell proliferation and differentiation. It mediates *cAMP*-gene regulation by binding specifically to phosphorylated CREB protein. *EP300* has also been identified as a co-activator of *HIF1A*, which plays a role in the stimulation of hypoxia-induced genes such as *VEGF*. Mutations in *EP300* are a rare cause of Rubinstein-Taybi syndrome (RSTS) (OMIM**#**180849). RSTS is mainly characterized by growth delays, craniofacial features (i.e., downslanting palpebral fissures, pouting lower lip, dental crowding, micrognathia and dysplastic), and skeletal abnormalities including broad or duplicated distal phalanges of thumbs and halluces [60].

According to the recommendations of a genetic association study, we applied a strict multiple testing correction. However, the Bonferroni adjustment can lead to a loss of true association because Class I errors cannot be reduced without increasing Type II errors, which does not guarantee a careful explanation of the results [61]. Although most of these effects didn’t survive multiple testing correction, our results may also be suggestive. Mandibular prognathism (MP) has long been considered a complicated maxillofacial disorder, with both genetic and environmental factors contributing to its etiology. Now, it is accepted by most researchers that MP is a polygenic disorder. Polygenic inheritance refers to the inheritance of a phenotypic trait that can be attributed to two or more susceptibility genes. Our results indicated that the main effect gene may not be included in the NOTCH signaling pathway. Future studies with larger sample sizes, more comprehensive genome coverage, and in other population are required to replicate these findings. We will continue to collect more samples, which may take us additional time.

## Conclusion

The genetic mechanisms of MP are complex. In this study, we identified some variants in the *NOTCH signaling pathway* that may be associated with MP. We found that rs1051415 in an exon of *JAG1* was significantly related to PC1 (*P*  =3.608 × 10^− 4^), which corresponded to the sagittal mandibular position towards the maxilla. Forty-one other variants were associated with PC1–5. We also identified 6 variants associated with MP and an array of common variants associated with single cephalometric parameter, although not significant after multiple corrections. And rare variants in *EP300* showed association with MP. Future studies with larger sample sizes, more candidate genes, and in other population are required to replicate these findings, and further functional studies are also warranted.

## Supplementary Information

**Additional file 1.**

**Additional file 2.**

**Additional file 3.**

**Additional file 4.**

**Additional file 5.**

**Additional file 6.**

**Additional file 7: Figure S1**. Three SNPs (rs1051415 in an exonic region of *JAG1*; rs75236173 in the 3′-UTR of *NUMB*; rs1044006 in an exonic region of *NOTCH3*) in six randomly selecting samples (including three cases and three controls) were validated by Sanger sequencing. The three cases were C02, C03 and C8, while the three control samples were A8, A42 and A43. The black arrows represent the polymorphism sites.

## Data Availability

All data generated or analysed during this study are included in this published article and its supplementary information files.
