# Supplementary material for: Targeted sequencing of NOTCH signaling pathway genes and association analysis of variants correlated with mandibular prognathism
Source: Head Face Med. 2021 May 26;17:17. doi: 10.1186/s13005-021-00268-0 (PMC8152080; doi:10.1186/s13005-021-00268-0)
Supplement: Supplementary file 1 — Additional file 1. [file 13005_2021_268_MOESM1_ESM.docx]

Table S1.Skeletal landmarks

| Landmarks | Abbreviation | Definition |
| --- | --- | --- |
| Sella | S | The midpoint of sella turcica |
| Nasion | N | Most anterior and medial point of the frontonasal suture |
| Orbitale | Or | The lowest point on the inferior rim of the orbit |
| Porion | Po | The most superiorly positioned point of the external auditory meatus |
| Basion | Ba | The midpoint of front foramen magnum |
| A point | A | The most posterior midline point in the concavity between ANS and the most inferior point on the alveolar bone overlying the maxillary incisors |
| Posterior nasal spine | PNS | The posterior spine of the palatine bone constituting the hard palate |
| Apex of upper first incisor | U1A | The apex of the upper first incisor |
| Tip of upper first incisor | U1T | The tip of the upper first incisor |
| Pterygomaxillary fissure | Ptm | The contour of the fissure projected onto the palatal plane. |
| U6 | U6 | The mesiobuccal cusp tip of the maxillary molar |
| Ao | Ao | Intersection between a perpendicular line dropped from Point A and the occlusal plane |
| AOcP | AOcP | Anterior point of occlusal plane |
| D | D | The middle point of mandibular body synostosis |
| B point | B | The most posterior midline point in the concavity of the mandible between the most superior point on the alveolar bone overlying the mandibular incisors and Pog |
| Gnathion | Gn | A point located by taking the midpoint between the anterior (pogonion) and inferior (menton) points of the bony chin |
| Anterior nasal spine  Pogonion | ANS  Pog | The anterior tip of the sharp bony process of the maxilla at the lower margin of the anterior nasal opening  The most anterior point on the chin |
| Gonion | Go | A point on the curvature of the angle of the mandible located by bisecting the angle formed by lines tangent to the posterior ramus and the inferior border of the mandible |
| Menton | Me | The lowest point on the symphyseal shadow of the mandible seen on a lateral cephalogram |
| Apex of lower first incisor | L1A | The apex of the lower first incisor |
| Tip of lower first incisor | L1T | The tip of the lower first incisor |
| Condylion | Co | Most superior point on the head of the condyle |
| L6 | L6 | The mesiobuccal cusp tip of the mandibular molar |
| Bo | Bo | Intersection between a perpendicular line dropped from Point B and the occlusal plane |
| POcP | POcP | Posterior point of occlusal plane |
| Articulare | Ar | The intersection of the posterior margin of the ascending ramus and the outer margin of the cranial base. |
| subnasale | Sn | The point at which the columella merges with the upper lip in the sagittal plane |
| lower lip | LL | The most prominent point of the vermilion border of the Cupid’s bow of the lower lip |
| Soft tissue pogonion | Pog’ | The most anterior point in the chin |
| upper lip | UL | The most prominent point of the vermilion border of the Cupid’s bow of the upper lip |
| Me’ | Me’ | The lowest point of the soft chin |
| G’  LLA  ULA | G’  LLA  ULA | The most anterior point of forehead  Lower lip anterior  Upper lip anterior |
| LLS | LLS | lower lip stomion inferius |
