# Supplementary material for: Targeted sequencing of NOTCH signaling pathway genes and association analysis of variants correlated with mandibular prognathism
Source: Head Face Med. 2021 May 26;17:17. doi: 10.1186/s13005-021-00268-0 (PMC8152080; doi:10.1186/s13005-021-00268-0)
Supplement: Supplementary file 2 — Additional file 2. [file 13005_2021_268_MOESM2_ESM.docx]

**Table-S2**. Sixty-one cephalometric parameters

| Cranial Base | Intermaxillary | Dental |
| --- | --- | --- |
| Saddle/Sella Angle(SN-Ar)(º) | ANB (º) | UI-NA (º) |
| Posterior Craneal Base Length(S-Ar) | Wits (Ao-Bo)(mm) | UI-NA(mm) |
| Anterior Craneal Base Length(SN) | FMA (FH-MP) (º) | UI-SN (º) |
|  | Y axis(N-S-Gn) (º) | LI-NB(º) |
| Maxilla | Anterior Facial Height (N-Me)(mm) | LI-NB(mm) |
| SNA (º) | Post.Fac.H/Ant.Fac.H (S-Go/N-Me)(%) | LI-MP(IMPA) (º) |
| Convexity (NA-PoA)(º) | APDI(NP-AB)(Facial Plane to AB) | Interincisal Angle (UI-LI) (º) |
| Maxilla Length(mm) | Occl. To S-N (º) | Overjet(mm) |
| N to A through Horizontal Plane | GoGn to S-N(º) | Overbite(mm) |
| Na _\|_ to A point | Difference Maxillary-Mandibular | UADH (mm)( UI-PP) |
|  | Midface Length (Co-A)(mm) | LADH (LI-MP)(mm) |
| Mandible | Occ Plane to FH (OP ^ PoOr)(º) | UPDH (U6-PP)(mm) |
| SNB(º) | Upper Face Ht (N to ans through TVL (N-ANS)(mm) | LPDH (L6-MP)(mm) |
| Pog-NB(mm)（Pg - NB） | Inferior Facial Heigh(mm) | L1 Protrusion (LI : APog)(mm) |
| MP-SN | ANS:Me % N:Me(Nasal Ht (N-ANS/N-Me) (%) | L1 Protrusion (LI ^ Apog)(º) |
| APDI(NP-FH)（Facial Angle） |  | UI ^ PoOr (U1 – FH) (º) |
| Ramus Height(Ar-Go) (mm) | **Soft Tissue** | FMIA(LI-FH) (º) |
| Posterior Facial Height (Co-Go)(mm) | Upper Lip to E-Plane (UL:En-Pog) (mm) |  |
| N to B through Horizontal Plane | Lower Lip to E-Plane (LL:En-Pog) (mm) |  |
| N to Pog through Horizontal Plane | Upper lip length (Sn - ULI) |  |
| Gonial Angle(Ar-Go-Me) (º) | Lower lip length (LLS - Me') |  |
| Mandibular Body Length(Mn Unit Length (Co-Gn) (mm) | Upper lip anterior (ULA - TVL) |  |
| Articular Angle(S-Ar-Go) (º) | Lower lip anterior (LLA - TVL) |  |
| SND(º) | Facial angle (G' - Sn - Pog') |  |
