# Supplementary material for: Targeted sequencing of NOTCH signaling pathway genes and association analysis of variants correlated with mandibular prognathism
Source: Head Face Med. 2021 May 26;17:17. doi: 10.1186/s13005-021-00268-0 (PMC8152080; doi:10.1186/s13005-021-00268-0)
Supplement: Supplementary file 3 — Additional file 3. [file 13005_2021_268_MOESM3_ESM.docx]

**Table S3.** Selected genes for studying

| **Gene** | **Chr** | **Start** | **Stop** | **Length** | **Whole length** |
| --- | --- | --- | --- | --- | --- |
| *NOTCH1* | chr9 | 139388875 | 139392030 | 3155 | 10655 |
| *NOTCH1* | chr9 | 139393330 | 139393468 | 138 |  |
| *NOTCH1* | chr9 | 139393543 | 139393731 | 188 |  |
| *NOTCH1* | chr9 | 139394983 | 139395319 | 336 |  |
| *NOTCH1* | chr9 | 139396179 | 139396385 | 206 |  |
| *NOTCH1* | chr9 | 139396432 | 139396560 | 128 |  |
| *NOTCH1* | chr9 | 139396703 | 139396960 | 257 |  |
| *NOTCH1* | chr9 | 139397613 | 139397802 | 189 |  |
| *NOTCH1* | chr9 | 139399104 | 139399576 | 472 |  |
| *NOTCH1* | chr9 | 139399741 | 139400353 | 612 |  |
| *NOTCH1* | chr9 | 139400958 | 139401111 | 153 |  |
| *NOTCH1* | chr9 | 139401147 | 139401445 | 298 |  |
| *NOTCH1* | chr9 | 139401736 | 139401909 | 173 |  |
| *NOTCH1* | chr9 | 139402386 | 139402611 | 225 |  |
| *NOTCH1* | chr9 | 139402663 | 139402857 | 194 |  |
| *NOTCH1* | chr9 | 139403301 | 139403543 | 242 |  |
| *NOTCH1* | chr9 | 139404164 | 139404433 | 269 |  |
| *NOTCH1* | chr9 | 139405084 | 139405277 | 193 |  |
| *NOTCH1* | chr9 | 139405583 | 139405743 | 160 |  |
| *NOTCH1* | chr9 | 139407452 | 139407606 | 154 |  |
| *NOTCH1* | chr9 | 139407823 | 139408009 | 186 |  |
| *NOTCH1* | chr9 | 139408941 | 139409174 | 233 |  |
| *NOTCH1* | chr9 | 139409721 | 139409872 | 151 |  |
| *NOTCH1* | chr9 | 139409914 | 139410188 | 274 |  |
| *NOTCH1* | chr9 | 139410412 | 139410566 | 154 |  |
| *NOTCH1* | chr9 | 139411703 | 139411857 | 154 |  |
| *NOTCH1* | chr9 | 139412183 | 139412409 | 226 |  |
| *NOTCH1* | chr9 | 139412568 | 139412764 | 196 |  |
| *NOTCH1* | chr9 | 139413022 | 139413296 | 274 |  |
| *NOTCH1* | chr9 | 139413874 | 139414037 | 163 |  |
| *NOTCH1* | chr9 | 139417281 | 139417660 | 379 |  |
| *NOTCH1* | chr9 | 139418148 | 139418451 | 303 |  |
| *NOTCH1* | chr9 | 139438455 | 139438574 | 119 |  |
| *NOTCH1* | chr9 | 139440157 | 139440258 | 101 |  |
| *NOTCH2* | chr1 | 120454155 | 120459337 | 5182 |  |
| *NOTCH2* | chr1 | 120460267 | 120460405 | 138 |  |
| *NOTCH2* | chr1 | 120461008 | 120461196 | 188 |  |
| *NOTCH2* | chr1 | 120461914 | 120462256 | 342 |  |
| *NOTCH2* | chr1 | 120462831 | 120463040 | 209 |  |
| *NOTCH2* | chr1 | 120464315 | 120464452 | 137 |  |
| *NOTCH2* | chr1 | 120464838 | 120465089 | 251 |  |
| *NOTCH2* | chr1 | 120465238 | 120465421 | 183 |  |
| *NOTCH2* | chr1 | 120466239 | 120466627 | 388 |  |
| *NOTCH2* | chr1 | 120467907 | 120468453 | 546 |  |
| *NOTCH2* | chr1 | 120469101 | 120469254 | 153 |  |
| *NOTCH2* | chr1 | 120471578 | 120471855 | 277 |  |
| *NOTCH2* | chr1 | 120477715 | 120478247 | 532 |  |
| *NOTCH2* | chr1 | 120478074 | 120478247 | 173 |  |
| *NOTCH2* | chr1 | 120479884 | 120480109 | 225 |  |
| *NOTCH2* | chr1 | 120480459 | 120480653 | 194 |  |
| *NOTCH2* | chr1 | 120483157 | 120483399 | 242 |  |
| *NOTCH2* | chr1 | 120484128 | 120484397 | 269 |  |
| *NOTCH2* | chr1 | 120491016 | 120491209 | 193 |  |
| *NOTCH2* | chr1 | 120491609 | 120491769 | 160 |  |
| *NOTCH2* | chr1 | 120493326 | 120493480 | 154 |  |
| *NOTCH2* | chr1 | 120496145 | 120496331 | 186 |  |
| *NOTCH2* | chr1 | 120497642 | 120497875 | 233 |  |
| *NOTCH2* | chr1 | 120501994 | 120502145 | 151 |  |
| *NOTCH2* | chr1 | 120506176 | 120506450 | 274 |  |
| *NOTCH2* | chr1 | 120508055 | 120508209 | 154 |  |
| *NOTCH2* | chr1 | 120508978 | 120509132 | 154 |  |
| *NOTCH2* | chr1 | 120510035 | 120510264 | 229 |  |
| *NOTCH2* | chr1 | 120510679 | 120510875 | 196 |  |
| *NOTCH2* | chr1 | 120512113 | 120512387 | 274 |  |
| *NOTCH2* | chr1 | 120529562 | 120529725 | 163 |  |
| *NOTCH2* | chr1 | 120539599 | 120539975 | 376 |  |
| *NOTCH2* | chr1 | 120547931 | 120548231 | 300 |  |
| *NOTCH2* | chr1 | 120572508 | 120572630 | 122 |  |
| *NOTCH2* | chr1 | 120611927 | 120612337 | 410 | 13358 |
| *NOTCH3* | chr19 | 15270423 | 15272545 | 2122 |  |
| *NOTCH3* | chr19 | 15273255 | 15273393 | 138 |  |
| *NOTCH3* | chr19 | 15276158 | 15276346 | 188 |  |
| *NOTCH3* | chr19 | 15276577 | 15276922 | 345 |  |
| *NOTCH3* | chr19 | 15278039 | 15278242 | 203 |  |
| *NOTCH3* | chr19 | 15280876 | 15281001 | 125 |  |
| *NOTCH3* | chr19 | 15281121 | 15281384 | 263 |  |
| *NOTCH3* | chr19 | 15281461 | 15281656 | 195 |  |
| *NOTCH3* | chr19 | 15284858 | 15285231 | 373 |  |
| *NOTCH3* | chr19 | 15288315 | 15288921 | 606 |  |
| *NOTCH3* | chr19 | 15289613 | 15289772 | 159 |  |
| *NOTCH3* | chr19 | 15289815 | 15290113 | 298 |  |
| *NOTCH3* | chr19 | 15290154 | 15290327 | 173 |  |
| *NOTCH3* | chr19 | 15290862 | 15291087 | 225 |  |
| *NOTCH3* | chr19 | 15291471 | 15291659 | 188 |  |
| *NOTCH3* | chr19 | 15291751 | 15291993 | 242 |  |
| *NOTCH3* | chr19 | 15292366 | 15292632 | 266 |  |
| *NOTCH3* | chr19 | 15295085 | 15295281 | 196 |  |
| *NOTCH3* | chr19 | 15295696 | 15295850 | 154 | 9391 |
| *NOTCH3* | chr19 | 15296047 | 15296239 | 192 |  |
| *NOTCH3* | chr19 | 15296277 | 15296510 | 233 |  |
| *NOTCH3* | chr19 | 15297668 | 15297819 | 151 |  |
| *NOTCH3* | chr19 | 15297895 | 15298169 | 274 |  |
| *NOTCH3* | chr19 | 15298671 | 15298825 | 154 |  |
| *NOTCH3* | chr19 | 15299025 | 15299179 | 154 |  |
| *NOTCH3* | chr19 | 15299779 | 15300005 | 226 |  |
| *NOTCH3* | chr19 | 15300063 | 15300259 | 196 |  |
| *NOTCH3* | chr19 | 15302214 | 15302488 | 274 |  |
| *NOTCH3* | chr19 | 15302535 | 15302698 | 163 |  |
| *NOTCH3* | chr19 | 15302750 | 15303129 | 379 |  |
| *NOTCH3* | chr19 | 15303167 | 15303350 | 183 |  |
| *NOTCH3* | chr19 | 15308290 | 15308409 | 119 |  |
| *NOTCH3* | chr19 | 15311578 | 15311812 | 234 |  |
| *NOTCH4* | chr6 | 32162599 | 32163947 | 1348 |  |
| *NOTCH4* | chr6 | 32164080 | 32164218 | 138 |  |
| *NOTCH4* | chr6 | 32164681 | 32164869 | 188 |  |
| *NOTCH4* | chr6 | 32165055 | 32165391 | 336 |  |
| *NOTCH4* | chr6 | 32166177 | 32166356 | 179 |  |
| *NOTCH4* | chr6 | 32166405 | 32166527 | 122 |  |
| *NOTCH4* | chr6 | 32166682 | 32166942 | 260 |  |
| *NOTCH4* | chr6 | 32168587 | 32168803 | 216 |  |
| *NOTCH4* | chr6 | 32168873 | 32169297 | 424 |  |
| *NOTCH4* | chr6 | 32169832 | 32170396 | 564 |  |
| *NOTCH4* | chr6 | 32171526 | 32171679 | 153 |  |
| *NOTCH4* | chr6 | 32171893 | 32172186 | 293 |  |
| *NOTCH4* | chr6 | 32178508 | 32178733 | 225 |  |
| *NOTCH4* | chr6 | 32180230 | 32180424 | 194 |  |
| *NOTCH4* | chr6 | 32180580 | 32180708 | 128 |  |
| *NOTCH4* | chr6 | 32180891 | 32181049 | 158 |  |
| *NOTCH4* | chr6 | 32181444 | 32181637 | 193 |  |
| *NOTCH4* | chr6 | 32181866 | 32182052 | 186 |  |
| *NOTCH4* | chr6 | 32182982 | 32183182 | 200 |  |
| *NOTCH4* | chr6 | 32184701 | 32184864 | 163 |  |
| *NOTCH4* | chr6 | 32184909 | 32185063 | 154 |  |
| *NOTCH4* | chr6 | 32185751 | 32185905 | 154 |  |
| *NOTCH4* | chr6 | 32187348 | 32187583 | 235 |  |
| *NOTCH4* | chr6 | 32187885 | 32188081 | 196 |  |
| *NOTCH4* | chr6 | 32188161 | 32188438 | 277 |  |
| *NOTCH4* | chr6 | 32188512 | 32188675 | 163 |  |
| *NOTCH4* | chr6 | 32188734 | 32189122 | 388 |  |
| *NOTCH4* | chr6 | 32190267 | 32190603 | 336 |  |
| *NOTCH4* | chr6 | 32190761 | 32190883 | 122 |  |
| *NOTCH4* | chr6 | 32191612 | 32191864 | 252 | 7945 |
| *JAG1* | chr20 | 10618311 | 10620623 | 2312 | 7027 |
| *JAG1* | chr20 | 10621410 | 10621601 | 191 |  |
| *JAG1* | chr20 | 10621740 | 10621912 | 172 |  |
| *JAG1* | chr20 | 10622087 | 10622361 | 274 |  |
| *JAG1* | chr20 | 10622410 | 10622560 | 150 |  |
| *JAG1* | chr20 | 10623115 | 10623269 | 154 |  |
| *JAG1* | chr20 | 10624405 | 10624531 | 126 |  |
| *JAG1* | chr20 | 10624984 | 10625052 | 68 |  |
| *JAG1* | chr20 | 10625490 | 10625647 | 157 |  |
| *JAG1* | chr20 | 10625770 | 10625924 | 154 |  |
| *JAG1* | chr20 | 10625983 | 10626137 | 154 |  |
| *JAG1* | chr20 | 10626598 | 10626752 | 154 |  |
| *JAG1* | chr20 | 10627566 | 10627771 | 205 |  |
| *JAG1* | chr20 | 10628587 | 10628778 | 191 |  |
| *JAG1* | chr20 | 10629176 | 10629390 | 214 |  |
| *JAG1* | chr20 | 10629688 | 10629775 | 87 |  |
| *JAG1* | chr20 | 10630149 | 10630303 | 154 |  |
| *JAG1* | chr20 | 10630874 | 10631028 | 154 |  |
| *JAG1* | chr20 | 10632208 | 10632362 | 154 |  |
| *JAG1* | chr20 | 10632758 | 10632918 | 160 |  |
| *JAG1* | chr20 | 10633095 | 10633266 | 171 |  |
| *JAG1* | chr20 | 10637025 | 10637126 | 101 |  |
| *JAG1* | chr20 | 10639095 | 10639390 | 295 |  |
| *JAG1* | chr20 | 10644590 | 10644682 | 92 |  |
| *JAG1* | chr20 | 10653328 | 10653674 | 346 |  |
| *JAG1* | chr20 | 10654077 | 10654714 | 637 |  |
| *JAG2* | chr14 | 105607297 | 105609527 | 2230 |  |
| *JAG2* | chr14 | 105609798 | 105609995 | 197 |  |
| *JAG2* | chr14 | 105611246 | 105611418 | 172 |  |
| *JAG2* | chr14 | 105612047 | 105612330 | 283 |  |
| *JAG2* | chr14 | 105612701 | 105612857 | 156 |  |
| *JAG2* | chr14 | 105612927 | 105613081 | 154 |  |
| *JAG2* | chr14 | 105613642 | 105613768 | 126 |  |
| *JAG2* | chr14 | 105613816 | 105613884 | 68 |  |
| *JAG2* | chr14 | 105614088 | 105614245 | 157 |  |
| *JAG2* | chr14 | 105614432 | 105614586 | 154 |  |
| *JAG2* | chr14 | 105614642 | 105614796 | 154 |  |
| *JAG2* | chr14 | 105615061 | 105615215 | 154 |  |
| *JAG2* | chr14 | 105615253 | 105615446 | 193 |  |
| *JAG2* | chr14 | 105615486 | 105615677 | 191 |  |
| *JAG2* | chr14 | 105616920 | 105617134 | 214 |  |
| *JAG2* | chr14 | 105617181 | 105617268 | 87 |  |
| *JAG2* | chr14 | 105617307 | 105617461 | 154 |  |
| *JAG2* | chr14 | 105617599 | 105617753 | 154 | 6875 |
| *JAG2* | chr14 | 105617942 | 105618096 | 154 |  |
| *JAG2* | chr14 | 105618254 | 105618414 | 160 |  |
| *JAG2* | chr14 | 105618477 | 105618648 | 171 |  |
| *JAG2* | chr14 | 105621878 | 105621979 | 101 |  |
| *JAG2* | chr14 | 105622054 | 105622346 | 292 |  |
| *JAG2* | chr14 | 105624022 | 105624120 | 98 |  |
| *JAG2* | chr14 | 105634073 | 105634464 | 391 |  |
| *JAG2* | chr14 | 105634671 | 105635181 | 510 |  |
| *ADAM10* | chr15 | 58887382 | 58889860 | 2478 | 5674 |
| *ADAM10* | chr15 | 58891776 | 58891943 | 167 |  |
| *ADAM10* | chr15 | 58902475 | 58902736 | 261 |  |
| *ADAM10* | chr15 | 58903177 | 58903326 | 149 |  |
| *ADAM10* | chr15 | 58903986 | 58904210 | 224 |  |
| *ADAM10* | chr15 | 58913649 | 58913840 | 191 |  |
| *ADAM10* | chr15 | 58919878 | 58920102 | 224 |  |
| *ADAM10* | chr15 | 58925374 | 58925578 | 204 |  |
| *ADAM10* | chr15 | 58932955 | 58933179 | 224 |  |
| *ADAM10* | chr15 | 58936064 | 58936197 | 133 |  |
| *ADAM10* | chr15 | 58938233 | 58938423 | 190 |  |
| *ADAM10* | chr15 | 58957275 | 58957416 | 141 |  |
| *ADAM10* | chr15 | 58971302 | 58971501 | 199 |  |
| *ADAM10* | chr15 | 58974374 | 58974533 | 159 |  |
| *ADAM10* | chr15 | 59009755 | 59009946 | 191 |  |
| *ADAM10* | chr15 | 59041658 | 59042197 | 539 |  |
| *ADAM17* | chr2 | 9629390 | 9630667 | 1277 | 4313 |
| *ADAM17* | chr2 | 9631209 | 9631300 | 91 |  |
| *ADAM17* | chr2 | 9633006 | 9633135 | 129 |  |
| *ADAM17* | chr2 | 9633855 | 9633974 | 119 |  |
| *ADAM17* | chr2 | 9634745 | 9634916 | 171 |  |
| *ADAM17* | chr2 | 9637222 | 9637397 | 175 |  |
| *ADAM17* | chr2 | 9642281 | 9642425 | 144 |  |
| *ADAM17* | chr2 | 9645274 | 9645514 | 240 |  |
| *ADAM17* | chr2 | 9650087 | 9650280 | 193 |  |
| *ADAM17* | chr2 | 9658009 | 9658138 | 129 |  |
| *ADAM17* | chr2 | 9658211 | 9658396 | 185 |  |
| *ADAM17* | chr2 | 9661311 | 9661465 | 154 |  |
| *ADAM17* | chr2 | 9663357 | 9663487 | 130 |  |
| *ADAM17* | chr2 | 9666219 | 9666393 | 174 |  |
| *ADAM17* | chr2 | 9667894 | 9668103 | 209 |  |
| *ADAM17* | chr2 | 9675942 | 9676071 | 129 |  |
| *ADAM17* | chr2 | 9676806 | 9676977 | 171 |  |
| *ADAM17* | chr2 | 9683261 | 9683434 | 173 |  |
| *ADAM17* | chr2 | 9695617 | 9695937 | 320 |  |
| *DLL1* | chr6 | 170591273 | 170591987 | 714 | 3750 |
| *DLL1* | chr6 | 170592055 | 170592213 | 158 |  |
| *DLL1* | chr6 | 170592298 | 170593137 | 839 |  |
| *DLL1* | chr6 | 170593986 | 170594243 | 257 |  |
| *DLL1* | chr6 | 170594321 | 170594531 | 210 |  |
| *DLL1* | chr6 | 170594636 | 170594807 | 171 |  |
| *DLL1* | chr6 | 170595305 | 170595406 | 101 |  |
| *DLL1* | chr6 | 170597306 | 170597604 | 298 |  |
| *DLL1* | chr6 | 170597796 | 170597897 | 101 |  |
| *DLL1* | chr6 | 170598579 | 170598916 | 337 |  |
| *DLL1* | chr6 | 170599153 | 170599717 | 564 |  |
| *DLL3* | chr19 | 39989536 | 39989703 | 167 | 3098 |
| *DLL3* | chr19 | 39989811 | 39990133 | 322 |  |
| *DLL3* | chr19 | 39991234 | 39991332 | 98 |  |
| *DLL3* | chr19 | 39993434 | 39993717 | 283 |  |
| *DLL3* | chr19 | 39994690 | 39994948 | 258 |  |
| *DLL3* | chr19 | 39995848 | 39996111 | 263 |  |
| *DLL3* | chr19 | 39997658 | 39998278 | 620 |  |
| *DLL3* | chr19 | 39998449 | 39998574 | 125 |  |
| *DLL3* | chr19 | 39998449 | 39999141 | 692 |  |
| *DLL3* | chr19 | 39998871 | 39999141 | 270 |  |
| *DLL4* | chr15 | 41221510 | 41221952 | 442 | 3860 |
| *DLL4* | chr15 | 41222024 | 41222334 | 310 |  |
| *DLL4* | chr15 | 41222802 | 41222900 | 98 |  |
| *DLL4* | chr15 | 41223680 | 41223984 | 304 |  |
| *DLL4* | chr15 | 41224348 | 41224449 | 101 |  |
| *DLL4* | chr15 | 41224494 | 41224665 | 171 |  |
| *DLL4* | chr15 | 41226725 | 41226935 | 210 |  |
| *DLL4* | chr15 | 41227075 | 41227335 | 260 |  |
| *DLL4* | chr15 | 41228405 | 41229148 | 743 |  |
| *DLL4* | chr15 | 41229595 | 41229744 | 149 |  |
| *DLL4* | chr15 | 41230206 | 41231278 | 1072 |  |
| *DTX1* | chr12 | 113495641 | 113496276 | 635 | 3648 |
| *DTX1* | chr12 | 113515208 | 113515930 | 722 |  |
| *DTX1* | chr12 | 113530946 | 113531048 | 102 |  |
| *DTX1* | chr12 | 113531323 | 113531525 | 202 |  |
| *DTX1* | chr12 | 113531822 | 113531924 | 102 |  |
| *DTX1* | chr12 | 113532573 | 113532772 | 199 |  |
| *DTX1* | chr12 | 113532826 | 113533028 | 202 |  |
| *DTX1* | chr12 | 113533109 | 113533239 | 130 |  |
| *DTX1* | chr12 | 113534499 | 113535853 | 1354 |  |
| *EP300* | chr22 | 41488593 | 41489122 | 529 |  |
| *EP300* | chr22 | 41513170 | 41513845 | 675 |  |
| *EP300* | chr22 | 41521847 | 41522064 | 217 |  |
| *EP300* | chr22 | 41523470 | 41523772 | 302 | 10001 |
| *EP300* | chr22 | 41525873 | 41526027 | 154 |  |
| *EP300* | chr22 | 41527371 | 41527657 | 286 |  |
| *EP300* | chr22 | 41531796 | 41531930 | 134 |  |
| *EP300* | chr22 | 41533636 | 41533814 | 178 |  |
| *EP300* | chr22 | 41536123 | 41536281 | 158 |  |
| *EP300* | chr22 | 41537031 | 41537246 | 215 |  |
| *EP300* | chr22 | 41542722 | 41542840 | 118 |  |
| *EP300* | chr22 | 41543820 | 41543970 | 150 |  |
| *EP300* | chr22 | 41545021 | 41545199 | 178 |  |
| *EP300* | chr22 | 41545744 | 41546222 | 478 |  |
| *EP300* | chr22 | 41547816 | 41548036 | 220 |  |
| *EP300* | chr22 | 41548189 | 41548374 | 185 |  |
| *EP300* | chr22 | 41550978 | 41551137 | 159 |  |
| *EP300* | chr22 | 41553152 | 41553432 | 280 |  |
| *EP300* | chr22 | 41554395 | 41554524 | 129 |  |
| *EP300* | chr22 | 41556625 | 41556746 | 121 |  |
| *EP300* | chr22 | 41558706 | 41558803 | 97 |  |
| *EP300* | chr22 | 41560036 | 41560154 | 118 |  |
| *EP300* | chr22 | 41562582 | 41562690 | 108 |  |
| *EP300* | chr22 | 41564432 | 41564623 | 191 |  |
| *EP300* | chr22 | 41564704 | 41564891 | 187 |  |
| *EP300* | chr22 | 41565486 | 41565640 | 154 |  |
| *EP300* | chr22 | 41566389 | 41566595 | 206 |  |
| *EP300* | chr22 | 41568482 | 41568687 | 205 |  |
| *EP300* | chr22 | 41569606 | 41569808 | 202 |  |
| *EP300* | chr22 | 41572230 | 41572552 | 322 |  |
| *EP300* | chr22 | 41572756 | 41576101 | 3345 |  |
| *HDAC1* | chr1 | 32757687 | 32757839 | 152 | 2651 |
| *HDAC1* | chr1 | 32768201 | 32768354 | 153 |  |
| *HDAC1* | chr1 | 32782245 | 32782403 | 158 |  |
| *HDAC1* | chr1 | 32790059 | 32790174 | 115 |  |
| *HDAC1* | chr1 | 32792519 | 32792698 | 179 |  |
| *HDAC1* | chr1 | 32793116 | 32793298 | 182 |  |
| *HDAC1* | chr1 | 32794648 | 32794781 | 133 |  |
| *HDAC1* | chr1 | 32796158 | 32796307 | 149 |  |
| *HDAC1* | chr1 | 32796348 | 32796529 | 181 |  |
| *HDAC1* | chr1 | 32797054 | 32797203 | 149 |  |
| *HDAC1* | chr1 | 32797256 | 32797427 | 171 |  |
| *HDAC1* | chr1 | 32797670 | 32797863 | 193 |  |
| *HDAC1* | chr1 | 32798281 | 32798370 | 89 |  |
| *HDAC1* | chr1 | 32798597 | 32799244 | 647 |  |
| *LFNG* | chr7 | 2552142 | 2552355 | 213 |  |
| *LFNG* | chr7 | 2552770 | 2552982 | 212 |  |
| *LFNG* | chr7 | 2557476 | 2557598 | 122 | 3675 |
| *LFNG* | chr7 | 2558161 | 2558252 | 91 |  |
| *LFNG* | chr7 | 2559458 | 2559947 | 489 |  |
| *LFNG* | chr7 | 2564308 | 2564397 | 89 |  |
| *LFNG* | chr7 | 2564832 | 2564972 | 140 |  |
| *LFNG* | chr7 | 2565027 | 2565221 | 194 |  |
| *LFNG* | chr7 | 2565298 | 2565424 | 126 |  |
| *LFNG* | chr7 | 2565857 | 2566063 | 206 |  |
| *LFNG* | chr7 | 2566449 | 2566575 | 126 |  |
| *LFNG* | chr7 | 2566759 | 2568083 | 1324 |  |
| *LFNG* | chr7 | 2568487 | 2568830 | 343 |  |
| *MAML1* | chr5 | 179159830 | 179160448 | 618 | 5923 |
| *MAML1* | chr5 | 179192306 | 179193762 | 1456 |  |
| *MAML1* | chr5 | 179195830 | 179196110 | 280 |  |
| *MAML1* | chr5 | 179198127 | 179198264 | 137 |  |
| *MAML1* | chr5 | 179200875 | 179204307 | 3432 |  |
| *MAML2* | chr11 | 95709736 | 95713147 | 3411 | 7311 |
| *MAML2* | chr11 | 95718674 | 95718826 | 152 |  |
| *MAML2* | chr11 | 95724663 | 95724907 | 244 |  |
| *MAML2* | chr11 | 95825035 | 95826701 | 1666 |  |
| *MAML2* | chr11 | 96074526 | 96076364 | 1838 |  |
| *MFNG* | chr22 | 37865080 | 37866087 | 1007 | 2961 |
| *MFNG* | chr22 | 37868460 | 37868586 | 126 |  |
| *MFNG* | chr22 | 37870529 | 37870735 | 206 |  |
| *MFNG* | chr22 | 37872913 | 37873039 | 126 |  |
| *MFNG* | chr22 | 37875362 | 37875556 | 194 |  |
| *MFNG* | chr22 | 37876214 | 37876357 | 143 |  |
| *MFNG* | chr22 | 37876738 | 37876827 | 89 |  |
| *MFNG* | chr22 | 37881933 | 37882445 | 512 |  |
| *MFNG* | chr22 | 37881940 | 37882498 | 558 |  |
| *NCOR2* | chr12 | 124808936 | 124810149 | 1213 |  |
| *NCOR2* | chr12 | 124810716 | 124810936 | 220 |  |
| *NCOR2* | chr12 | 124811934 | 124812199 | 265 |  |
| *NCOR2* | chr12 | 124812072 | 124812199 | 127 |  |
| *NCOR2* | chr12 | 124815370 | 124815464 | 94 |  |
| *NCOR2* | chr12 | 124816844 | 124817033 | 189 |  |
| *NCOR2* | chr12 | 124817655 | 124817845 | 190 |  |
| *NCOR2* | chr12 | 124818949 | 124819183 | 234 |  |
| *NCOR2* | chr12 | 124819660 | 124819846 | 186 |  |
| *NCOR2* | chr12 | 124820008 | 124820198 | 190 |  |
| *NCOR2* | chr12 | 124821278 | 124821746 | 468 |  |
| *NCOR2* | chr12 | 124824531 | 124824770 | 239 |  |
| *NCOR2* | chr12 | 124824819 | 124825009 | 190 |  |
| *NCOR2* | chr12 | 124825127 | 124825317 | 190 |  |
| *NCOR2* | chr12 | 124826348 | 124826640 | 292 | 11280 |
| *NCOR2* | chr12 | 124827530 | 124827792 | 262 |  |
| *NCOR2* | chr12 | 124829122 | 124829517 | 395 |  |
| *NCOR2* | chr12 | 124831089 | 124831416 | 327 |  |
| *NCOR2* | chr12 | 124832350 | 124832477 | 127 |  |
| *NCOR2* | chr12 | 124832699 | 124832880 | 181 |  |
| *NCOR2* | chr12 | 124835112 | 124835303 | 191 |  |
| *NCOR2* | chr12 | 124838618 | 124838762 | 144 |  |
| *NCOR2* | chr12 | 124839003 | 124839148 | 145 |  |
| *NCOR2* | chr12 | 124839362 | 124839505 | 143 |  |
| *NCOR2* | chr12 | 124839957 | 124840137 | 180 |  |
| *NCOR2* | chr12 | 124841167 | 124841348 | 181 |  |
| *NCOR2* | chr12 | 124841167 | 124841372 | 205 |  |
| *NCOR2* | chr12 | 124846651 | 124846863 | 212 |  |
| *NCOR2* | chr12 | 124848204 | 124848365 | 161 |  |
| *NCOR2* | chr12 | 124856547 | 124857176 | 629 |  |
| *NCOR2* | chr12 | 124858938 | 124859029 | 91 |  |
| *NCOR2* | chr12 | 124862762 | 124862950 | 188 |  |
| *NCOR2* | chr12 | 124870270 | 124870453 | 183 |  |
| *NCOR2* | chr12 | 124882644 | 124882747 | 103 |  |
| *NCOR2* | chr12 | 124885026 | 124885239 | 213 |  |
| *NCOR2* | chr12 | 124886929 | 124887127 | 198 |  |
| *NCOR2* | chr12 | 124904482 | 124904621 | 139 |  |
| *NCOR2* | chr12 | 124907026 | 124907121 | 95 |  |
| *NCOR2* | chr12 | 124911147 | 124911366 | 219 |  |
| *NCOR2* | chr12 | 124914138 | 124914269 | 131 |  |
| *NCOR2* | chr12 | 124914138 | 124914272 | 134 |  |
| *NCOR2* | chr12 | 124915140 | 124915353 | 213 |  |
| *NCOR2* | chr12 | 124922455 | 124922562 | 107 |  |
| *NCOR2* | chr12 | 124934340 | 124934433 | 93 |  |
| *NCOR2* | chr12 | 124941631 | 124941728 | 97 |  |
| *NCOR2* | chr12 | 124950698 | 124950852 | 154 |  |
| *NCOR2* | chr12 | 124957477 | 124957697 | 220 |  |
| *NCOR2* | chr12 | 124968121 | 124968339 | 218 |  |
| *NCOR2* | chr12 | 124970966 | 124971134 | 168 |  |
| *NCOR2* | chr12 | 124979672 | 124979934 | 262 |  |
| *NCOR2* | chr12 | 125020090 | 125020177 | 87 |  |
| *NCOR2* | chr12 | 125051833 | 125052030 | 197 |  |
| *NCSTN* | chr1 | 160313042 | 160313291 | 249 |  |
| *NCSTN* | chr1 | 160314245 | 160314424 | 179 |  |
| *NCSTN* | chr1 | 160314491 | 160314636 | 145 |  |
| *NCSTN* | chr1 | 160318768 | 160318932 | 164 |  |
| *NCSTN* | chr1 | 160319318 | 160319480 | 162 |  |
| *NCSTN* | chr1 | 160319874 | 160320060 | 186 |  |
| *NCSTN* | chr1 | 160320991 | 160321182 | 191 | 3794 |
| *NCSTN* | chr1 | 160321465 | 160321615 | 150 |  |
| *NCSTN* | chr1 | 160321823 | 160322016 | 193 |  |
| *NCSTN* | chr1 | 160322656 | 160322801 | 145 |  |
| *NCSTN* | chr1 | 160322929 | 160323047 | 118 |  |
| *NCSTN* | chr1 | 160323887 | 160324100 | 213 |  |
| *NCSTN* | chr1 | 160325424 | 160325567 | 143 |  |
| *NCSTN* | chr1 | 160325632 | 160325768 | 136 |  |
| *NCSTN* | chr1 | 160326029 | 160326157 | 128 |  |
| *NCSTN* | chr1 | 160326369 | 160326564 | 195 |  |
| *NCSTN* | chr1 | 160326810 | 160327063 | 253 |  |
| *NCSTN* | chr1 | 160327918 | 160328762 | 844 |  |
| *NUMB* | chr14 | 73741897 | 73744021 | 2124 | 4164 |
| *NUMB* | chr14 | 73745968 | 73746152 | 184 |  |
| *NUMB* | chr14 | 73749046 | 73749233 | 187 |  |
| *NUMB* | chr14 | 73750768 | 73751102 | 334 |  |
| *NUMB* | chr14 | 73753797 | 73754042 | 245 |  |
| *NUMB* | chr14 | 73759421 | 73759602 | 181 |  |
| *NUMB* | chr14 | 73763898 | 73764013 | 115 |  |
| *NUMB* | chr14 | 73783077 | 73783150 | 73 |  |
| *NUMB* | chr14 | 73789817 | 73789932 | 115 |  |
| *NUMB* | chr14 | 73822313 | 73822494 | 181 |  |
| *NUMB* | chr14 | 73833584 | 73833709 | 125 |  |
| *NUMB* | chr14 | 73876624 | 73876796 | 172 |  |
| *NUMB* | chr14 | 73925178 | 73925306 | 128 |  |
| *PSEN1* | chr14 | 73603122 | 73603311 | 189 | 6738 |
| *PSEN1* | chr14 | 73614482 | 73614604 | 122 |  |
| *PSEN1* | chr14 | 73614654 | 73614822 | 168 |  |
| *PSEN1* | chr14 | 73614654 | 73614834 | 180 |  |
| *PSEN1* | chr14 | 73637484 | 73637775 | 291 |  |
| *PSEN1* | chr14 | 73640253 | 73640435 | 182 |  |
| *PSEN1* | chr14 | 73653540 | 73653648 | 108 |  |
| *PSEN1* | chr14 | 73659331 | 73659592 | 261 |  |
| *PSEN1* | chr14 | 73664718 | 73664857 | 139 |  |
| *PSEN1* | chr14 | 73673073 | 73673200 | 127 |  |
| *PSEN1* | chr14 | 73678456 | 73678670 | 214 |  |
| *PSEN1* | chr14 | 73683813 | 73683972 | 159 |  |
| *PSEN1* | chr14 | 73685821 | 73690419 | 4598 |  |
| *PSEN2* | chr1 | 227058252 | 227058370 | 118 |  |
| *PSEN2* | chr1 | 227058942 | 227059125 | 183 |  |
| *PSEN2* | chr1 | 227063045 | 227063271 | 226 |  |
| *PSEN2* | chr1 | 227069568 | 227069769 | 201 |  |
| *PSEN2* | chr1 | 227071385 | 227071640 | 255 |  |
| *PSEN2* | chr1 | 227073218 | 227073400 | 182 |  |
| *PSEN2* | chr1 | 227075771 | 227075879 | 108 | 2957 |
| *PSEN2* | chr1 | 227076509 | 227076770 | 261 |  |
| *PSEN2* | chr1 | 227077715 | 227077854 | 139 |  |
| *PSEN2* | chr1 | 227078958 | 227079082 | 124 |  |
| *PSEN2* | chr1 | 227079423 | 227079565 | 142 |  |
| *PSEN2* | chr1 | 227079426 | 227079565 | 139 |  |
| *PSEN2* | chr1 | 227081687 | 227081846 | 159 |  |
| *PSEN2* | chr1 | 227083104 | 227083824 | 720 |  |
| *PSENEN* | chr19 | 36236457 | 36236621 | 164 | 1153 |
| *PSENEN* | chr19 | 36236715 | 36236912 | 197 |  |
| *PSENEN* | chr19 | 36236753 | 36236912 | 159 |  |
| *PSENEN* | chr19 | 36237299 | 36237444 | 145 |  |
| *PSENEN* | chr19 | 36237588 | 36238076 | 488 |  |
| *RBPJL* | chr20 | 43935462 | 43935604 | 142 | 4309 |
| *RBPJL* | chr20 | 43936762 | 43936911 | 149 |  |
| *RBPJL* | chr20 | 43938186 | 43938352 | 166 |  |
| *RBPJL* | chr20 | 43940208 | 43940319 | 111 |  |
| *RBPJL* | chr20 | 43940458 | 43940614 | 156 |  |
| *RBPJL* | chr20 | 43940840 | 43941055 | 215 |  |
| *RBPJL* | chr20 | 43942087 | 43942265 | 178 |  |
| *RBPJL* | chr20 | 43942654 | 43942804 | 150 |  |
| *RBPJL* | chr20 | 43943032 | 43943225 | 193 |  |
| *RBPJL* | chr20 | 43944806 | 43945002 | 196 |  |
| *RBPJL* | chr20 | 43945096 | 43945240 | 144 |  |
| *RBPJL* | chr20 | 43945096 | 43945250 | 154 |  |
| *RBPJL* | chr20 | 43945305 | 43946484 | 1179 |  |
| *RBPJL* | chr20 | 43945308 | 43946484 | 1176 |  |
| *RFNG* | chr17 | 80005757 | 80006703 | 946 | 2148 |
| *RFNG* | chr17 | 80006886 | 80007012 | 126 |  |
| *RFNG* | chr17 | 80007532 | 80007738 | 206 |  |
| *RFNG* | chr17 | 80007773 | 80007902 | 129 |  |
| *RFNG* | chr17 | 80008257 | 80008451 | 194 |  |
| *RFNG* | chr17 | 80008517 | 80008660 | 143 |  |
| *RFNG* | chr17 | 80009149 | 80009238 | 89 |  |
| *RFNG* | chr17 | 80009355 | 80009670 | 315 |  |
| *SNW1* | chr14 | 78183923 | 78184649 | 726 |  |
| *SNW1* | chr14 | 78184689 | 78184893 | 204 |  |
| *SNW1* | chr14 | 78187033 | 78187191 | 158 |  |
| *SNW1* | chr14 | 78189503 | 78189640 | 137 |  |
| *SNW1* | chr14 | 78197310 | 78197492 | 182 |  |
| *SNW1* | chr14 | 78198807 | 78198964 | 157 |  |
| *SNW1* | chr14 | 78201269 | 78201375 | 106 |  |
| *SNW1* | chr14 | 78202259 | 78202369 | 110 |  |
| *SNW1* | chr14 | 78203293 | 78203438 | 145 |  |
| *SNW1* | chr14 | 78205100 | 78205247 | 147 | 2685 |
| *SNW1* | chr14 | 78205288 | 78205424 | 136 |  |
| *SNW1* | chr14 | 78217641 | 78217843 | 202 |  |
| *SNW1* | chr14 | 78221289 | 78221483 | 194 |  |
| *SNW1* | chr14 | 78227436 | 78227517 | 81 |  |
