# Supplementary material for: Targeted sequencing of NOTCH signaling pathway genes and association analysis of variants correlated with mandibular prognathism
Source: Head Face Med. 2021 May 26;17:17. doi: 10.1186/s13005-021-00268-0 (PMC8152080; doi:10.1186/s13005-021-00268-0)
Supplement: Supplementary file 4 — Additional file 4. [file 13005_2021_268_MOESM4_ESM.docx]

**Table S4** Custom probes details.

|  | TOTAL_READS | PCT_USABLE_BASES_ON_BAIT | PCT_TARGET_BASES | MEAN_BAIT_COVERAGE | PCT_TARGET_BASES_20X | PCT_TARGET_BASES_10X | PCT_TARGET_BASES_4X |
| --- | --- | --- | --- | --- | --- | --- | --- |
| LSY12010302044 | 877089432 | 0.4182 | 0.99 | 213 | 0.98 | 0.98 | 0.99 |
| LSY2587 | 801839518 | 0.4438 | 0.99 | 206 | 0.97 | 0.98 | 0.99 |
| LSY2234 | 692964531 | 0.5045 | 0.99 | 203 | 0.98 | 0.98 | 0.99 |
| LSY12010302282 | 831185187 | 0.3931 | 0.99 | 190 | 0.97 | 0.98 | 0.99 |
| LSY5618 | 727359127 | 0.4139 | 0.99 | 175 | 0.95 | 0.97 | 0.98 |
| IS410 | 553575755 | 0.5369 | 0.99 | 172 | 0.97 | 0.98 | 0.99 |
| LSY2608 | 610528198 | 0.4695 | 0.99 | 166 | 0.97 | 0.98 | 0.99 |
| LSY5919 | 585662625 | 0.4835 | 0.99 | 164 | 0.98 | 0.98 | 0.99 |
| IS363 | 478223182 | 0.5524 | 0.99 | 153 | 0.95 | 0.97 | 0.99 |
| LSY3855 | 546311011 | 0.4658 | 0.99 | 148 | 0.97 | 0.98 | 0.99 |
| LSY12010301238 | 657275055 | 0.3772 | 0.99 | 144 | 0.97 | 0.98 | 0.99 |
| IS396 | 542955493 | 0.453 | 0.99 | 143 | 0.96 | 0.98 | 0.98 |
| IS333 | 458404019 | 0.5089 | 0.99 | 135 | 0.96 | 0.98 | 0.99 |
| LSY2763 | 536103202 | 0.4229 | 0.99 | 132 | 0.9 | 0.94 | 0.96 |
| LSY12010300700 | 561765940 | 0.4056 | 0.99 | 132 | 0.97 | 0.98 | 0.99 |
| IS391 | 405329124 | 0.5563 | 0.99 | 131 | 0.97 | 0.98 | 0.99 |
| LSY5574 | 429027860 | 0.5218 | 0.99 | 130 | 0.85 | 0.93 | 0.97 |
| LSY2184 | 463772744 | 0.4499 | 0.98 | 123 | 0.75 | 0.84 | 0.93 |
| LSY12010300539 | 555892213 | 0.381 | 0.99 | 123 | 0.96 | 0.98 | 0.99 |
| LSY12010301194 | 544451316 | 0.3896 | 0.99 | 123 | 0.97 | 0.98 | 0.99 |
| LSY12010302211 | 486790774 | 0.4339 | 0.99 | 123 | 0.97 | 0.98 | 0.99 |
| IS474 | 402276505 | 0.5242 | 0.99 | 122 | 0.96 | 0.98 | 0.99 |
| LSY3790 | 466044547 | 0.4465 | 0.98 | 122 | 0.9 | 0.94 | 0.96 |
| LSY2583 | 442803840 | 0.4676 | 0.99 | 120 | 0.97 | 0.98 | 0.99 |
| IS327 | 358818193 | 0.5466 | 0.99 | 114 | 0.96 | 0.98 | 0.99 |
| IS382 | 349798519 | 0.5623 | 0.99 | 114 | 0.96 | 0.98 | 0.99 |
| IS392 | 408388108 | 0.475 | 0.99 | 113 | 0.96 | 0.98 | 0.98 |
| IS359 | 365028901 | 0.5298 | 0.99 | 112 | 0.96 | 0.98 | 0.99 |
| LSY3442 | 403677152 | 0.4806 | 0.99 | 112 | 0.96 | 0.98 | 0.98 |
| LSY12010302393 | 556881277 | 0.3458 | 0.99 | 112 | 0.95 | 0.97 | 0.98 |
| LSY2938 | 450485373 | 0.4239 | 0.99 | 111 | 0.95 | 0.97 | 0.98 |
| LSY4455 | 414850407 | 0.4613 | 0.99 | 111 | 0.96 | 0.97 | 0.98 |
| IS498 | 352948214 | 0.527 | 0.99 | 108 | 0.95 | 0.97 | 0.98 |
| LSY12010302642 | 618787142 | 0.3006 | 0.99 | 108 | 0.97 | 0.98 | 0.99 |
| LSY0994 | 453938724 | 0.403 | 0.98 | 107 | 0.69 | 0.81 | 0.93 |
| IS246 | 378448132 | 0.4797 | 0.99 | 105 | 0.94 | 0.98 | 0.99 |
| LSY2254 | 446194801 | 0.4012 | 0.98 | 105 | 0.84 | 0.89 | 0.94 |
| LSY12010301032 | 447515527 | 0.4069 | 0.99 | 105 | 0.97 | 0.98 | 0.99 |
| LSY5512 | 437511503 | 0.4096 | 0.99 | 104 | 0.92 | 0.95 | 0.97 |
|  |  |  |  |  |  |  |  |
| LSY3658 | 394765204 | 0.4508 | 0.99 | 103 | 0.97 | 0.98 | 0.98 |
| LSY5154 | 441799057 | 0.4003 | 0.99 | 103 | 0.95 | 0.97 | 0.98 |
| LSY12010301076 | 440121383 | 0.4057 | 0.99 | 103 | 0.96 | 0.98 | 0.99 |
| IS375 | 382541092 | 0.4605 | 0.99 | 102 | 0.95 | 0.97 | 0.98 |
| LSY12010300637 | 382609365 | 0.4615 | 0.99 | 102 | 0.96 | 0.98 | 0.98 |
| LSY12010301409 | 457758422 | 0.3835 | 0.99 | 102 | 0.96 | 0.98 | 0.99 |
| IS409 | 408540475 | 0.4247 | 0.99 | 101 | 0.95 | 0.97 | 0.98 |
| LSY4320 | 379514395 | 0.4581 | 0.99 | 101 | 0.89 | 0.93 | 0.96 |
| LSY5149 | 417789888 | 0.4123 | 0.98 | 101 | 0.86 | 0.91 | 0.95 |
| LSY3660 | 421152234 | 0.4049 | 0.98 | 100 | 0.9 | 0.94 | 0.96 |
| LSY4690 | 421079271 | 0.4095 | 0.99 | 100 | 0.95 | 0.97 | 0.98 |
| LSY2103 | 433996971 | 0.3899 | 0.99 | 99 | 0.91 | 0.94 | 0.97 |
| IS448 | 358159544 | 0.4698 | 0.99 | 98 | 0.95 | 0.97 | 0.98 |
| IS486 | 357078278 | 0.4742 | 0.99 | 98 | 0.94 | 0.97 | 0.98 |
| LSY4582 | 335306324 | 0.4952 | 0.99 | 97 | 0.91 | 0.94 | 0.97 |
| LSY5463 | 372014455 | 0.4486 | 0.99 | 97 | 0.93 | 0.96 | 0.98 |
| LSY12010300638 | 433272682 | 0.3822 | 0.99 | 96 | 0.96 | 0.98 | 0.99 |
| LSY12010300698 | 285120650 | 0.5747 | 0.99 | 95 | 0.96 | 0.98 | 0.98 |
| LSY12010300940 | 420351440 | 0.387 | 0.99 | 94 | 0.96 | 0.97 | 0.98 |
| IS439 | 338656274 | 0.4728 | 0.99 | 93 | 0.93 | 0.97 | 0.98 |
| LSY3394 | 440605855 | 0.3647 | 0.99 | 93 | 0.96 | 0.98 | 0.99 |
| LSY12010300639 | 358854860 | 0.4469 | 0.99 | 93 | 0.96 | 0.98 | 0.98 |
| S4 | 414902211 | 0.375 | 0.99 | 91 | 0.92 | 0.95 | 0.97 |
| IS384 | 330031258 | 0.4759 | 0.99 | 91 | 0.94 | 0.97 | 0.98 |
| LSY4430 | 353971456 | 0.4378 | 0.99 | 90 | 0.93 | 0.96 | 0.98 |
| IS244 | 317110365 | 0.4839 | 0.99 | 89 | 0.95 | 0.97 | 0.99 |
| IS487 | 379851199 | 0.4041 | 0.99 | 89 | 0.94 | 0.97 | 0.98 |
| LSY12010301729 | 358093075 | 0.429 | 0.99 | 89 | 0.96 | 0.98 | 0.99 |
| S2 | 385984561 | 0.3903 | 0.99 | 88 | 0.9 | 0.95 | 0.97 |
| IS414 | 297113178 | 0.5101 | 0.99 | 88 | 0.96 | 0.98 | 0.98 |
| IS445 | 343931867 | 0.4395 | 0.99 | 88 | 0.93 | 0.97 | 0.98 |
| IS170 | 280831939 | 0.5325 | 0.99 | 87 | 0.93 | 0.97 | 0.99 |
| LSY12010301212 | 361735537 | 0.4167 | 0.99 | 87 | 0.96 | 0.97 | 0.98 |
| LSY12010301623 | 360043386 | 0.4165 | 0.99 | 87 | 0.95 | 0.97 | 0.98 |
| LSY2459 | 317020440 | 0.4663 | 0.99 | 86 | 0.95 | 0.97 | 0.98 |
| LSY3552 | 356535032 | 0.4142 | 0.98 | 86 | 0.76 | 0.84 | 0.92 |
| 1S112 | 255526047 | 0.5726 | 0.99 | 85 | 0.95 | 0.97 | 0.98 |
| S15 | 399660399 | 0.3646 | 0.99 | 85 | 0.9 | 0.95 | 0.97 |
| IS148 | 306921830 | 0.4751 | 0.99 | 84 | 0.92 | 0.97 | 0.98 |
| IS499 | 289536309 | 0.5009 | 0.99 | 84 | 0.95 | 0.98 | 0.99 |
| LSY4391 | 309899870 | 0.4663 | 0.99 | 84 | 0.83 | 0.89 | 0.95 |
| LSY5168 | 350692088 | 0.4128 | 0.99 | 84 | 0.95 | 0.97 | 0.98 |
| LSY12010301031 | 334625773 | 0.4328 | 0.99 | 84 | 0.95 | 0.97 | 0.98 |
| LSY12010301275 | 353711317 | 0.4098 | 0.99 | 84 | 0.95 | 0.97 | 0.98 |
| LSY12010302143 | 313528028 | 0.4626 | 0.99 | 84 | 0.95 | 0.97 | 0.98 |
| IS370 | 289462727 | 0.4943 | 0.99 | 83 | 0.94 | 0.97 | 0.99 |
| LSY12010302246 | 362957326 | 0.3924 | 0.99 | 83 | 0.94 | 0.97 | 0.98 |
| LSY12030105389 | 423850802 | 0.3378 | 0.99 | 83 | 0.96 | 0.98 | 0.99 |
| LSY4138 | 319799174 | 0.4427 | 0.99 | 82 | 0.96 | 0.98 | 0.98 |
| IS331 | 312335497 | 0.4462 | 0.99 | 81 | 0.93 | 0.97 | 0.98 |
| IS401 | 347035216 | 0.4046 | 0.99 | 81 | 0.94 | 0.97 | 0.98 |
| LSY2849 | 289650474 | 0.4854 | 0.99 | 81 | 0.96 | 0.98 | 0.98 |
| LSY3845 | 355034725 | 0.3944 | 0.99 | 81 | 0.95 | 0.97 | 0.98 |
| LSY12010301587 | 338364234 | 0.4141 | 0.99 | 81 | 0.94 | 0.97 | 0.98 |
| LSY12010302199 | 322408323 | 0.4334 | 0.99 | 81 | 0.94 | 0.97 | 0.98 |
| S3 | 403220060 | 0.3419 | 0.98 | 80 | 0.9 | 0.95 | 0.97 |
| IS077 | 246255414 | 0.5627 | 0.99 | 80 | 0.94 | 0.97 | 0.98 |
| LSY12010302122 | 350785547 | 0.3944 | 0.99 | 80 | 0.95 | 0.97 | 0.98 |
| LSY12030105668 | 488463260 | 0.2823 | 0.99 | 80 | 0.97 | 0.98 | 0.99 |
| LSY12010301187 | 310423028 | 0.4382 | 0.99 | 79 | 0.95 | 0.97 | 0.98 |
| IS151 | 275610322 | 0.4863 | 0.99 | 78 | 0.91 | 0.96 | 0.98 |
| LSY3675 | 312592256 | 0.4283 | 0.99 | 78 | 0.92 | 0.96 | 0.98 |
| LSY3677 | 322909902 | 0.4149 | 0.99 | 78 | 0.95 | 0.97 | 0.98 |
| LSY12010300695 | 324212502 | 0.4159 | 0.99 | 78 | 0.95 | 0.97 | 0.98 |
| LSY12010300702 | 342934165 | 0.3849 | 0.99 | 77 | 0.95 | 0.97 | 0.98 |
| LSY1217 | 353789630 | 0.3704 | 0.99 | 76 | 0.95 | 0.98 | 0.99 |
| LSY12010301307 | 311007835 | 0.4208 | 0.99 | 76 | 0.94 | 0.97 | 0.98 |
| S29 | 351207174 | 0.3677 | 0.98 | 75 | 0.86 | 0.92 | 0.96 |
| IS367 | 255950671 | 0.5059 | 0.99 | 75 | 0.91 | 0.96 | 0.98 |
| LSY1126 | 316033847 | 0.4023 | 0.99 | 74 | 0.89 | 0.94 | 0.97 |
| LSY12010301465 | 313672881 | 0.407 | 0.99 | 74 | 0.94 | 0.97 | 0.98 |
| LSY12010301557 | 300473004 | 0.4272 | 0.99 | 74 | 0.95 | 0.97 | 0.98 |
| TXRY12E0314 | 283942358 | 0.4375 | 0.97 | 73 | 0.76 | 0.84 | 0.92 |
| S50 | 381084640 | 0.3278 | 0.98 | 73 | 0.83 | 0.91 | 0.96 |
| IS126 | 253107036 | 0.4991 | 0.99 | 73 | 0.92 | 0.97 | 0.98 |
| IS400 | 275523300 | 0.4537 | 0.99 | 73 | 0.93 | 0.96 | 0.98 |
| LSY12010202355 | 268504250 | 0.4718 | 0.99 | 73 | 0.85 | 0.93 | 0.97 |
| LSY12010301575 | 287291280 | 0.4414 | 0.99 | 73 | 0.95 | 0.97 | 0.99 |
| LSY12010301671 | 296141248 | 0.4259 | 0.99 | 73 | 0.94 | 0.97 | 0.98 |
| LSY12010302678 | 410144896 | 0.3054 | 0.99 | 73 | 0.91 | 0.95 | 0.98 |
| S30 | 332817463 | 0.3733 | 0.99 | 72 | 0.88 | 0.94 | 0.97 |
| IS198 | 280364912 | 0.4454 | 0.99 | 72 | 0.92 | 0.96 | 0.98 |
| LSY4894 | 239230209 | 0.502 | 0.95 | 72 | 0.75 | 0.81 | 0.88 |
| LSY5225 | 277376159 | 0.4441 | 0.99 | 72 | 0.9 | 0.94 | 0.97 |
| LSY12010302613 | 403360830 | 0.309 | 0.99 | 72 | 0.95 | 0.97 | 0.98 |
| S34 | 351576674 | 0.3473 | 0.98 | 71 | 0.86 | 0.92 | 0.96 |
| LSY1894 | 323488297 | 0.3705 | 0.97 | 71 | 0.73 | 0.81 | 0.9 |
| LSY4987 | 305394500 | 0.3923 | 0.97 | 71 | 0.68 | 0.77 | 0.9 |
| IS040 | 381944499 | 0.3166 | 0.99 | 70 | 0.94 | 0.97 | 0.99 |
| IS134 | 235307346 | 0.5128 | 0.99 | 70 | 0.9 | 0.96 | 0.98 |
| IS205 | 262054046 | 0.4591 | 0.99 | 70 | 0.93 | 0.97 | 0.98 |
| IS235 | 339310382 | 0.3582 | 0.99 | 70 | 0.95 | 0.97 | 0.98 |
| IS329 | 224934598 | 0.5386 | 0.99 | 70 | 0.94 | 0.97 | 0.98 |
| IS436 | 249138499 | 0.4869 | 0.99 | 70 | 0.94 | 0.97 | 0.98 |
|  |  |  |  |  |  |  |  |
| LSY5476 | 338930855 | 0.351 | 0.99 | 69 | 0.95 | 0.97 | 0.98 |
| LSY5583 | 313166200 | 0.3649 | 0.95 | 69 | 0.78 | 0.84 | 0.9 |
| LSY12010301049 | 274948335 | 0.4355 | 0.99 | 69 | 0.94 | 0.97 | 0.98 |
| LSY12010302371 | 359982320 | 0.3285 | 0.99 | 69 | 0.94 | 0.97 | 0.98 |
| LSY12010302495 | 374185419 | 0.3165 | 0.99 | 69 | 0.93 | 0.96 | 0.98 |
| S10 | 312692521 | 0.3745 | 0.98 | 68 | 0.87 | 0.93 | 0.97 |
| S13 | 353997044 | 0.33 | 0.98 | 68 | 0.79 | 0.89 | 0.96 |
| IS402 | 253068804 | 0.4663 | 0.99 | 68 | 0.94 | 0.97 | 0.98 |
| LSY11010202184 | 235753462 | 0.4986 | 0.99 | 68 | 0.88 | 0.95 | 0.98 |
| LSY12030104946 | 337197006 | 0.3485 | 0.99 | 68 | 0.96 | 0.98 | 0.99 |
| S1 | 324204426 | 0.3569 | 0.99 | 67 | 0.87 | 0.94 | 0.97 |
| IS263 | 264148434 | 0.4395 | 0.99 | 67 | 0.92 | 0.97 | 0.98 |
| IS330 | 242095888 | 0.4782 | 0.99 | 67 | 0.91 | 0.96 | 0.98 |
| IS493 | 276694010 | 0.4164 | 0.99 | 67 | 0.92 | 0.96 | 0.98 |
| LSY4036 | 270436169 | 0.4241 | 0.99 | 67 | 0.94 | 0.97 | 0.98 |
| LSY4226 | 284271415 | 0.4039 | 0.99 | 67 | 0.95 | 0.97 | 0.98 |
| LSY4360 | 279004844 | 0.4113 | 0.99 | 67 | 0.89 | 0.94 | 0.97 |
| LSY12010300831 | 294791132 | 0.3951 | 0.99 | 67 | 0.95 | 0.97 | 0.98 |
| LSY12010301539 | 288457806 | 0.4026 | 0.99 | 67 | 0.94 | 0.97 | 0.98 |
| LSY12010301635 | 269331864 | 0.4313 | 0.99 | 67 | 0.93 | 0.97 | 0.98 |
| LSY12010301678 | 247356994 | 0.4684 | 0.99 | 67 | 0.94 | 0.97 | 0.98 |
| IS291 | 237282503 | 0.4799 | 0.99 | 66 | 0.91 | 0.96 | 0.98 |
| IS406 | 268486795 | 0.4265 | 0.99 | 66 | 0.93 | 0.96 | 0.98 |
| LSY12010300716 | 279621548 | 0.4103 | 0.99 | 66 | 0.93 | 0.97 | 0.98 |
| LSY12010301047 | 277274651 | 0.412 | 0.99 | 66 | 0.94 | 0.97 | 0.98 |
| LSY12010301296 | 291077555 | 0.391 | 0.99 | 66 | 0.94 | 0.97 | 0.98 |
| IS083 | 226627120 | 0.492 | 0.99 | 65 | 0.91 | 0.96 | 0.98 |
| IS362 | 245931669 | 0.4543 | 0.99 | 65 | 0.89 | 0.95 | 0.98 |
| IS437 | 275395045 | 0.4078 | 0.99 | 65 | 0.92 | 0.96 | 0.98 |
| IS440 | 267300374 | 0.4188 | 0.99 | 65 | 0.91 | 0.96 | 0.98 |
| LSY3149 | 243665573 | 0.4638 | 0.99 | 65 | 0.95 | 0.97 | 0.98 |
| LSY12010202255 | 255315055 | 0.441 | 0.99 | 65 | 0.87 | 0.95 | 0.98 |
| S9 | 395104668 | 0.2805 | 0.99 | 64 | 0.87 | 0.94 | 0.97 |
| S38 | 410638831 | 0.2678 | 0.99 | 64 | 0.9 | 0.94 | 0.97 |
| IS195 | 241417458 | 0.4586 | 0.99 | 64 | 0.85 | 0.94 | 0.98 |
| IS290 | 289188862 | 0.384 | 0.99 | 64 | 0.95 | 0.97 | 0.98 |
| IS398 | 224583662 | 0.4923 | 0.99 | 64 | 0.93 | 0.96 | 0.98 |
| LSY2736 | 261264927 | 0.4185 | 0.99 | 64 | 0.88 | 0.93 | 0.97 |
| LSY3873 | 249796808 | 0.4438 | 0.99 | 64 | 0.95 | 0.97 | 0.98 |
| LSY12010301528 | 285607897 | 0.3898 | 0.99 | 64 | 0.93 | 0.97 | 0.98 |
| S5 | 374857694 | 0.2903 | 0.98 | 63 | 0.9 | 0.95 | 0.97 |
| IS076 | 207386565 | 0.5216 | 0.99 | 63 | 0.92 | 0.96 | 0.98 |
| IS441 | 226521853 | 0.4844 | 0.99 | 63 | 0.92 | 0.96 | 0.98 |
| LSY12010302249 | 290714086 | 0.3723 | 0.99 | 63 | 0.91 | 0.95 | 0.98 |
| LSY12030106285 | 332385145 | 0.3296 | 0.99 | 63 | 0.94 | 0.97 | 0.98 |
| IS090 | 231681576 | 0.4609 | 0.99 | 62 | 0.91 | 0.96 | 0.98 |
| IS347 | 254911515 | 0.4223 | 0.99 | 62 | 0.93 | 0.97 | 0.98 |
| LSY1759 | 286590247 | 0.3672 | 0.97 | 62 | 0.67 | 0.76 | 0.89 |
| LSY5483 | 248827161 | 0.4251 | 0.98 | 62 | 0.85 | 0.91 | 0.95 |
| LSY12010302628 | 472044541 | 0.2267 | 0.99 | 62 | 0.95 | 0.97 | 0.99 |
| LSY12030105793 | 227499042 | 0.4726 | 0.99 | 62 | 0.93 | 0.97 | 0.98 |
| 1S138 | 249034204 | 0.4215 | 0.99 | 61 | 0.88 | 0.95 | 0.98 |
| IS245 | 221781144 | 0.4788 | 0.99 | 61 | 0.93 | 0.97 | 0.98 |
| IS353 | 222938158 | 0.4713 | 0.99 | 61 | 0.92 | 0.96 | 0.98 |
| LSY1909 | 254140269 | 0.416 | 0.99 | 61 | 0.96 | 0.97 | 0.98 |
| LSY12010300548 | 238228750 | 0.4441 | 0.99 | 61 | 0.91 | 0.97 | 0.98 |
| LSY12010301981 | 280848077 | 0.3725 | 0.99 | 61 | 0.93 | 0.97 | 0.98 |
| LSY12030104829 | 229282356 | 0.4613 | 0.99 | 61 | 0.92 | 0.96 | 0.98 |
| S7 | 361907352 | 0.2847 | 0.99 | 60 | 0.83 | 0.92 | 0.97 |
| IS080 | 217318625 | 0.4753 | 0.99 | 60 | 0.9 | 0.96 | 0.98 |
| LSY2588 | 262888316 | 0.3863 | 0.97 | 60 | 0.84 | 0.89 | 0.93 |
| LSY3731 | 209556976 | 0.4946 | 0.99 | 60 | 0.93 | 0.97 | 0.98 |
| LSY5210 | 268977973 | 0.3802 | 0.97 | 60 | 0.62 | 0.73 | 0.88 |
| TXRY12E0297 | 251049634 | 0.4082 | 0.99 | 59 | 0.79 | 0.89 | 0.95 |
| IS358 | 242808564 | 0.4177 | 0.99 | 59 | 0.91 | 0.96 | 0.98 |
| IS456 | 209186601 | 0.487 | 0.99 | 59 | 0.94 | 0.97 | 0.98 |
| S23 | 316620497 | 0.3165 | 0.99 | 58 | 0.88 | 0.94 | 0.97 |
| IS055 | 240663484 | 0.4157 | 0.99 | 58 | 0.9 | 0.96 | 0.98 |
| LSY5536 | 366328260 | 0.2711 | 0.98 | 58 | 0.77 | 0.84 | 0.92 |
| IS344 | 213746647 | 0.4631 | 0.99 | 57 | 0.92 | 0.96 | 0.98 |
| IS399 | 212574305 | 0.4601 | 0.98 | 57 | 0.9 | 0.94 | 0.97 |
| IS495 | 223058989 | 0.4415 | 0.99 | 57 | 0.93 | 0.96 | 0.98 |
| LSY5528 | 218627426 | 0.4463 | 0.99 | 57 | 0.9 | 0.95 | 0.97 |
| S33 | 314673072 | 0.3065 | 0.98 | 56 | 0.86 | 0.93 | 0.96 |
| IS326 | 212566780 | 0.4589 | 0.99 | 56 | 0.93 | 0.97 | 0.98 |
| IS389 | 237154291 | 0.4074 | 0.99 | 56 | 0.92 | 0.96 | 0.98 |
| LSY3459 | 249011262 | 0.3872 | 0.99 | 56 | 0.91 | 0.95 | 0.98 |
| LSY4034 | 277226299 | 0.3461 | 0.99 | 56 | 0.9 | 0.95 | 0.97 |
| LSY12010301943 | 217489067 | 0.4469 | 0.99 | 56 | 0.92 | 0.96 | 0.98 |
| IS039 | 198528052 | 0.4771 | 0.99 | 55 | 0.87 | 0.95 | 0.98 |
| IS169 | 232454453 | 0.4066 | 0.99 | 55 | 0.96 | 0.98 | 0.99 |
| IS242 | 233842623 | 0.403 | 0.99 | 55 | 0.91 | 0.96 | 0.98 |
| IS265 | 225813170 | 0.4213 | 0.99 | 55 | 0.91 | 0.96 | 0.98 |
| IS395 | 198662648 | 0.4812 | 0.99 | 55 | 0.92 | 0.96 | 0.98 |
| LSY12010300703 | 220965409 | 0.4308 | 0.99 | 55 | 0.93 | 0.97 | 0.98 |
| LSY12010301599 | 211346419 | 0.4508 | 0.99 | 55 | 0.91 | 0.96 | 0.98 |
| LSY12030105490 | 280914209 | 0.3404 | 0.99 | 55 | 0.94 | 0.97 | 0.98 |
| LSY5558 | 214402354 | 0.4328 | 0.99 | 54 | 0.91 | 0.95 | 0.97 |
| LSY5639 | 225610595 | 0.413 | 0.99 | 54 | 0.93 | 0.97 | 0.98 |
| LSY12010300701 | 234586242 | 0.3954 | 0.99 | 54 | 0.92 | 0.96 | 0.98 |
| LSY12010301808 | 232324553 | 0.4028 | 0.99 | 54 | 0.91 | 0.96 | 0.98 |
| IS413 | 205538382 | 0.4439 | 0.99 | 53 | 0.94 | 0.97 | 0.98 |
| LSY2049 | 246963772 | 0.3631 | 0.97 | 53 | 0.63 | 0.74 | 0.89 |
| LSY12030104251 | 255155327 | 0.3561 | 0.99 | 53 | 0.92 | 0.96 | 0.98 |
| IS215 | 205568251 | 0.4381 | 0.99 | 52 | 0.89 | 0.96 | 0.98 |
| IS303 | 275294674 | 0.3267 | 0.99 | 52 | 0.95 | 0.97 | 0.98 |
| IS322 | 224877159 | 0.3977 | 0.99 | 52 | 0.95 | 0.97 | 0.98 |
| LSY4224 | 214191120 | 0.4196 | 0.98 | 52 | 0.87 | 0.93 | 0.97 |
| LSY4621 | 226828966 | 0.3923 | 0.98 | 52 | 0.66 | 0.77 | 0.9 |
| LSY5497 | 196976800 | 0.4549 | 0.98 | 52 | 0.85 | 0.92 | 0.96 |
| LSY1576 | 211160552 | 0.4156 | 0.98 | 51 | 0.86 | 0.92 | 0.96 |
| LSY3426 | 209934742 | 0.4216 | 0.99 | 51 | 0.93 | 0.97 | 0.98 |
| LSY4511 | 220977459 | 0.3887 | 0.97 | 51 | 0.69 | 0.78 | 0.89 |
| LSY12010202466 | 222020567 | 0.402 | 0.99 | 51 | 0.82 | 0.92 | 0.97 |
| S43 | 260945516 | 0.3265 | 0.98 | 50 | 0.77 | 0.88 | 0.95 |
| IS447 | 202176298 | 0.4249 | 0.99 | 50 | 0.89 | 0.95 | 0.97 |
| IS460 | 185666272 | 0.4672 | 0.99 | 50 | 0.91 | 0.96 | 0.98 |
| LSY5659 | 262444171 | 0.3244 | 0.97 | 50 | 0.59 | 0.68 | 0.85 |
| LSY12010202294 | 195502988 | 0.4463 | 0.99 | 50 | 0.79 | 0.91 | 0.97 |
| LSY12010301367 | 199933596 | 0.4357 | 0.99 | 50 | 0.89 | 0.96 | 0.98 |
| LSY12010302061 | 176218160 | 0.4888 | 0.99 | 50 | 0.92 | 0.96 | 0.98 |
| IS250 | 209829436 | 0.4074 | 0.99 | 49 | 0.89 | 0.96 | 0.98 |
| LSY2052 | 190410395 | 0.4481 | 0.99 | 49 | 0.93 | 0.97 | 0.98 |
| LSY2832 | 194727909 | 0.4312 | 0.99 | 49 | 0.89 | 0.95 | 0.98 |
| LSY12010202238 | 211556518 | 0.4027 | 0.99 | 49 | 0.86 | 0.94 | 0.98 |
| LSY12030104516 | 166720160 | 0.5084 | 0.99 | 49 | 0.91 | 0.96 | 0.98 |
| LSY12030105386 | 352761108 | 0.2391 | 0.99 | 49 | 0.95 | 0.97 | 0.98 |
| LSY12030105483 | 198022872 | 0.4291 | 0.99 | 49 | 0.9 | 0.96 | 0.98 |
| LSY12030105637 | 221301342 | 0.3847 | 0.99 | 49 | 0.93 | 0.97 | 0.98 |
| S14 | 309807553 | 0.2684 | 0.99 | 48 | 0.86 | 0.94 | 0.97 |
| IS016 | 178486952 | 0.4651 | 0.99 | 48 | 0.91 | 0.96 | 0.98 |
| LSY5094 | 205107466 | 0.3991 | 0.97 | 48 | 0.58 | 0.7 | 0.88 |
| LSY11010202198 | 171579331 | 0.4884 | 0.99 | 48 | 0.85 | 0.94 | 0.98 |
| LSY12010301302 | 208355791 | 0.4013 | 0.99 | 48 | 0.91 | 0.96 | 0.98 |
| LSY12010302460 | 370166871 | 0.2269 | 0.99 | 48 | 0.93 | 0.97 | 0.99 |
| LSY12030104309 | 206108886 | 0.4063 | 0.99 | 48 | 0.88 | 0.95 | 0.97 |
| IS199 | 182961716 | 0.446 | 0.99 | 47 | 0.89 | 0.95 | 0.98 |
| IS366 | 192477027 | 0.4187 | 0.99 | 47 | 0.86 | 0.94 | 0.97 |
| IS390 | 183259720 | 0.4408 | 0.99 | 47 | 0.9 | 0.95 | 0.98 |
| LSY2036 | 252854218 | 0.317 | 0.97 | 47 | 0.64 | 0.74 | 0.88 |
| LSY4402 | 164835235 | 0.4914 | 0.98 | 47 | 0.82 | 0.89 | 0.94 |
| LSY5415 | 184593821 | 0.4402 | 0.98 | 47 | 0.81 | 0.89 | 0.94 |
| S64 | 353082259 | 0.2258 | 0.98 | 46 | 0.84 | 0.93 | 0.96 |
| LSY1173 | 189698923 | 0.4179 | 0.98 | 46 | 0.85 | 0.92 | 0.95 |
| LSY4417 | 208816334 | 0.377 | 0.97 | 46 | 0.74 | 0.83 | 0.91 |
| LSY12010300493 | 159767078 | 0.4997 | 0.99 | 46 | 0.9 | 0.96 | 0.98 |
| LSY12010301413 | 235698186 | 0.3377 | 0.99 | 46 | 0.93 | 0.97 | 0.98 |
| LSY12010302276 | 177537911 | 0.4453 | 0.99 | 46 | 0.9 | 0.96 | 0.98 |
| S48 | 287202411 | 0.2686 | 0.98 | 45 | 0.8 | 0.91 | 0.96 |
| IS064 | 181779934 | 0.4288 | 0.99 | 45 | 0.86 | 0.95 | 0.98 |
| LSY1388 | 193540671 | 0.4052 | 0.99 | 45 | 0.89 | 0.96 | 0.98 |
| LSY5405 | 201723678 | 0.3835 | 0.97 | 45 | 0.6 | 0.71 | 0.87 |
| LSY12010202491 | 198803680 | 0.3928 | 0.99 | 45 | 0.82 | 0.93 | 0.98 |
| LSY12010301526 | 199970198 | 0.3899 | 0.99 | 45 | 0.89 | 0.95 | 0.98 |
| S37 | 211544414 | 0.3589 | 0.98 | 44 | 0.79 | 0.89 | 0.95 |
| IS059 | 193079319 | 0.3976 | 0.99 | 44 | 0.85 | 0.94 | 0.98 |
| S81 | 314683775 | 0.2391 | 0.98 | 44 | 0.85 | 0.93 | 0.96 |
| IS444 | 158118422 | 0.4782 | 0.99 | 44 | 0.9 | 0.95 | 0.97 |
| LSY12010202385 | 163987608 | 0.4717 | 0.99 | 44 | 0.84 | 0.94 | 0.98 |
| LSY12030104283 | 179170203 | 0.4266 | 0.99 | 44 | 0.85 | 0.93 | 0.97 |
| TXRY12E0130 | 185005513 | 0.4022 | 0.98 | 43 | 0.77 | 0.86 | 0.93 |
| IS030 | 179510764 | 0.4144 | 0.99 | 43 | 0.87 | 0.95 | 0.98 |
| S63 | 360514583 | 0.2047 | 0.98 | 43 | 0.85 | 0.93 | 0.96 |
| IS432 | 174301111 | 0.4238 | 0.99 | 43 | 0.85 | 0.94 | 0.97 |
| LSY4930 | 187608337 | 0.3985 | 0.99 | 43 | 0.86 | 0.93 | 0.97 |
| LSY5383 | 163972927 | 0.4466 | 0.98 | 43 | 0.77 | 0.87 | 0.93 |
| LSY5575 | 212835690 | 0.3466 | 0.98 | 43 | 0.64 | 0.76 | 0.91 |
| IS067 | 171465064 | 0.4257 | 0.99 | 42 | 0.86 | 0.95 | 0.98 |
| IS167 | 215798711 | 0.3383 | 0.99 | 42 | 0.91 | 0.96 | 0.98 |
| IS175 | 258386458 | 0.2826 | 0.99 | 42 | 0.92 | 0.97 | 0.98 |
| IS293 | 181825475 | 0.3978 | 0.99 | 42 | 0.92 | 0.97 | 0.98 |
| LSY12010202488 | 173377178 | 0.4177 | 0.99 | 42 | 0.79 | 0.92 | 0.97 |
| LSY12010202597 | 166938268 | 0.4402 | 0.99 | 42 | 0.81 | 0.93 | 0.98 |
| LSY12010300692 | 170999533 | 0.425 | 0.99 | 42 | 0.86 | 0.95 | 0.98 |
| LSY12010300808 | 192288337 | 0.374 | 0.99 | 42 | 0.86 | 0.95 | 0.98 |
| LSY12010300936 | 187842998 | 0.3841 | 0.99 | 42 | 0.88 | 0.95 | 0.98 |
|  |  |  |  |  |  |  |  |
| LSY12030105094 | 227512515 | 0.3174 | 0.99 | 42 | 0.92 | 0.97 | 0.98 |
| IS020 | 116095849 | 0.6173 | 0.99 | 41 | 0.88 | 0.95 | 0.98 |
| IS435 | 148922920 | 0.4724 | 0.99 | 41 | 0.87 | 0.95 | 0.97 |
| LSY2550 | 108540416 | 0.6516 | 0.99 | 41 | 0.89 | 0.96 | 0.98 |
| LSY3068 | 174845877 | 0.4048 | 0.99 | 41 | 0.9 | 0.96 | 0.98 |
| LSY12010202422 | 159970442 | 0.4432 | 0.99 | 41 | 0.81 | 0.93 | 0.98 |
| LSY12030105467 | 201964356 | 0.3523 | 0.99 | 41 | 0.9 | 0.97 | 0.98 |
| S31 | 222274920 | 0.3076 | 0.98 | 40 | 0.8 | 0.91 | 0.96 |
| S69 | 275325856 | 0.2523 | 0.98 | 40 | 0.83 | 0.92 | 0.96 |
| LSY12010202256 | 168877740 | 0.4132 | 0.99 | 40 | 0.79 | 0.92 | 0.97 |
| LSY12010202274 | 157799466 | 0.4434 | 0.99 | 40 | 0.73 | 0.88 | 0.96 |
| LSY12010300465 | 219753630 | 0.3148 | 0.99 | 40 | 0.89 | 0.96 | 0.98 |
| LSY12010300689 | 184856900 | 0.3754 | 0.99 | 40 | 0.87 | 0.95 | 0.98 |
| LSY12010302692 | 269840326 | 0.2592 | 0.99 | 40 | 0.88 | 0.95 | 0.97 |
| LSY12030105702 | 195370139 | 0.3543 | 0.99 | 40 | 0.89 | 0.96 | 0.98 |
| IS033 | 168848139 | 0.4028 | 0.99 | 39 | 0.85 | 0.95 | 0.98 |
| IS206 | 242874358 | 0.2757 | 0.99 | 39 | 0.89 | 0.96 | 0.98 |
| IS222 | 156536038 | 0.4351 | 0.99 | 39 | 0.85 | 0.94 | 0.98 |
| LSY1544 | 150285118 | 0.4527 | 0.99 | 39 | 0.87 | 0.95 | 0.98 |
| LSY12010202324 | 176333786 | 0.384 | 0.99 | 39 | 0.79 | 0.92 | 0.98 |
| LSY12010300492 | 162745467 | 0.4177 | 0.99 | 39 | 0.85 | 0.95 | 0.97 |
| LSY12010301157 | 137808004 | 0.485 | 0.99 | 39 | 0.83 | 0.94 | 0.98 |
| LSY12030105413 | 266419129 | 0.2543 | 0.99 | 39 | 0.93 | 0.97 | 0.98 |
| LSY12030105710 | 242157238 | 0.2774 | 0.99 | 39 | 0.91 | 0.96 | 0.98 |
| S20 | 195814814 | 0.3316 | 0.99 | 38 | 0.82 | 0.92 | 0.97 |
| IS050 | 156541050 | 0.4178 | 0.99 | 38 | 0.81 | 0.93 | 0.97 |
| IS145 | 160782785 | 0.409 | 0.99 | 38 | 0.8 | 0.92 | 0.97 |
| LSY12010202482 | 149551488 | 0.4448 | 0.99 | 38 | 0.86 | 0.96 | 0.98 |
| LSY12010300868 | 182566742 | 0.3629 | 0.99 | 38 | 0.85 | 0.94 | 0.97 |
| S35 | 263063767 | 0.2386 | 0.98 | 37 | 0.74 | 0.88 | 0.95 |
| IS361 | 158664416 | 0.4012 | 0.99 | 37 | 0.85 | 0.95 | 0.97 |
| LSY2827 | 154276633 | 0.4216 | 0.99 | 37 | 0.89 | 0.96 | 0.98 |
| LSY2846 | 163555392 | 0.3929 | 0.98 | 37 | 0.66 | 0.77 | 0.9 |
| LSY12010302586 | 263112633 | 0.2462 | 0.99 | 37 | 0.88 | 0.96 | 0.98 |
| LSY12030105659 | 287820750 | 0.2238 | 0.99 | 37 | 0.91 | 0.97 | 0.98 |
| TXRY12E0311 | 130365058 | 0.4738 | 0.98 | 36 | 0.74 | 0.85 | 0.94 |
| LSY5465 | 141448001 | 0.4334 | 0.97 | 36 | 0.73 | 0.82 | 0.9 |
| LSY12010301752 | 166746092 | 0.3778 | 0.98 | 36 | 0.81 | 0.92 | 0.96 |
| LSY12030104975 | 207185476 | 0.3028 | 0.99 | 36 | 0.88 | 0.96 | 0.98 |
| IS312 | 152643816 | 0.4 | 0.98 | 35 | 0.83 | 0.94 | 0.97 |
| LSY12010300882 | 141716474 | 0.4291 | 0.99 | 35 | 0.84 | 0.94 | 0.97 |
| LSY12010301300 | 142005378 | 0.4245 | 0.99 | 35 | 0.85 | 0.95 | 0.97 |
| S21 | 242574045 | 0.2455 | 0.99 | 34 | 0.63 | 0.84 | 0.96 |
| IS302 | 205652752 | 0.2848 | 0.99 | 34 | 0.87 | 0.96 | 0.98 |
| IS308 | 144662316 | 0.4096 | 0.99 | 34 | 0.82 | 0.94 | 0.97 |
| IS443 | 123096536 | 0.4836 | 0.98 | 34 | 0.83 | 0.94 | 0.97 |
| LSY12010202225 | 133050002 | 0.4401 | 0.99 | 34 | 0.77 | 0.92 | 0.97 |
| S44 | 186298710 | 0.3077 | 0.98 | 33 | 0.72 | 0.88 | 0.95 |
| S66 | 347544318 | 0.1657 | 0.98 | 33 | 0.79 | 0.92 | 0.96 |
| LSY0622 | 142887824 | 0.3919 | 0.98 | 33 | 0.71 | 0.85 | 0.93 |
| LSY4817 | 193862129 | 0.2862 | 0.95 | 33 | 0.55 | 0.64 | 0.79 |
| LSY12010202242 | 137830355 | 0.4132 | 0.99 | 33 | 0.74 | 0.9 | 0.96 |
| LSY12010202588 | 126955135 | 0.4575 | 0.99 | 33 | 0.85 | 0.95 | 0.98 |
| LSY12010300645 | 125276893 | 0.4525 | 0.98 | 33 | 0.79 | 0.93 | 0.97 |
| LSY12030104040 | 179971714 | 0.3229 | 0.99 | 33 | 0.84 | 0.94 | 0.98 |
| LSY12030104584 | 120392415 | 0.4825 | 0.99 | 33 | 0.82 | 0.94 | 0.98 |
| LSY12030104940 | 124213565 | 0.4598 | 0.99 | 33 | 0.8 | 0.93 | 0.97 |
| S68 | 249578072 | 0.2236 | 0.98 | 32 | 0.78 | 0.91 | 0.96 |
| S73 | 315051534 | 0.1786 | 0.98 | 32 | 0.81 | 0.92 | 0.96 |
| LSY12010301884 | 125313155 | 0.4496 | 0.99 | 32 | 0.77 | 0.91 | 0.97 |
| LSY12030104103 | 172584145 | 0.3144 | 0.98 | 32 | 0.75 | 0.88 | 0.94 |
| LSY12030105632 | 133202207 | 0.4217 | 0.99 | 32 | 0.82 | 0.95 | 0.98 |
| S42 | 279790077 | 0.1901 | 0.98 | 31 | 0.75 | 0.91 | 0.96 |
| S77 | 206372300 | 0.2597 | 0.98 | 31 | 0.76 | 0.9 | 0.95 |
| IS208 | 155031580 | 0.3434 | 0.99 | 31 | 0.82 | 0.94 | 0.97 |
| IS318 | 126380672 | 0.424 | 0.99 | 31 | 0.85 | 0.96 | 0.98 |
| IS469 | 129149888 | 0.4143 | 0.98 | 31 | 0.78 | 0.92 | 0.97 |
| LSY1056 | 109484664 | 0.4625 | 0.94 | 31 | 0.63 | 0.72 | 0.82 |
| LSY4221 | 128094196 | 0.4201 | 0.98 | 31 | 0.78 | 0.9 | 0.95 |
| LSY5411 | 148229088 | 0.358 | 0.97 | 31 | 0.62 | 0.75 | 0.87 |
| LSY7691 | 158437918 | 0.3396 | 0.99 | 31 | 0.78 | 0.91 | 0.96 |
| LSY12010202533 | 129077909 | 0.4204 | 0.99 | 31 | 0.73 | 0.9 | 0.96 |
| LSY12030104229 | 176161150 | 0.3057 | 0.99 | 31 | 0.8 | 0.93 | 0.97 |
| LSY12030105002 | 180213624 | 0.2951 | 0.99 | 31 | 0.82 | 0.95 | 0.98 |
| TXRY12E0301 | 121896112 | 0.4264 | 0.99 | 30 | 0.67 | 0.86 | 0.96 |
| IS274 | 127073432 | 0.412 | 0.99 | 30 | 0.76 | 0.92 | 0.97 |
| LSY12010202224 | 118997165 | 0.4355 | 0.99 | 30 | 0.68 | 0.86 | 0.96 |
| LSY12010300471 | 170165722 | 0.3047 | 0.99 | 30 | 0.78 | 0.92 | 0.97 |
| LSY12030104241 | 115706396 | 0.4553 | 0.98 | 30 | 0.77 | 0.91 | 0.97 |
| S70 | 372137278 | 0.1334 | 0.99 | 29 | 0.77 | 0.93 | 0.97 |
| S75 | 261592166 | 0.1941 | 0.98 | 29 | 0.75 | 0.9 | 0.96 |
| IS316 | 189375455 | 0.2711 | 0.99 | 29 | 0.83 | 0.95 | 0.98 |
| IS442 | 101834915 | 0.4887 | 0.98 | 29 | 0.77 | 0.93 | 0.97 |
| LSY12010202328 | 126588692 | 0.4011 | 0.99 | 29 | 0.7 | 0.89 | 0.96 |
| LSY12010202596 | 120804647 | 0.4228 | 0.99 | 29 | 0.69 | 0.88 | 0.96 |
| LSY12030104270 | 147060639 | 0.3393 | 0.98 | 29 | 0.73 | 0.87 | 0.94 |
| TXRY12E0341 | 110835742 | 0.4318 | 0.97 | 28 | 0.57 | 0.77 | 0.91 |
| S67 | 246502179 | 0.1963 | 0.98 | 28 | 0.73 | 0.9 | 0.96 |
| IS087 | 112761729 | 0.4251 | 0.98 | 28 | 0.72 | 0.91 | 0.96 |
| IS192 | 125084192 | 0.3952 | 0.99 | 28 | 0.71 | 0.9 | 0.97 |
| IS269 | 117751141 | 0.4139 | 0.98 | 28 | 0.76 | 0.93 | 0.97 |
| LSY2691 | 184677574 | 0.2603 | 0.97 | 28 | 0.68 | 0.84 | 0.92 |
| LSY12010202464 | 99897249 | 0.4826 | 0.99 | 28 | 0.64 | 0.84 | 0.95 |
| LSY12030105353 | 166847595 | 0.2971 | 0.99 | 28 | 0.81 | 0.95 | 0.98 |
| S25 | 248134382 | 0.1904 | 0.98 | 27 | 0.7 | 0.9 | 0.96 |
| IS102 | 97838376 | 0.4801 | 0.99 | 27 | 0.74 | 0.93 | 0.97 |
| LSY4007 | 129327444 | 0.3603 | 0.96 | 27 | 0.5 | 0.63 | 0.82 |
| LSY5361 | 127138568 | 0.3702 | 0.98 | 27 | 0.71 | 0.88 | 0.95 |
| LSY12030104019 | 217798283 | 0.2156 | 0.99 | 27 | 0.78 | 0.95 | 0.98 |
| LSY12030104995 | 141239186 | 0.3314 | 0.99 | 27 | 0.75 | 0.93 | 0.97 |
|  |  |  |  |  |  |  |  |
| LSY12030106129 | 153994559 | 0.3029 | 0.99 | 27 | 0.76 | 0.94 | 0.98 |
| S52 | 255611861 | 0.1776 | 0.98 | 26 | 0.56 | 0.75 | 0.91 |
| IS261 | 132566216 | 0.341 | 0.98 | 26 | 0.74 | 0.93 | 0.97 |
| LSY2594 | 82792220 | 0.5464 | 0.98 | 26 | 0.73 | 0.93 | 0.97 |
| LSY5041 | 114989935 | 0.3848 | 0.98 | 26 | 0.68 | 0.86 | 0.94 |
| LSY12010300860 | 111088049 | 0.4051 | 0.99 | 26 | 0.72 | 0.93 | 0.97 |
| LSY12030104123 | 129199346 | 0.3507 | 0.99 | 26 | 0.74 | 0.92 | 0.97 |
| LSY12030104364 | 112890666 | 0.3943 | 0.98 | 26 | 0.7 | 0.91 | 0.97 |
| LSY12030105246 | 137934531 | 0.3344 | 0.99 | 26 | 0.75 | 0.93 | 0.97 |
| S28 | 107187515 | 0.3971 | 0.97 | 25 | 0.6 | 0.83 | 0.93 |
| S39 | 178398925 | 0.2452 | 0.98 | 25 | 0.64 | 0.86 | 0.94 |
| LSY12010202389 | 109881303 | 0.4036 | 0.99 | 25 | 0.65 | 0.88 | 0.96 |
| LSY12010301915 | 94498655 | 0.4617 | 0.98 | 25 | 0.68 | 0.91 | 0.97 |
| LSY12030105382 | 148079266 | 0.2961 | 0.99 | 25 | 0.76 | 0.95 | 0.98 |
| TXRY12E0161 | 94943060 | 0.4368 | 0.96 | 24 | 0.52 | 0.73 | 0.87 |
| S49 | 209501984 | 0.1989 | 0.97 | 24 | 0.6 | 0.84 | 0.94 |
| S74 | 160772239 | 0.2527 | 0.97 | 24 | 0.62 | 0.85 | 0.94 |
| S82 | 127751018 | 0.3277 | 0.98 | 24 | 0.62 | 0.85 | 0.94 |
| IS372 | 97668023 | 0.4257 | 0.98 | 24 | 0.65 | 0.9 | 0.96 |
| LSY4190 | 88973428 | 0.4661 | 0.99 | 24 | 0.64 | 0.89 | 0.97 |
| TXRY12E0298 | 95915643 | 0.4117 | 0.98 | 23 | 0.58 | 0.84 | 0.95 |
| S26 | 185591814 | 0.2113 | 0.98 | 23 | 0.57 | 0.82 | 0.94 |
| S27 | 173639064 | 0.2255 | 0.97 | 23 | 0.56 | 0.81 | 0.93 |
| S55 | 336070886 | 0.1187 | 0.99 | 23 | 0.56 | 0.81 | 0.94 |
| IS193 | 95502578 | 0.4216 | 0.99 | 23 | 0.64 | 0.9 | 0.97 |
| LSY4581 | 94155391 | 0.4185 | 0.97 | 23 | 0.55 | 0.78 | 0.91 |
| LSY12010202470 | 107694292 | 0.3749 | 0.98 | 23 | 0.58 | 0.83 | 0.95 |
| LSY12010300628 | 153137980 | 0.2663 | 0.99 | 23 | 0.66 | 0.91 | 0.97 |
| LSY12010300634 | 142079034 | 0.2869 | 0.98 | 23 | 0.64 | 0.9 | 0.97 |
| LSY12010302154 | 100986059 | 0.3972 | 0.98 | 23 | 0.6 | 0.88 | 0.96 |
| LSY12030105033 | 128815721 | 0.3159 | 0.99 | 23 | 0.67 | 0.92 | 0.97 |
| S65 | 302858289 | 0.1264 | 0.98 | 22 | 0.58 | 0.84 | 0.94 |
| LSY5033 | 89387747 | 0.4329 | 0.99 | 22 | 0.58 | 0.85 | 0.95 |
| LSY12010202365 | 96931599 | 0.4012 | 0.98 | 22 | 0.55 | 0.82 | 0.94 |
| LSY12010202437 | 88483606 | 0.4392 | 0.99 | 22 | 0.56 | 0.83 | 0.95 |
| LSY12010202443 | 90446801 | 0.4145 | 0.98 | 22 | 0.54 | 0.82 | 0.94 |
| LSY12010202453 | 85633159 | 0.4405 | 0.98 | 22 | 0.49 | 0.75 | 0.91 |
| LSY12010202607 | 85170562 | 0.4542 | 0.98 | 22 | 0.51 | 0.76 | 0.92 |
| LSY12030103995 | 117325504 | 0.3301 | 0.98 | 22 | 0.61 | 0.89 | 0.96 |
| LSY12030105111 | 120796571 | 0.322 | 0.99 | 22 | 0.63 | 0.92 | 0.97 |
| LSY12030105533 | 97542288 | 0.3913 | 0.99 | 22 | 0.6 | 0.89 | 0.97 |
| TXRY12E0200 | 86209583 | 0.4194 | 0.98 | 21 | 0.49 | 0.78 | 0.93 |
| IS229 | 146278696 | 0.2534 | 0.99 | 21 | 0.58 | 0.91 | 0.97 |
| IS352 | 78891578 | 0.4664 | 0.98 | 21 | 0.56 | 0.88 | 0.96 |
| LSY2347 | 77194928 | 0.473 | 0.99 | 21 | 0.58 | 0.9 | 0.97 |
| LSY12030104945 | 101430197 | 0.363 | 0.99 | 21 | 0.58 | 0.91 | 0.97 |
| LSY12030105056 | 121517822 | 0.308 | 0.99 | 21 | 0.6 | 0.91 | 0.97 |
| LSY12030106365 | 134785959 | 0.2697 | 0.99 | 21 | 0.56 | 0.89 | 0.96 |
| S24 | 261203747 | 0.1322 | 0.98 | 20 | 0.49 | 0.8 | 0.94 |
| S59 | 298421470 | 0.1168 | 0.98 | 20 | 0.53 | 0.86 | 0.96 |
| S60 | 217528602 | 0.1629 | 0.98 | 20 | 0.52 | 0.82 | 0.94 |
| LSY12010202564 | 78879843 | 0.4481 | 0.98 | 20 | 0.48 | 0.77 | 0.92 |
| LSY12010300510 | 134492663 | 0.2652 | 0.99 | 20 | 0.55 | 0.9 | 0.97 |
| LSY12010301912 | 77539828 | 0.4391 | 0.98 | 20 | 0.5 | 0.86 | 0.96 |
| S18 | 182788890 | 0.1847 | 0.97 | 19 | 0.48 | 0.78 | 0.93 |
| S92 | 127417638 | 0.2605 | 0.97 | 19 | 0.47 | 0.81 | 0.93 |
| IS218 | 132729551 | 0.2466 | 0.99 | 19 | 0.46 | 0.89 | 0.97 |
| IS421 | 213704671 | 0.1544 | 0.99 | 19 | 0.47 | 0.91 | 0.97 |
| IS461 | 69647137 | 0.4766 | 0.98 | 19 | 0.47 | 0.85 | 0.96 |
| LSY12010202302 | 83564514 | 0.3989 | 0.98 | 19 | 0.46 | 0.79 | 0.94 |
| LSY12010202500 | 64102281 | 0.5115 | 0.98 | 19 | 0.43 | 0.74 | 0.92 |
| LSY12030104444 | 79580107 | 0.4243 | 0.98 | 19 | 0.49 | 0.86 | 0.96 |
| LSY12030105521 | 131822444 | 0.2571 | 0.98 | 19 | 0.5 | 0.89 | 0.97 |
| LSY12030106024 | 110456255 | 0.302 | 0.99 | 19 | 0.48 | 0.91 | 0.98 |
| TXRY12E0386 | 74515147 | 0.4244 | 0.98 | 18 | 0.43 | 0.81 | 0.95 |
| S46 | 145906196 | 0.2149 | 0.98 | 18 | 0.43 | 0.8 | 0.94 |
| IS052 | 136264403 | 0.2368 | 0.99 | 18 | 0.45 | 0.9 | 0.97 |
| S71 | 246902629 | 0.1288 | 0.98 | 18 | 0.46 | 0.81 | 0.94 |
| IS417 | 201204933 | 0.1575 | 0.99 | 18 | 0.43 | 0.9 | 0.98 |
| IS497 | 55014621 | 0.5661 | 0.98 | 18 | 0.42 | 0.85 | 0.96 |
| LSY4457 | 74518082 | 0.4185 | 0.99 | 18 | 0.42 | 0.82 | 0.95 |
| LSY12010202527 | 77620744 | 0.4152 | 0.98 | 18 | 0.43 | 0.75 | 0.91 |
| LSY12030105504 | 91885313 | 0.3375 | 0.99 | 18 | 0.41 | 0.87 | 0.97 |
| LSY12030105657 | 142389099 | 0.2249 | 0.98 | 18 | 0.44 | 0.88 | 0.97 |
| LSY12030106042 | 105294886 | 0.2978 | 0.99 | 18 | 0.41 | 0.9 | 0.98 |
| LSY12030106247 | 137467183 | 0.2326 | 0.99 | 18 | 0.44 | 0.89 | 0.97 |
| LSY12030106408 | 109800338 | 0.2819 | 0.99 | 18 | 0.4 | 0.89 | 0.98 |
| S32 | 78816908 | 0.3637 | 0.97 | 17 | 0.37 | 0.73 | 0.91 |
| IS224 | 79905589 | 0.375 | 0.98 | 17 | 0.39 | 0.82 | 0.95 |
| LSY4478 | 70342285 | 0.4088 | 0.97 | 17 | 0.38 | 0.73 | 0.89 |
| LSY5207 | 93021593 | 0.3127 | 0.99 | 17 | 0.36 | 0.83 | 0.96 |
| LSY5479 | 66009335 | 0.4338 | 0.97 | 17 | 0.37 | 0.73 | 0.9 |
| LSY12010202396 | 64487103 | 0.4708 | 0.98 | 17 | 0.4 | 0.79 | 0.95 |
| LSY12010202541 | 66602464 | 0.4351 | 0.98 | 17 | 0.36 | 0.76 | 0.93 |
| LSY12030104748 | 68233625 | 0.4415 | 0.98 | 17 | 0.39 | 0.83 | 0.96 |
| LSY12030105957 | 93110926 | 0.3183 | 0.99 | 17 | 0.36 | 0.87 | 0.97 |
| S16 | 95750180 | 0.2997 | 0.97 | 16 | 0.36 | 0.74 | 0.92 |
| S62 | 222564783 | 0.1233 | 0.97 | 16 | 0.33 | 0.74 | 0.92 |
| S72 | 161118983 | 0.1721 | 0.97 | 16 | 0.36 | 0.74 | 0.91 |
| S86 | 172368727 | 0.1624 | 0.97 | 16 | 0.35 | 0.72 | 0.91 |
| S94 | 152311040 | 0.1857 | 0.97 | 16 | 0.35 | 0.75 | 0.92 |
| LSY1780 | 65512909 | 0.4397 | 0.98 | 16 | 0.35 | 0.78 | 0.94 |
| LSY3724 | 89815184 | 0.3037 | 0.98 | 16 | 0.29 | 0.82 | 0.96 |
| LSY3853 | 107043771 | 0.2635 | 0.99 | 16 | 0.32 | 0.83 | 0.96 |
| LSY5517 | 59097356 | 0.4567 | 0.97 | 16 | 0.33 | 0.72 | 0.89 |
| LSY12010300472 | 78629739 | 0.3511 | 0.98 | 16 | 0.31 | 0.8 | 0.95 |
| LSY12010300745 | 100496032 | 0.2873 | 0.98 | 16 | 0.35 | 0.8 | 0.95 |
| LSY12010300795 | 87745412 | 0.3171 | 0.98 | 16 | 0.32 | 0.78 | 0.95 |
| LSY12030104102 | 67201155 | 0.4024 | 0.97 | 16 | 0.32 | 0.76 | 0.91 |
| LSY12030104115 | 86971995 | 0.3259 | 0.98 | 16 | 0.35 | 0.79 | 0.93 |
|  |  |  |  |  |  |  |  |
| LSY12030104269 | 79414161 | 0.3487 | 0.98 | 16 | 0.32 | 0.8 | 0.94 |
| LSY12030104633 | 65208959 | 0.4208 | 0.98 | 16 | 0.31 | 0.79 | 0.95 |
| LSY12030105603 | 115880635 | 0.24 | 0.99 | 16 | 0.3 | 0.83 | 0.96 |
| TXRY12E0381 | 62312246 | 0.4109 | 0.98 | 15 | 0.28 | 0.7 | 0.91 |
| S17 | 126186927 | 0.2054 | 0.98 | 15 | 0.29 | 0.7 | 0.91 |
| S36 | 85069333 | 0.3022 | 0.96 | 15 | 0.3 | 0.67 | 0.88 |
| S40 | 127897373 | 0.2041 | 0.97 | 15 | 0.3 | 0.71 | 0.91 |
| S53 | 184507282 | 0.1443 | 0.98 | 15 | 0.29 | 0.77 | 0.94 |
| S79 | 193426061 | 0.1357 | 0.97 | 15 | 0.3 | 0.73 | 0.92 |
| IS213 | 66222112 | 0.3935 | 0.98 | 15 | 0.24 | 0.8 | 0.96 |
| IS310 | 119714319 | 0.2223 | 0.99 | 15 | 0.26 | 0.8 | 0.96 |
| IS346 | 60437170 | 0.4216 | 0.98 | 15 | 0.26 | 0.73 | 0.93 |
| IS405 | 170450909 | 0.1566 | 0.98 | 15 | 0.25 | 0.83 | 0.97 |
| LSY4850 | 56688669 | 0.473 | 0.97 | 15 | 0.31 | 0.72 | 0.91 |
| LSY12010202566 | 66442911 | 0.4034 | 0.98 | 15 | 0.3 | 0.74 | 0.93 |
| LSY12010300473 | 95602631 | 0.2734 | 0.98 | 15 | 0.26 | 0.78 | 0.95 |
| LSY12030105355 | 80405215 | 0.3341 | 0.99 | 15 | 0.26 | 0.83 | 0.96 |
| LSY12030105537 | 96840180 | 0.2784 | 0.98 | 15 | 0.28 | 0.8 | 0.95 |
| IS007 | 89284391 | 0.2813 | 0.98 | 14 | 0.21 | 0.78 | 0.96 |
| S51 | 206636524 | 0.1172 | 0.98 | 14 | 0.22 | 0.71 | 0.93 |
| S56 | 204916336 | 0.1164 | 0.98 | 14 | 0.23 | 0.67 | 0.91 |
| IS066 | 76088514 | 0.323 | 0.98 | 14 | 0.19 | 0.78 | 0.96 |
| S84 | 143900712 | 0.1753 | 0.98 | 14 | 0.24 | 0.75 | 0.93 |
| S90 | 99830712 | 0.2455 | 0.97 | 14 | 0.25 | 0.7 | 0.91 |
| IS317 | 100103401 | 0.2468 | 0.98 | 14 | 0.19 | 0.78 | 0.96 |
| LSY5161 | 60060401 | 0.4194 | 0.97 | 14 | 0.28 | 0.69 | 0.89 |
| LSY12030104032 | 114019325 | 0.2176 | 0.98 | 14 | 0.22 | 0.75 | 0.95 |
| LSY12030104092 | 66175832 | 0.3761 | 0.98 | 14 | 0.23 | 0.75 | 0.93 |
| LSY12030104774 | 51032681 | 0.4854 | 0.98 | 14 | 0.23 | 0.73 | 0.93 |
| LSY12030106038 | 64597349 | 0.3731 | 0.99 | 14 | 0.17 | 0.78 | 0.97 |
| LSY12030106132 | 70619371 | 0.3541 | 0.99 | 14 | 0.2 | 0.79 | 0.96 |
| TXRY12E0124 | 49741559 | 0.4358 | 0.95 | 13 | 0.19 | 0.62 | 0.84 |
| S57 | 195074104 | 0.1188 | 0.97 | 13 | 0.19 | 0.7 | 0.92 |
| S87 | 97211514 | 0.2328 | 0.97 | 13 | 0.19 | 0.66 | 0.91 |
| IS276 | 54745398 | 0.4047 | 0.98 | 13 | 0.15 | 0.67 | 0.93 |
| IS315 | 87175263 | 0.2559 | 0.98 | 13 | 0.13 | 0.71 | 0.95 |
| LSY4415 | 100531138 | 0.2101 | 0.91 | 13 | 0.21 | 0.6 | 0.78 |
| LSY4418 | 49495974 | 0.4459 | 0.98 | 13 | 0.16 | 0.67 | 0.92 |
| LSY4887 | 54113786 | 0.4127 | 0.96 | 13 | 0.19 | 0.67 | 0.87 |
| LSY12010202308 | 56639648 | 0.3908 | 0.98 | 13 | 0.17 | 0.65 | 0.91 |
| LSY12030104942 | 38034361 | 0.6147 | 0.98 | 13 | 0.19 | 0.7 | 0.93 |
| IS002 | 72097185 | 0.2869 | 0.98 | 12 | 0.1 | 0.65 | 0.94 |
| S6 | 128660346 | 0.1571 | 0.96 | 12 | 0.17 | 0.53 | 0.83 |
| S12 | 119873813 | 0.169 | 0.96 | 12 | 0.22 | 0.41 | 0.73 |
| S45 | 125551072 | 0.1632 | 0.97 | 12 | 0.15 | 0.58 | 0.88 |
| IS072 | 91952685 | 0.2296 | 0.98 | 12 | 0.1 | 0.68 | 0.95 |
| LSY2038 | 51743113 | 0.3897 | 0.94 | 12 | 0.17 | 0.57 | 0.82 |
| LSY5364 | 125209500 | 0.1691 | 0.97 | 12 | 0.14 | 0.63 | 0.9 |
| LSY5484 | 47374371 | 0.4365 | 0.97 | 12 | 0.15 | 0.6 | 0.87 |
| LSY12030104719 | 39002287 | 0.5505 | 0.98 | 12 | 0.13 | 0.66 | 0.92 |
| LSY12030105950 | 71208655 | 0.3037 | 0.99 | 12 | 0.1 | 0.71 | 0.96 |
| TXRY12E0117 | 37874411 | 0.5014 | 0.98 | 11 | 0.1 | 0.56 | 0.88 |
| TXRY12E0244 | 43696452 | 0.4209 | 0.96 | 11 | 0.09 | 0.55 | 0.85 |
| TXRY12E0327 | 47803378 | 0.4027 | 0.96 | 11 | 0.11 | 0.57 | 0.86 |
| S54 | 127875351 | 0.1515 | 0.95 | 11 | 0.14 | 0.55 | 0.82 |
| IS216 | 52965035 | 0.3561 | 0.98 | 11 | 0.06 | 0.59 | 0.93 |
| IS404 | 87281446 | 0.2219 | 0.98 | 11 | 0.06 | 0.61 | 0.94 |
| IS419 | 131660238 | 0.1505 | 0.98 | 11 | 0.07 | 0.63 | 0.94 |
| IS422 | 137259476 | 0.1387 | 0.98 | 11 | 0.06 | 0.59 | 0.93 |
| LSY2538 | 47475549 | 0.3893 | 0.96 | 11 | 0.09 | 0.54 | 0.86 |
| LSY4876 | 50786349 | 0.383 | 0.94 | 11 | 0.15 | 0.56 | 0.81 |
| LSY12010202309 | 42823465 | 0.4372 | 0.98 | 11 | 0.09 | 0.55 | 0.89 |
| LSY12010302351 | 35335868 | 0.537 | 0.98 | 11 | 0.07 | 0.58 | 0.91 |
| LSY12030104545 | 35429747 | 0.5431 | 0.98 | 11 | 0.09 | 0.58 | 0.9 |
| LSY12030105773 | 81242306 | 0.2403 | 0.99 | 11 | 0.06 | 0.62 | 0.94 |
| LSY12030106055 | 84604056 | 0.24 | 0.98 | 11 | 0.09 | 0.64 | 0.93 |
| LSY12030106367 | 45537493 | 0.4312 | 0.97 | 11 | 0.11 | 0.59 | 0.88 |
| TXRY12E0348 | 48508170 | 0.3827 | 0.98 | 10 | 0.09 | 0.55 | 0.88 |
| S76 | 143452436 | 0.1246 | 0.98 | 10 | 0.04 | 0.55 | 0.91 |
| S80 | 126506244 | 0.1334 | 0.97 | 10 | 0.05 | 0.49 | 0.86 |
| S85 | 143626852 | 0.1198 | 0.96 | 10 | 0.06 | 0.51 | 0.86 |
| S91 | 55767140 | 0.3205 | 0.96 | 10 | 0.08 | 0.52 | 0.86 |
| S96 | 126442303 | 0.1404 | 0.97 | 10 | 0.06 | 0.52 | 0.89 |
| IS321 | 83510889 | 0.2197 | 0.98 | 10 | 0.05 | 0.56 | 0.93 |
| IS345 | 37859122 | 0.4557 | 0.97 | 10 | 0.05 | 0.5 | 0.89 |
| IS380 | 55194436 | 0.3071 | 0.97 | 10 | 0.05 | 0.49 | 0.87 |
| LSY3998 | 39810376 | 0.417 | 0.95 | 10 | 0.07 | 0.48 | 0.81 |
| LSY5451 | 40728151 | 0.4292 | 0.98 | 10 | 0.04 | 0.53 | 0.9 |
| LSY5539 | 36718768 | 0.4475 | 0.94 | 10 | 0.07 | 0.47 | 0.79 |
| LSY12030105138 | 69302439 | 0.2461 | 0.98 | 10 | 0.04 | 0.5 | 0.91 |
| LSY12030105589 | 74134713 | 0.2324 | 0.98 | 10 | 0.04 | 0.51 | 0.9 |
| LSY12030105886 | 81493617 | 0.2262 | 0.99 | 10 | 0.05 | 0.57 | 0.94 |
| LSY12030106069 | 59612920 | 0.2914 | 0.98 | 10 | 0.04 | 0.52 | 0.91 |
| TXRY12E0113 | 34175126 | 0.4948 | 0.98 | 9 | 0.03 | 0.5 | 0.91 |
| TXRY12E0157 | 40034895 | 0.401 | 0.98 | 9 | 0.03 | 0.46 | 0.88 |
| TXRY12E0238 | 39442295 | 0.4176 | 0.97 | 9 | 0.05 | 0.46 | 0.86 |
| TXRY12E0276 | 38874132 | 0.4021 | 0.97 | 9 | 0.03 | 0.44 | 0.86 |
| S19 | 107310884 | 0.1513 | 0.96 | 9 | 0.06 | 0.45 | 0.83 |
| S88 | 121169299 | 0.1287 | 0.96 | 9 | 0.05 | 0.43 | 0.82 |
| LSY2102 | 40009009 | 0.4177 | 0.97 | 9 | 0.05 | 0.47 | 0.87 |
| LSY12030105956 | 74337557 | 0.2244 | 0.98 | 9 | 0.03 | 0.48 | 0.91 |
| LSY12030106182 | 68191790 | 0.246 | 0.98 | 9 | 0.03 | 0.49 | 0.9 |
| S11 | 133741036 | 0.1092 | 0.96 | 8 | 0.03 | 0.38 | 0.81 |
| TXRY12E0163 | 32725478 | 0.4341 | 0.97 | 8 | 0.01 | 0.37 | 0.84 |
| S78 | 111223196 | 0.1331 | 0.96 | 8 | 0.03 | 0.4 | 0.82 |
| S93 | 110620796 | 0.1225 | 0.95 | 8 | 0.02 | 0.35 | 0.78 |
| S95 | 84328445 | 0.1732 | 0.97 | 8 | 0.02 | 0.39 | 0.83 |
| IS325 | 50572512 | 0.2681 | 0.98 | 8 | 0.01 | 0.32 | 0.85 |
| LSY3919 | 34215423 | 0.3713 | 0.87 | 8 | 0.05 | 0.35 | 0.6 |
| LSY12030105256 | 45651378 | 0.3086 | 0.96 | 8 | 0.01 | 0.36 | 0.83 |
|  |  |  |  |  |  |  |  |
| LSY12030106007 | 61944407 | 0.2457 | 0.98 | 8 | 0.02 | 0.41 | 0.89 |
| S41 | 83844648 | 0.1482 | 0.95 | 7 | 0.01 | 0.28 | 0.76 |
| IS070 | 443250345 | 0.0288 | 0.97 | 7 | 0.01 | 0.28 | 0.82 |
| S83 | 61952306 | 0.1953 | 0.95 | 7 | 0.01 | 0.26 | 0.77 |
| IS425 | 82953457 | 0.1505 | 0.97 | 7 | 0 | 0.27 | 0.82 |
| LSY12030104643 | 27362633 | 0.4876 | 0.97 | 7 | 0.01 | 0.32 | 0.83 |
| LSY12030106157 | 39356693 | 0.3412 | 0.97 | 7 | 0.01 | 0.32 | 0.84 |
| LSY12030106518 | 397091633 | 0.0312 | 0.97 | 7 | 0.01 | 0.26 | 0.81 |
| TXRY12E0151 | 23091176 | 0.4759 | 0.94 | 6 | 0 | 0.22 | 0.72 |
| S58 | 85418872 | 0.1262 | 0.95 | 6 | 0 | 0.19 | 0.73 |
| IS084 | 35227675 | 0.2976 | 0.96 | 6 | 0 | 0.17 | 0.74 |
| IS122 | 35030389 | 0.3217 | 0.96 | 6 | 0 | 0.21 | 0.77 |
| IS418 | 71740936 | 0.1552 | 0.97 | 6 | 0 | 0.19 | 0.78 |
| LSY3497 | 22171872 | 0.4544 | 0.91 | 6 | 0 | 0.19 | 0.66 |
| LSY12010302348 | 76322453 | 0.1339 | 0.96 | 6 | 0 | 0.15 | 0.74 |
| LSY12030104569 | 22487803 | 0.5113 | 0.96 | 6 | 0 | 0.23 | 0.76 |
| LSY12030106080 | 235219235 | 0.0431 | 0.97 | 6 | 0 | 0.15 | 0.73 |
| TXRY12E0252 | 19385156 | 0.4543 | 0.93 | 5 | 0 | 0.11 | 0.63 |
| S22 | 56254571 | 0.144 | 0.92 | 5 | 0 | 0.09 | 0.58 |
| S47 | 65355283 | 0.1315 | 0.92 | 5 | 0 | 0.11 | 0.61 |
| LSY1920 | 22456258 | 0.4058 | 0.95 | 5 | 0 | 0.11 | 0.66 |
| LSY2106 | 21304884 | 0.4071 | 0.93 | 5 | 0 | 0.1 | 0.63 |
| LSY2153 | 22561676 | 0.4301 | 0.94 | 5 | 0 | 0.15 | 0.67 |
| LSY3630 | 24404943 | 0.3491 | 0.93 | 5 | 0 | 0.08 | 0.64 |
| LSY12030104296 | 33535777 | 0.2789 | 0.93 | 5 | 0 | 0.13 | 0.66 |
| LSY12030104492 | 21107473 | 0.4197 | 0.94 | 5 | 0 | 0.11 | 0.65 |
| LSY12030106114 | 185189583 | 0.0439 | 0.93 | 5 | 0 | 0.08 | 0.61 |
| LSY12030106515 | 512139153 | 0.0177 | 0.95 | 5 | 0 | 0.11 | 0.66 |
| TXRY12E0351 | 14276429 | 0.4293 | 0.88 | 4 | 0 | 0.03 | 0.44 |
| IS429 | 48429882 | 0.1441 | 0.9 | 4 | 0 | 0.05 | 0.52 |
| LSY2165 | 16687865 | 0.3843 | 0.9 | 4 | 0 | 0.03 | 0.48 |
| LSY5707 | 20883360 | 0.3431 | 0.91 | 4 | 0 | 0.04 | 0.55 |
| LSY12030106123 | 230436534 | 0.0284 | 0.91 | 4 | 0 | 0.04 | 0.48 |
| LSY12030106403 | 188408568 | 0.0371 | 0.9 | 4 | 0 | 0.05 | 0.52 |
| LSY12030106419 | 163736213 | 0.0391 | 0.89 | 4 | 0 | 0.03 | 0.47 |
| LSY12030106470 | 221507523 | 0.0336 | 0.94 | 4 | 0 | 0.05 | 0.56 |
| IS058 | 156243182 | 0.0366 | 0.86 | 3 | 0 | 0.02 | 0.41 |
| IS423 | 16731796 | 0.2603 | 0.83 | 3 | 0 | 0.01 | 0.27 |
| LSY12010300462 | 21896861 | 0.1805 | 0.74 | 3 | 0 | 0.01 | 0.25 |
| IS046 | 253017944 | 0.0133 | 0.67 | 2 | 0 | 0.01 | 0.2 |
| S61 | 22186564 | 0.1252 | 0.65 | 2 | 0 | 0 | 0.14 |
| LSY12010302653 | 21974177 | 0.1256 | 0.68 | 2 | 0 | 0 | 0.13 |
| LSY12030103968 | 35131453 | 0.0801 | 0.69 | 2 | 0 | 0 | 0.13 |
| LSY12030104005 | 28473284 | 0.0871 | 0.64 | 2 | 0 | 0 | 0.1 |
| LSY12030104420 | 9684102 | 0.4045 | 0.8 | 2 | 0 | 0 | 0.23 |
| LSY12030104481 | 9767758 | 0.4348 | 0.82 | 2 | 0 | 0.01 | 0.26 |
| LSY12030106512 | 296411329 | 0.0106 | 0.72 | 2 | 0 | 0 | 0.16 |
| S8 | 25131 | 0.4126 | 0.01 | 1 | 0 | 0 | 0 |
| S89 | 3656434 | 0.0482 | 0.09 | 1 | 0 | 0 | 0 |
| IS415 | 92785 | 0.3621 | 0.01 | 1 | 0 | 0 | 0 |
| LSY12010202240 | 285107 | 0.4543 | 0.06 | 1 | 0 | 0 | 0 |
| LSY12030104120 | 10863681 | 0.0906 | 0.36 | 1 | 0 | 0 | 0.02 |
|  |  |  |  |  |  |  |  |
|  |  |  |  |  |  |  |  |
