# Supplementary material for: Targeted sequencing of NOTCH signaling pathway genes and association analysis of variants correlated with mandibular prognathism
Source: Head Face Med. 2021 May 26;17:17. doi: 10.1186/s13005-021-00268-0 (PMC8152080; doi:10.1186/s13005-021-00268-0)
Supplement: Supplementary file 5 — Additional file 5. [file 13005_2021_268_MOESM5_ESM.docx]

**Table S5**. Summary of the principal components analysis

| **Principal component^a^** | **1** | **2** | | **3** | **4** | | | **5** |
| --- | --- | --- | --- | --- | --- | --- | --- | --- |
| Variance explained | 0.24269 | | 0.17498 | 0.13939 | | 0.09660 | | 0.07272 |
| Cumulative variance^b^ |  | | 0.41767 | 0.55706 | | 0.65366 | 0.72638 | |
| Variables^c^ | Yaxis (N-S-Gn) | | Mandibular Body Length | L1Protrusion LI:(APog) | | LI-MP (IMPA) | N to A through Horizontal Plane | |
|  | APDI(NP-FH) | | Posterior Facial Height (Co-Go) | FacialTaper | | FMIA | Na_\|_toA point | |
|  | Pg-Na  Perpendicular | | Ramus Height  (Ar-Go) | GonialAngle  (Ar-Go-Me) | | LI^Apog (L1  Protrusio) | N to B through Horizontal Plane | |
|  | ANB | | Anterior Facial Height (N-Me) | Lower Lip to E-Plane | | S-Go/N-Me (%) | N to Pog through Horizontal Plane | |
|  | Convexity  (NA-APg) | | Lower lip length (LLS-Me') | UI-LI (Interincisal Angle) | | UI-SN | SNA | |

a. represents the variance explained by each principal component in PCA

b. shows the cumulative variance explained by each added PC sequentially

c. displays the variables contributing the most in each PC
