# Supplementary material for: Targeted sequencing of NOTCH signaling pathway genes and association analysis of variants correlated with mandibular prognathism
Source: Head Face Med. 2021 May 26;17:17. doi: 10.1186/s13005-021-00268-0 (PMC8152080; doi:10.1186/s13005-021-00268-0)
Supplement: Supplementary file 6 — Additional file 6. [file 13005_2021_268_MOESM6_ESM.docx]

**Table S6.** Burden test of rare variants

| Chr/Gene | Location | SNP/*t* value | REF/ALT | MAF | Genotype | | | | | | | CADD  score |
| --- | --- | --- | --- | --- | --- | --- | --- | --- | --- | --- | --- | --- |
|  |  |  |  |  |  | Case |  |  |  | Control |  |  |
|  |  |  |  |  | Ref-Ref | Ref-Alt | Alt-Alt |  | Ref-Ref | Ref-Alt | Alt-Alt |  |
| 15 | 58903263 | . | C/T | 0.001272 | 194 | 0 | 0 |  | 198 | 1 | 0 | 24.9 |
| 15 | 58936150 | rs147572059 | T/C | 0.01023 | 189 | 3 | 0 |  | 194 | 5 | 0 | 7.358 |
| 15 | 58957379 | . | C/T | 0.001263 | 196 | 1 | 0 |  | 199 | 0 | 0 | 33 |
| 15 | 58974425 | . | T/A | 0.001312 | 183 | 1 | 0 |  | 197 | 0 | 0 | 24.2 |
| *ADAM10* | **0.700966** | **-0.38409** |  |  |  |  |  |  |  |  |  |  |
|  |  |  |  |  |  |  |  |  |  |  |  |  |
| 2 | 9630617 | . | C/T | 0.001263 | 196 | 1 | 0 |  | 199 | 0 | 0 | 16.9 |
| 2 | 9631256 | . | C/G | 0.001292 | 187 | 1 | 0 |  | 199 | 0 | 0 | 21.5 |
| 2 | 9645340 | . | G/A | 0.001263 | 197 | 0 | 0 |  | 198 | 1 | 0 | 15.89 |
| *ADAM17* | **-0.73406** | **0.463056** |  |  |  |  |  |  |  |  |  |  |
|  |  |  |  |  |  |  |  |  |  |  |  |  |
| 6 | 170592397 | . | G/A | 0.001263 | 196 | 1 | 0 |  | 199 | 0 | 0 | 1.928 |
| 6 | 170592574 | rs370005717 | C/T | 0.001263 | 197 | 0 | 0 |  | 198 | 1 | 0 | 29 |
| 6 | 170592637 | . | C/T | 0.001269 | 196 | 1 | 0 |  | 197 | 0 | 0 | 5.724 |
| 6 | 170592677 | . | C/T | 0.001279 | 195 | 1 | 0 |  | 195 | 0 | 0 | 14.5 |
| 6 | 170592958 | rs200081575 | G/A | 0.001309 | 192 | 1 | 0 |  | 189 | 0 | 0 | 16.52 |
| 6 | 170592983 | . | C/T | 0.003876 | 193 | 1 | 0 |  | 191 | 2 | 0 | 8.606 |
| 6 | 170593054 | . | C/T | 0.001269 | 196 | 1 | 0 |  | 197 | 0 | 0 | 0.025 |
| 6 | 170597823 | rs146990317 | C/T | 0.001266 | 196 | 1 | 0 |  | 198 | 0 | 0 | 3.484 |
| *DLL1* | **-0.113852** | **0.909363** |  |  |  |  |  |  |  |  |  |  |
|  |  |  |  |  |  |  |  |  |  |  |  |  |
| 19 | 39990003 | . | G/A | 0.004043 | 184 | 2 | 0 |  | 184 | 1 | 0 | 4.682 |
| 19 | 39994804 | . | C/T | 0.001266 | 197 | 0 | 0 |  | 197 | 1 | 0 | 12.87 |
| 19 | 39994863 | rs139297205 | G/A | 0.001266 | 197 | 0 | 0 |  | 197 | 1 | 0 | 4.072 |
| 19 | 39997906 | . | G/T | 0.006098 | 51 | 0 | 0 |  | 30 | 1 | 0 | 31 |
| 19 | 39998571 | . | T/C | 0.001263 | 197 | 0 | 0 |  | 198 | 1 | 0 | 9.947 |
| *DLL3* | **1.49384** | **0.13541** |  |  |  |  |  |  |  |  |  |  |
|  |  |  |  |  |  |  |  |  |  |  |  |  |
| 15 | 41227100 | . | A/G | 0.002525 | 196 | 1 | 0 |  | 198 | 1 | 0 | 12.8 |
| 15 | 41228746 | rs189323864 | G/A | 0.013995 | 190 | 6 | 0 |  | 192 | 5 | 0 | 10.2 |
| 15 | 41229699 | rs200998312 | G/A | 0.001266 | 196 | 0 | 0 |  | 198 | 1 | 0 | 9.437 |
| *DLL4* | **-0.0383886** | **0.969384** |  |  |  |  |  |  |  |  |  |  |
|  |  |  |  |  |  |  |  |  |  |  |  |  |
| 12 | 113515348 | rs201845055 | A/G | 0.001276 | 196 | 1 | 0 |  | 195 | 0 | 0 | 11.22 |
| 12 | 113515539 | . | G/C | 0.001348 | 190 | 0 | 0 |  | 180 | 1 | 0 | 15.72 |
| 12 | 113531407 | . | C/C | 0.001266 | 197 | 0 | 0 |  | 197 | 1 | 0 | 22.9 |
| 12 | 113531458 | . | G/A | 0.003788 | 197 | 0 | 0 |  | 196 | 3 | 0 | 28.6 |
| 12 | 113532663 | rs201809063 | G/A | 0.002558 | 195 | 1 | 0 |  | 194 | 1 | 0 | 11.47 |
| 12 | 113532684 | rs183848543 | C/T | 0.002538 | 197 | 0 | 0 |  | 195 | 2 | 0 | 14.39 |
| 12 | 113533205 | . | G/A | 0.001266 | 196 | 1 | 0 |  | 198 | 0 | 0 | 19.33 |
| *DTX1* | **1.86148** | **0.062784** |  |  |  |  |  |  |  |  |  |  |
|  |  |  |  |  |  |  |  |  |  |  |  |  |
| 22 | 41513755 | . | C/T | 0.001263 | 197 | 0 | 0 |  | 198 | 1 | 0 | 27 |
| 22 | 41527497 | . | C/A | 0.001263 | 196 | 1 | 0 |  | 199 | 0 | 0 | 32 |
| 22 | 41527628 | rs146242251 | A/G | 0.017767 | 193 | 4 | 0 |  | 187 | 10 | 0 | 15.46 |
| 22 | 41531828 | . | A/G | 0.001263 | 196 | 1 | 0 |  | 199 | 0 | 0 | 16.63 |
| 22 | 41533700 | . | A/G | 0.001263 | 197 | 0 | 0 |  | 198 | 1 | 0 | 18.45 |
| 22 | 41543855 | . | G/A | 0.001276 | 196 | 0 | 0 |  | 195 | 1 | 0 | 18.6 |
| 22 | 41543915 | . | C/A | 0.001263 | 197 | 0 | 0 |  | 198 | 1 | 0 | 15.51 |
| 22 | 41545774 | . | T/C | 0.002538 | 195 | 2 | 0 |  | 197 | 0 | 0 | 10.59 |
| 22 | 41545924 | . | C/T | 0.001263 | 197 | 0 | 0 |  | 198 | 1 | 0 | 28.2 |
| 22 | 41560079 | rs373249130 | G/A | 0.001263 | 197 | 0 | 0 |  | 198 | 1 | 0 | 26 |
| 22 | 41562607 | . | G/T | 0.001366 | 172 | 1 | 0 |  | 193 | 0 | 0 | 18.22 |
| 22 | 41569726 | . | A/G | 0.001263 | 196 | 1 | 0 |  | 199 | 0 | 0 | 14.45 |
| 22 | 41572288 | . | C/T | 0.001263 | 197 | 0 | 0 |  | 198 | 1 | 0 | 17.68 |
| 22 | 41573050 | . | T/G | 0.013405 | 196 | 0 | 0 |  | 167 | 10 | 0 | 15.75 |
| 22 | 41573269 | . | C/T | 0.001279 | 195 | 1 | 0 |  | 195 | 0 | 0 | 1.382 |
| 22 | 41573338 | rs200149159 | C/T | 0.002538 | 194 | 2 | 0 |  | 198 | 0 | 0 | 8.291 |
| 22 | 41574196 | rs188035979 | A/G | 0.012626 | 191 | 6 | 0 |  | 195 | 4 | 0 | 7.687 |
| 22 | 41574523 | . | G/C | 0.001269 | 195 | 1 | 0 |  | 198 | 0 | 0 | 14.88 |
| 22 | 41574628 | . | A/G | 0.001266 | 196 | 1 | 0 |  | 198 | 0 | 0 | 14.88 |
| 22 | 41574827 | . | C/G | 0.001263 | 197 | 0 | 0 |  | 198 | 1 | 0 | 53 |
| *EP300* | **2.32072** | **0.020328** |  |  |  |  |  |  |  |  |  |  |
|  |  |  |  |  |  |  |  |  |  |  |  |  |
| 1 | 32797709 | . | G/A | 0.001269 | 197 | 0 | 0 |  | 196 | 1 | 0 | 17.73 |
| *HDAC1* | **1** | **0.317927** |  |  |  |  |  |  |  |  |  |  |
|  |  |  |  |  |  |  |  |  |  |  |  |  |
| 20 | 10620282 | . | G/A | 0.001263 | 197 | 0 | 0 |  | 198 | 1 | 0 | 14.67 |
| 20 | 10620405 | rs373260040 | G/A | 0.001263 | 196 | 1 | 0 |  | 199 | 0 | 0 | 9.889 |
| 20 | 10620412 | . | C/T | 0.006313 | 194 | 3 | 0 |  | 197 | 2 | 0 | 13.96 |
| 20 | 10620418 | . | G/T | 0.001263 | 197 | 0 | 0 |  | 198 | 1 | 0 | 1.609 |
| 20 | 10621521 | . | C/T | 0.001266 | 195 | 1 | 0 |  | 199 | 0 | 0 | 13.31 |
| 20 | 10621771 | . | T/A | 0.001263 | 197 | 0 | 0 |  | 198 | 1 | 0 | 24.7 |
| 20 | 10621808 | rs200593413 | C/T | 0.001263 | 196 | 1 | 0 |  | 199 | 0 | 0 | 9.442 |
| 20 | 10622215 | . | G/A | 0.001269 | 196 | 0 | 0 |  | 197 | 1 | 0 | 14.33 |
| 20 | 10622501 | rs35761929 | G/C | 0.035443 | 184 | 12 | 1 |  | 184 | 14 | 0 | 20.9 |
| 20 | 10626016 | rs79176844 | T/G | 0.006812 | 196 | 0 | 0 |  | 166 | 5 | 0 | 23.6 |
| 20 | 10628673 | rs201785359 | G/A | 0.006313 | 196 | 1 | 0 |  | 195 | 4 | 0 | 35 |
| 20 | 10629255 | . | T/C | 0.002525 | 196 | 1 | 0 |  | 198 | 1 | 0 | 17.83 |
| 20 | 10630954 | . | A/C | 0.002525 | 197 | 0 | 0 |  | 197 | 2 | 0 | 10.53 |
| 20 | 10639284 | rs199674138 | C/T | 0.006313 | 195 | 2 | 0 |  | 196 | 3 | 0 | 13.2 |
| 20 | 10653554 | . | G/A | 0.003788 | 195 | 2 | 0 |  | 198 | 1 | 0 | 16.99 |
| 20 | 10653603 | rs183974372 | C/A | 0.003797 | 195 | 2 | 0 |  | 197 | 1 | 0 | 15.53 |
| 20 | 10654139 | . | G/T | 0.002688 | 111 | 0 | 0 |  | 74 | 1 | 0 | 15.48 |
| *JAG1* | **1.69903** | **0.089361** |  |  |  |  |  |  |  |  |  |  |
|  |  |  |  |  |  |  |  |  |  |  |  |  |
| 14 | 105609043 | rs201557978 | C/T | 0.001285 | 195 | 0 | 0 |  | 193 | 1 | 0 | 9.637 |
| 14 | 105609060 | . | T/C | 0.001282 | 194 | 1 | 0 |  | 195 | 0 | 0 | 5.101 |
| 14 | 105609234 | . | G/A | 0.001266 | 197 | 0 | 0 |  | 197 | 1 | 0 | 2.786 |
| 14 | 105609419 | . | C/A | 0.001279 | 196 | 0 | 0 |  | 194 | 1 | 0 | 18.39 |
| 14 | 105609836 | rs74913644 | G/A | 0.008929 | 194 | 3 | 0 |  | 191 | 4 | 0 | 0.01 |
| 14 | 105612247 | . | G/A | 0.001285 | 194 | 1 | 0 |  | 194 | 0 | 0 | 16.84 |
| 14 | 105613041 | . | A/C | 0.001272 | 195 | 1 | 0 |  | 197 | 0 | 0 | 14.9 |
| 14 | 105614696 | rs192887377 | C/T | 0.002577 | 195 | 1 | 0 |  | 191 | 1 | 0 | 12.47 |
| 14 | 105614734 | rs78154277 | C/T | 0.015306 | 189 | 6 | 0 |  | 191 | 6 | 0 | 14.32 |
| 14 | 105615518 | . | C/T | 0.001309 | 194 | 1 | 0 |  | 187 | 0 | 0 | 7.438 |
| *JAG2* | **-0.204111** | **0.838278** |  |  |  |  |  |  |  |  |  |  |
|  |  |  |  |  |  |  |  |  |  |  |  |  |
| 7 | 2552825 | rs187443334 | G/A | 0.008883 | 194 | 3 | 0 |  | 193 | 4 | 0 | 11.31 |
| 7 | 2564918 | . | G/A | 0.001289 | 194 | 0 | 0 |  | 193 | 1 | 0 | 32 |
| 7 | 2565079 | . | G/A | 0.001269 | 195 | 1 | 0 |  | 198 | 0 | 0 | 24.4 |
| 7 | 2565119 | . | C/G | 0.001279 | 194 | 1 | 0 |  | 196 | 0 | 0 | 12.94 |
| 7 | 2565976 | rs201791522 | G/A | 0.009021 | 193 | 3 | 0 |  | 188 | 4 | 0 | 9.39 |
| *LFNG* | **0.270145** | **0.787077** |  |  |  |  |  |  |  |  |  |  |
|  |  |  |  |  |  |  |  |  |  |  |  |  |
| 5 | 179159926 | . | C/T | 0.004274 | 70 | 0 | 0 |  | 46 | 1 | 0 | 3.034 |
| 5 | 179192490 | . | C/G | 0.001263 | 196 | 1 | 0 |  | 199 | 0 | 0 | 13.82 |
| 5 | 179193597 | . | G/C | 0.001272 | 196 | 1 | 0 |  | 196 | 0 | 0 | 5.456 |
| 5 | 179195867 | rs41285557 | G/A | 0.002525 | 196 | 1 | 0 |  | 198 | 1 | 0 | 7.998 |
| 5 | 179198228 | . | G/T | 0.001263 | 197 | 0 | 0 |  | 198 | 1 | 0 | 18.44 |
| *MAML1* | **0.106057** | **0.915549** |  |  |  |  |  |  |  |  |  |  |
|  |  |  |  |  |  |  |  |  |  |  |  |  |
| 11 | 95712465 | . | T/C | 0.001263 | 196 | 1 | 0 |  | 199 | 0 | 0 | 8.734 |
| 11 | 95712897 | rs7123133 | G/T | 0.034439 | 184 | 11 | 1 |  | 183 | 12 | 1 | 10.23 |
| 11 | 95718722 | . | T/C | 0.001282 | 191 | 1 | 0 |  | 198 | 0 | 0 | 16.37 |
| 11 | 95724685 | . | G/A | 0.001269 | 197 | 0 | 0 |  | 196 | 1 | 0 | 10.89 |
| 11 | 95825811 | . | A/C | 0.005063 | 195 | 2 | 0 |  | 196 | 2 | 0 | 8.866 |
| 11 | 95825852 | . | C/T | 0.001266 | 195 | 1 | 0 |  | 199 | 0 | 0 | 11.69 |
| 11 | 95825940 | . | C/T | 0.001263 | 196 | 1 | 0 |  | 199 | 0 | 0 | 4.441 |
| 11 | 95826417 | . | T/G | 0.002525 | 195 | 2 | 0 |  | 199 | 0 | 0 | 8.669 |
| 11 | 95826461 | . | G/A | 0.001263 | 196 | 1 | 0 |  | 199 | 0 | 0 | 8.533 |
| 11 | 95826575 | rs191391876 | C/T | 0.001263 | 196 | 1 | 0 |  | 199 | 0 | 0 | 9.956 |
| 11 | 96074675 | . | C/T | 0.005063 | 194 | 2 | 0 |  | 197 | 2 | 0 | 14.22 |
| 11 | 96074915 | . | G/T | 0.001266 | 195 | 1 | 0 |  | 199 | 0 | 0 | 15.85 |
| *MAML2* | **-1.06214** | **0.288228** |  |  |  |  |  |  |  |  |  |  |
|  |  |  |  |  |  |  |  |  |  |  |  |  |
| 22 | 37866063 | rs8192548 | G/A | 0.001269 | 195 | 1 | 0 |  | 198 | 0 | 0 | 11.5 |
| 22 | 37872964 | . | T/C | 0.001272 | 196 | 0 | 0 |  | 196 | 1 | 0 | 13.82 |
| 22 | 37875387 | . | C/T | 0.001263 | 197 | 0 | 0 |  | 198 | 1 | 0 | 14.89 |
| *MFNG* | **0.730416** | **0.465281** |  |  |  |  |  |  |  |  |  |  |
|  |  |  |  |  |  |  |  |  |  |  |  |  |
| 12 | 124809954 | . | G/A | 0.001502 | 168 | 1 | 0 |  | 164 | 0 | 0 | 3.04 |
| 12 | 124810004 | . | C/T | 0.00142 | 176 | 0 | 0 |  | 175 | 1 | 0 | 17.17 |
| 12 | 124810028 | rs77107801 | C/T | 0.014749 | 165 | 4 | 0 |  | 164 | 6 | 0 | 10.16 |
| 12 | 124810080 | . | A/C | 0.006557 | 171 | 1 | 0 |  | 130 | 3 | 0 | 8.256 |
| 12 | 124810741 | rs199646358 | G/A | 0.014031 | 192 | 4 | 0 |  | 189 | 7 | 0 | 2.49 |
| 12 | 124812069 | . | A/G | 0.002604 | 196 | 1 | 0 |  | 186 | 1 | 0 | 13.29 |
| 12 | 124812155 | . | G/A | 0.001266 | 196 | 1 | 0 |  | 198 | 0 | 0 | 19.75 |
| 12 | 124815439 | rs2228587 | T/C | 0.011539 | 190 | 4 | 0 |  | 191 | 5 | 0 | 9.015 |
| 12 | 124816920 | . | C/G | 0.001263 | 196 | 1 | 0 |  | 199 | 0 | 0 | 15.39 |
| 12 | 124817749 | . | T/C | 0.001266 | 196 | 1 | 0 |  | 198 | 0 | 0 | 2.637 |
| 12 | 124817756 | rs75362677 | C/T | 0.019133 | 187 | 8 | 0 |  | 190 | 7 | 0 | 4.445 |
| 12 | 124817789 | rs61751353 | A/G | 0.022727 | 188 | 9 | 0 |  | 190 | 9 | 0 | 0.066 |
| 12 | 124819006 | rs200545066 | G/A | 0.002849 | 178 | 0 | 0 |  | 171 | 2 | 0 | 14.19 |
| 12 | 124819043 | rs376063427 | T/C | 0.004545 | 164 | 2 | 0 |  | 163 | 1 | 0 | 5.162 |
| 12 | 124819100 | . | G/T | 0.001887 | 137 | 0 | 0 |  | 127 | 1 | 0 | 9.695 |
| 12 | 124819757 | rs373474834 | G/A | 0.001282 | 193 | 1 | 0 |  | 196 | 0 | 0 | 12.23 |
| 12 | 124821373 | . | G/A | 0.001269 | 195 | 1 | 0 |  | 198 | 0 | 0 | 12.34 |
| 12 | 124821385 | . | G/A | 0.001269 | 197 | 0 | 0 |  | 196 | 1 | 0 | 9.61 |
| 12 | 124821413 | rs2230944 | G/A | 0.017995 | 186 | 10 | 0 |  | 189 | 4 | 0 | 6.603 |
| 12 | 124821416 | . | C/T | 0.016753 | 186 | 9 | 0 |  | 189 | 4 | 0 | 12.06 |
| 12 | 124821495 | . | G/T | 0.001305 | 192 | 1 | 0 |  | 190 | 0 | 0 | 13.04 |
| 12 | 124821575 | rs369817436 | G/A | 0.001305 | 191 | 0 | 0 |  | 191 | 1 | 0 | 11.63 |
| 12 | 124824595 | . | C/A | 0.001272 | 196 | 0 | 0 |  | 196 | 1 | 0 | 13.33 |
| 12 | 124824955 | rs370212615 | C/T | 0.001263 | 197 | 0 | 0 |  | 198 | 1 | 0 | 0.071 |
| 12 | 124825196 | rs375289860 | C/T | 0.003927 | 190 | 2 | 0 |  | 189 | 1 | 0 | 9.487 |
| 12 | 124825227 | . | G/A | 0.002571 | 194 | 1 | 0 |  | 193 | 1 | 0 | 9.019 |
| 12 | 124829232 | . | G/A | 0.001282 | 195 | 1 | 0 |  | 194 | 0 | 0 | 13.58 |
| 12 | 124829278 | . | G/T | 0.001292 | 193 | 1 | 0 |  | 193 | 0 | 0 | 12.9 |
| 12 | 124829304 | . | G/A | 0.001279 | 195 | 1 | 0 |  | 195 | 0 | 0 | 10.03 |
| 12 | 124829376 | . | G/A | 0.001279 | 195 | 0 | 0 |  | 195 | 1 | 0 | 11.36 |
| 12 | 124835212 | rs76063811 | C/G | 0.001263 | 196 | 1 | 0 |  | 199 | 0 | 0 | 13.52 |
| 12 | 124838655 | rs184942554 | C/T | 0.003788 | 195 | 2 | 0 |  | 198 | 1 | 0 | 18.13 |
| 12 | 124839117 | . | C/T | 0.001316 | 195 | 0 | 0 |  | 184 | 1 | 0 | 14.31 |
| 12 | 124846833 | rs146881270 | T/G | 0.022161 | 178 | 13 | 0 |  | 168 | 1 | 1 | 12.33 |
| 12 | 124856847 | . | T/C | 0.001266 | 196 | 0 | 0 |  | 198 | 1 | 0 | 8.886 |
| 12 | 124862860 | rs371054468 | G/A | 0.001263 | 196 | 1 | 0 |  | 199 | 0 | 0 | 12.12 |
| 12 | 124904568 | . | T/A | 0.001263 | 196 | 1 | 0 |  | 199 | 0 | 0 | 16.63 |
| 12 | 124957636 | . | G/C | 0.002525 | 196 | 1 | 0 |  | 198 | 1 | 0 | 2.061 |
| 12 | 124968175 | . | C/T | 0.002558 | 196 | 0 | 0 |  | 194 | 0 | 1 | 0.101 |
| 12 | 124968240 | rs183845640 | T/C | 0.014139 | 190 | 5 | 0 |  | 188 | 6 | 0 | 12.2 |
| 12 | 124971071 | . | G/A | 0.001266 | 196 | 1 | 0 |  | 198 | 0 | 0 | 14.52 |
| *NCOR2* | **-2.04953** | **0.040427** |  |  |  |  |  |  |  |  |  |  |
|  |  |  |  |  |  |  |  |  |  |  |  |  |
| 1 | 160321499 | rs6664627 | C/T | 0.019084 | 190 | 7 | 0 |  | 188 | 8 | 0 | 9.326 |
| 1 | 160321887 | . | A/G | 0.001263 | 196 | 1 | 0 |  | 199 | 0 | 0 | 17.23 |
| 1 | 160321968 | rs200596888 | G/A | 0.001263 | 197 | 0 | 0 |  | 198 | 1 | 0 | 16.01 |
| 1 | 160322703 | . | G/C | 0.001263 | 197 | 0 | 0 |  | 198 | 1 | 0 | 6.018 |
| 1 | 160322763 | rs2296270 | A/G | 0.020253 | 191 | 6 | 0 |  | 188 | 10 | 0 | 8.536 |
| 1 | 160324027 | . | T/C | 0.001263 | 197 | 0 | 0 |  | 198 | 1 | 0 | 11.26 |
| 1 | 160326460 | rs115808642 | C/T | 0.001263 | 196 | 1 | 0 |  | 199 | 0 | 0 | 10.83 |
| *NCSTN* | **0.831** | **0.406045** |  |  |  |  |  |  |  |  |  |  |
|  |  |  |  |  |  |  |  |  |  |  |  |  |
| 14 | 73743807 | . | C/T | 0.001263 | 196 | 1 | 0 |  | 199 | 0 | 0 | 11.15 |
| 14 | 73746055 | . | G/A | 0.001263 | 196 | 1 | 0 |  | 199 | 0 | 0 | 34 |
| 14 | 73750944 | rs116089501 | T/C | 0.001263 | 197 | 0 | 0 |  | 198 | 1 | 0 | 24.1 |
| NUMB | **-0.496222** | **0.61983** |  |  |  |  |  |  |  |  |  |  |
|  |  |  |  |  |  |  |  |  |  |  |  |  |
| 9 | 139390567 | . | T/C | 0.001263 | 197 | 0 | 0 |  | 198 | 1 | 0 | 15.92 |
| 9 | 139390825 | . | T/C | 0.001323 | 189 | 0 | 0 |  | 188 | 1 | 0 | 1.554 |
| 9 | 139391152 | . | C/T | 0.001295 | 195 | 0 | 0 |  | 190 | 1 | 0 | 6.063 |
| 9 | 139391338 | rs61751489 | C/T | 0.002551 | 196 | 0 | 0 |  | 194 | 2 | 0 | 0.501 |
| 9 | 139391403 | rs200521815 | C/T | 0.006494 | 191 | 3 | 0 |  | 189 | 2 | 0 | 8.469 |
| 9 | 139391458 | rs201613894 | C/T | 0.001289 | 194 | 0 | 0 |  | 193 | 1 | 0 | 8.88 |
| 9 | 139391832 | . | C/T | 0.001269 | 195 | 1 | 0 |  | 198 | 0 | 0 | 19.15 |
| 9 | 139391840 | . | G/T | 0.001266 | 196 | 0 | 0 |  | 198 | 1 | 0 | 20.7 |
| 9 | 139396503 | . | C/T | 0.001263 | 197 | 0 | 0 |  | 198 | 1 | 0 | 24.6 |
| 9 | 139397646 | . | C/T | 0.001282 | 195 | 1 | 0 |  | 194 | 0 | 0 | 14.48 |
| 9 | 139399490 | . | G/T | 0.001272 | 195 | 0 | 0 |  | 197 | 1 | 0 | 14.27 |
| 9 | 139400302 | . | G/A | 0.001337 | 191 | 0 | 0 |  | 182 | 1 | 0 | 17.12 |
| 9 | 139401233 | rs61751543 | C/T | 0.001279 | 196 | 0 | 0 |  | 194 | 1 | 0 | 16.23 |
| 9 | 139401375 | . | C/T | 0.001279 | 194 | 0 | 0 |  | 196 | 1 | 0 | 9.4 |
| 9 | 139402484 | . | G/T | 0.001279 | 194 | 1 | 0 |  | 196 | 0 | 0 | 16.89 |
| 9 | 139402516 | rs374230681 | T/C | 0.01023 | 191 | 3 | 0 |  | 192 | 5 | 0 | 13.84 |
| 9 | 139404290 | . | C/T | 0.001263 | 196 | 1 | 0 |  | 199 | 0 | 0 | 22.7 |
| 9 | 139409035 | rs200692749 | T/G | 0.001337 | 196 | 0 | 0 |  | 177 | 1 | 0 | 16.12 |
| 9 | 139409041 | . | C/T | 0.001263 | 197 | 0 | 0 |  | 198 | 1 | 0 | 23 |
| 9 | 139409055 | . | G/C | 0.001266 | 197 | 0 | 0 |  | 197 | 1 | 0 | 14.89 |
| 9 | 139410063 | . | C/T | 0.001266 | 196 | 1 | 0 |  | 198 | 0 | 0 | 10.67 |
| 9 | 139410139 | rs369067940 | T/C | 0.001266 | 197 | 0 | 0 |  | 197 | 1 | 0 | 10.2 |
| 9 | 139413097 | rs200520088 | T/G | 0.022487 | 195 | 1 | 0 |  | 166 | 16 | 0 | 28.3 |
| 9 | 139413211 | rs202145498 | T/G | 0.021053 | 196 | 0 | 0 |  | 168 | 16 | 0 | 19.66 |
| NOTCH1 | **5.41964** | **6.12E-08** |  |  |  |  |  |  |  |  |  |  |
|  |  |  |  |  |  |  |  |  |  |  |  |  |
| 1 | 120458305 | . | C/T | 0.001266 | 196 | 0 | 0 |  | 198 | 1 | 0 | 13.3 |
| 1 | 120459050 | rs201584590 | T/C | 0.001263 | 197 | 0 | 0 |  | 198 | 1 | 0 | 4.983 |
| 1 | 120459215 | . | G/A | 0.001263 | 197 | 0 | 0 |  | 198 | 1 | 0 | 16.77 |
| 1 | 120462092 | rs148613210 | C/T | 0.001266 | 196 | 1 | 0 |  | 198 | 0 | 0 | 33 |
| 1 | 120462928 | . | C/A | 0.001263 | 197 | 0 | 0 |  | 198 | 1 | 0 | 12.78 |
| 1 | 120468156 | . | T/C | 0.001266 | 196 | 0 | 0 |  | 198 | 1 | 0 | 4.094 |
| 1 | 120469147 | rs61752484 | T/C | 0.001266 | 196 | 1 | 0 |  | 198 | 0 | 0 | 11.18 |
| 1 | 120471712 | rs75423398 | C/T | 0.010101 | 193 | 4 | 0 |  | 195 | 4 | 0 | 19.65 |
| 1 | 120484314 | rs201100122 | G/A | 0.002532 | 196 | 1 | 0 |  | 197 | 1 | 0 | 15.48 |
| 1 | 120491103 | . | T/G | 0.001263 | 197 | 0 | 0 |  | 198 | 1 | 0 | 11.12 |
| 1 | 120491109 | . | T/C | 0.008838 | 196 | 1 | 0 |  | 193 | 6 | 0 | 12.58 |
| 1 | 120491183 | . | C/T | 0.001263 | 196 | 1 | 0 |  | 199 | 0 | 0 | 8.175 |
| 1 | 120497840 | rs74882029 | A/T | 0.001263 | 196 | 1 | 0 |  | 199 | 0 | 0 | 13.58 |
| 1 | 120502047 | rs189684879 | C/T | 0.001263 | 197 | 0 | 0 |  | 198 | 1 | 0 | 10.67 |
| 1 | 120506352 | . | C/A | 0.007595 | 197 | 0 | 0 |  | 192 | 6 | 0 | 28.4 |
| 1 | 120539661 | rs146498360 | C/T | 0.003797 | 195 | 2 | 0 |  | 197 | 1 | 0 | 20.6 |
| NOTCH2 | **1.90156** | **0.057275** |  |  |  |  |  |  |  |  |  |  |
|  |  |  |  |  |  |  |  |  |  |  |  |  |
| 19 | 15271504 | . | T/G | 0.002538 | 194 | 2 | 0 |  | 198 | 0 | 0 | 9.207 |
| 19 | 15271523 | . | G/A | 0.001269 | 197 | 0 | 0 |  | 196 | 1 | 0 | 46 |
| 19 | 15272105 | rs372833545 | C/T | 0.001779 | 145 | 0 | 0 |  | 135 | 1 | 0 | 0.431 |
| 19 | 15272173 | . | G/A | 0.001618 | 166 | 0 | 0 |  | 142 | 1 | 0 | 14.27 |
| 19 | 15272409 | . | G/T | 0.001269 | 195 | 1 | 0 |  | 198 | 0 | 0 | 12.59 |
| 19 | 15276215 | . | C/T | 0.001263 | 196 | 1 | 0 |  | 199 | 0 | 0 | 13.64 |
| 19 | 15276695 | rs200883235 | C/T | 0.001276 | 195 | 0 | 0 |  | 196 | 1 | 0 | 35 |
| 19 | 15276717 | . | C/T | 0.001266 | 197 | 0 | 0 |  | 197 | 1 | 0 | 35 |
| 19 | 15276764 | rs202027632 | C/T | 0.002525 | 196 | 1 | 0 |  | 198 | 1 | 0 | 8.734 |
| 19 | 15281207 | . | G/T | 0.001272 | 196 | 1 | 0 |  | 196 | 0 | 0 | 11.15 |
| 19 | 15281580 | rs201167365 | T/A | 0.001289 | 195 | 1 | 0 |  | 192 | 0 | 0 | 17.8 |
| 19 | 15281582 | . | A/T | 0.002571 | 195 | 2 | 0 |  | 192 | 0 | 0 | 15.06 |
| 19 | 15285063 | rs141320511 | G/T | 0.00651 | 193 | 2 | 0 |  | 186 | 3 | 0 | 24.7 |
| 19 | 15289907 | . | G/A | 0.001266 | 197 | 0 | 0 |  | 197 | 1 | 0 | 12.34 |
| 19 | 15290031 | rs200504060 | G/A | 0.003788 | 196 | 1 | 0 |  | 197 | 2 | 0 | 16.86 |
| 19 | 15290911 | rs189545202 | C/T | 0.001269 | 195 | 1 | 0 |  | 198 | 0 | 0 | 14.15 |
| 19 | 15292460 | . | A/C | 0.001269 | 197 | 0 | 0 |  | 196 | 1 | 0 | 17.43 |
| 19 | 15295220 | . | C/T | 0.001266 | 195 | 1 | 0 |  | 199 | 0 | 0 | 9.654 |
| 19 | 15295807 | . | G/T | 0.001408 | 182 | 0 | 0 |  | 172 | 1 | 0 | 15.24 |
| 19 | 15296485 | . | G/A | 0.001263 | 197 | 0 | 0 |  | 198 | 1 | 0 | 11.92 |
| 19 | 15297721 | rs377689004 | C/T | 0.001266 | 197 | 0 | 0 |  | 197 | 1 | 0 | 23.6 |
| 19 | 15298083 | . | C/T | 0.001279 | 193 | 0 | 0 |  | 197 | 1 | 0 | 14.99 |
| 19 | 15302445 | . | C/A | 0.001269 | 196 | 1 | 0 |  | 197 | 0 | 0 | 17.57 |
| 19 | 15302649 | rs2285981 | C/T | 0.003797 | 197 | 0 | 0 |  | 195 | 3 | 0 | 15.22 |
| 19 | 15302951 | rs202157633 | G/A | 0.002545 | 195 | 1 | 0 |  | 196 | 1 | 0 | 8.139 |
| 19 | 15303297 | . | C/A | 0.001272 | 196 | 1 | 0 |  | 196 | 0 | 0 | 18.01 |
| NOTCH3 | **1.30713** | **0.191198** |  |  |  |  |  |  |  |  |  |  |
|  |  |  |  |  |  |  |  |  |  |  |  |  |
| 6 | 32163591 | . | C/G | 0.002688 | 184 | 1 | 0 |  | 186 | 1 | 0 | 16.54 |
| 6 | 32163799 | . | T/C | 0.03534 | 184 | 8 | 1 |  | 173 | 15 | 1 | 11.85 |
| 6 | 32168733 | . | G/A | 0.001263 | 197 | 0 | 0 |  | 198 | 1 | 0 | 15.13 |
| 6 | 32168969 | . | C/T | 0.001263 | 196 | 1 | 0 |  | 199 | 0 | 0 | 9.514 |
| 6 | 32168996 | . | C/G | 0.002525 | 197 | 0 | 0 |  | 197 | 2 | 0 | 12.75 |
| 6 | 32169083 | . | C/T | 0.001269 | 196 | 0 | 0 |  | 197 | 1 | 0 | 15.8 |
| 6 | 32170102 | . | C/T | 0.001269 | 197 | 0 | 0 |  | 196 | 1 | 0 | 13.15 |
| 6 | 32178583 | rs189882422 | C/A | 0.001269 | 197 | 0 | 0 |  | 196 | 1 | 0 | 7.981 |
| 6 | 32180930 | . | C/T | 0.001263 | 196 | 1 | 0 |  | 199 | 0 | 0 | 10.76 |
| 6 | 32181473 | . | C/T | 0.001263 | 196 | 1 | 0 |  | 199 | 0 | 0 | 14.22 |
| 6 | 32184830 | rs150609093 | G/A | 0.001263 | 196 | 1 | 0 |  | 199 | 0 | 0 | 9.962 |
| 6 | 32184835 | rs151131761 | C/T | 0.008838 | 195 | 2 | 0 |  | 194 | 5 | 0 | 16.65 |
| 6 | 32185796 | rs8192591 | C/T | 0.016539 | 190 | 6 | 0 |  | 190 | 7 | 0 | 13.38 |
| 6 | 32185828 | . | A/G | 0.001266 | 197 | 0 | 0 |  | 197 | 1 | 0 | 11.92 |
| 6 | 32187906 | rs200731043 | C/G | 0.006329 | 192 | 5 | 0 |  | 198 | 0 | 0 | 18.59 |
| 6 | 32188823 | rs386616293 | G/A | 0.020305 | 189 | 6 | 0 |  | 189 | 10 | 0 | 0.03 |
| 6 | 32188853 | rs192478549 | C/T | 0.008861 | 193 | 3 | 0 |  | 195 | 4 | 0 | 7.468 |
| 6 | 32188872 | . | C/T | 0.001263 | 196 | 1 | 0 |  | 199 | 0 | 0 | 14.4 |
| 6 | 32188943 | rs386556332 | G/A | 0.032051 | 183 | 11 | 0 |  | 182 | 14 | 0 | 10.54 |
| NOTCH4 | **0.971678** | **0.331242** |  |  |  |  |  |  |  |  |  |  |
|  |  |  |  |  |  |  |  |  |  |  |  |  |
| 14 | 73637584 | . | A/G | 0.001263 | 196 | 1 | 0 |  | 199 | 0 | 0 | 11.18 |
| 14 | 73673178 | . | A/G | 0.001323 | 180 | 1 | 0 |  | 197 | 0 | 0 | 11.07 |
| 14 | 73678523 | rs116640707 | C/T | 0.001266 | 197 | 0 | 0 |  | 197 | 1 | 0 | 18.15 |
| PSEN1 | **-0.198128** | **0.84298** |  |  |  |  |  |  |  |  |  |  |
|  |  |  |  |  |  |  |  |  |  |  |  |  |
| 1 | 227069673 | . | C/T | 0.001266 | 196 | 0 | 0 |  | 198 | 1 | 0 | 15.91 |
| 1 | 227069708 | rs200636353 | G/A | 0.005102 | 197 | 0 | 0 |  | 191 | 4 | 0 | 28.6 |
| 1 | 227071448 | rs150400387 | C/T | 0.001263 | 197 | 0 | 0 |  | 198 | 1 | 0 | 17.23 |
| 1 | 227075798 | . | C/A | 0.001269 | 195 | 0 | 0 |  | 198 | 1 | 0 | 23 |
| 1 | 227076603 | . | G/T | 0.005051 | 195 | 2 | 0 |  | 197 | 2 | 0 | 26.4 |
| 1 | 227076640 | . | T/C | 0.001266 | 196 | 0 | 0 |  | 198 | 1 | 0 | 11.7 |
| PSEN2 | **2.12571** | **0.033631** |  |  |  |  |  |  |  |  |  |  |
|  |  |  |  |  |  |  |  |  |  |  |  |  |
| 19 | 36237692 | . | C/T | 0.001266 | 197 | 0 | 0 |  | 197 | 1 | 0 | 18.89 |
| PSENEN | **0.997465** | **0.319153** |  |  |  |  |  |  |  |  |  |  |
|  |  |  |  |  |  |  |  |  |  |  |  |  |
| 20 | 43938244 | . | G/A | 0.002525 | 196 | 1 | 0 |  | 198 | 1 | 0 | 8.467 |
| 20 | 43942183 | . | C/G | 0.001263 | 196 | 1 | 0 |  | 199 | 0 | 0 | 17.35 |
| 20 | 43945527 | . | C/G | 0.001263 | 196 | 1 | 0 |  | 199 | 0 | 0 | 13.55 |
| RBPJL | **-1.24437** | **0.21361** |  |  |  |  |  |  |  |  |  |  |
|  |  |  |  |  |  |  |  |  |  |  |  |  |
| 17 | 80008394 | rs202206613 | C/T | 0.001269 | 194 | 1 | 0 |  | 199 | 0 | 0 | 8.113 |
| 17 | 80008542 | . | G/A | 0.001263 | 197 | 0 | 0 |  | 198 | 1 | 0 | 13.71 |
| RFNG | **0.34094** | **0.73324** |  |  |  |  |  |  |  |  |  |  |
|  |  |  |  |  |  |  |  |  |  |  |  |  |
| 14 | 78217669 | . | T/C | 0.001266 | 197 | 0 | 0 |  | 197 | 1 | 0 | 29.7 |
| 14 | 78221339 | . | G/A | 0.001266 | 196 | 1 | 0 |  | 198 | 0 | 0 | 18.96 |
| SNW1 | **0.301307** | **0.76326** |  |  |  |  |  |  |  |  |  |  |
