# Supplementary material for: Targeted sequencing of NOTCH signaling pathway genes and association analysis of variants correlated with mandibular prognathism
Source: Head Face Med. 2021 May 26;17:17. doi: 10.1186/s13005-021-00268-0 (PMC8152080; doi:10.1186/s13005-021-00268-0)
Supplement: Supplementary file 7 — Additional file 7: Figure S1. Three SNPs (rs1051415 in an exonic region of JAG1; rs75236173 in the 3′-UTR of NUMB; rs1044006 in an exonic region of NOTCH3) in six randomly selecting samples (including three cases and three controls) were validated by Sanger sequencing. The three cases were C02, C03 and C8, while the three control samples were A8, A42 and A43. The black arrows represent the polymorphism sites. [file 13005_2021_268_MOESM7_ESM.pdf]

Fig S1. The detailed diagrams for the repeated measures of targeted region sequencing results.

①rs1051415

A8

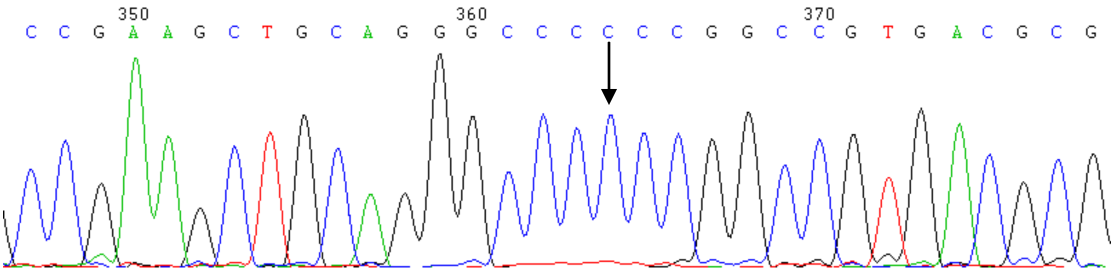

A42

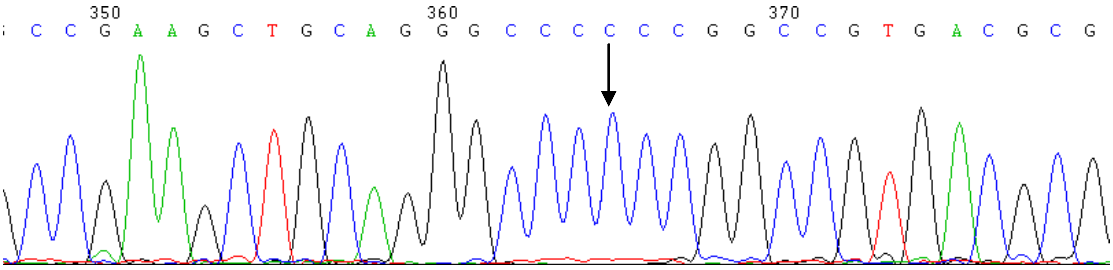

A43

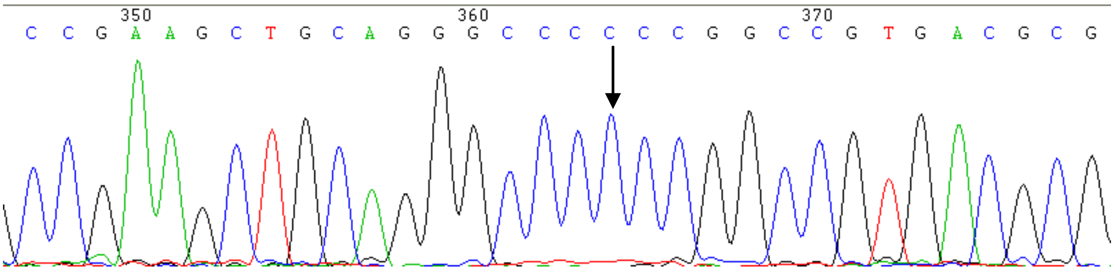

C02

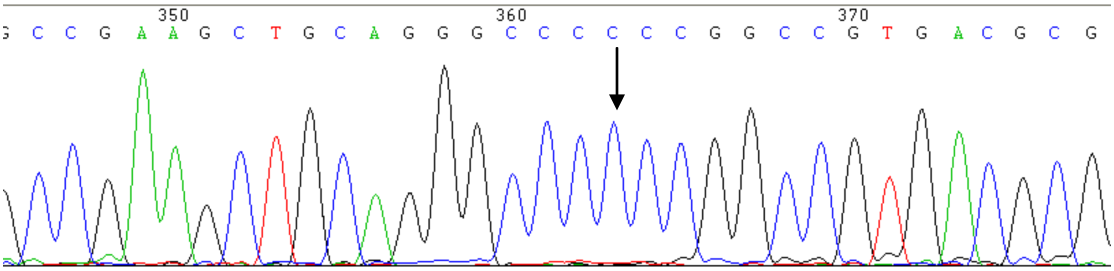

C03

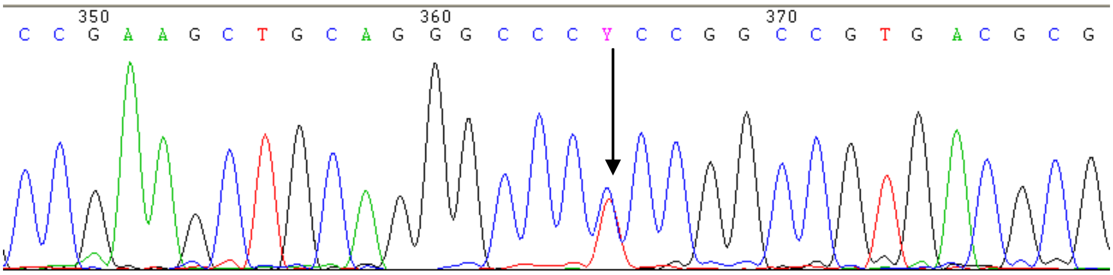

C8

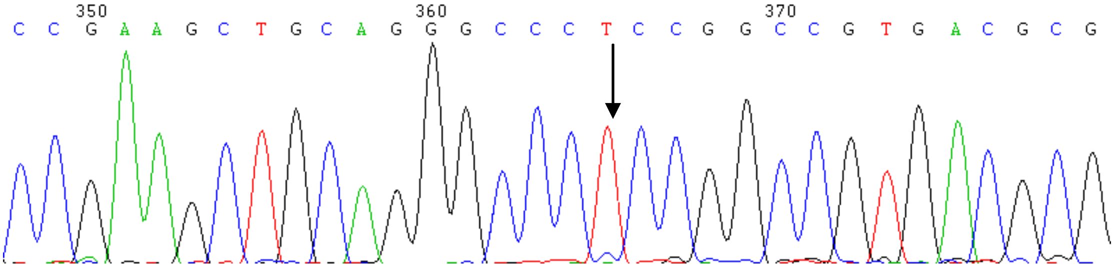

②rs75236173.

A8

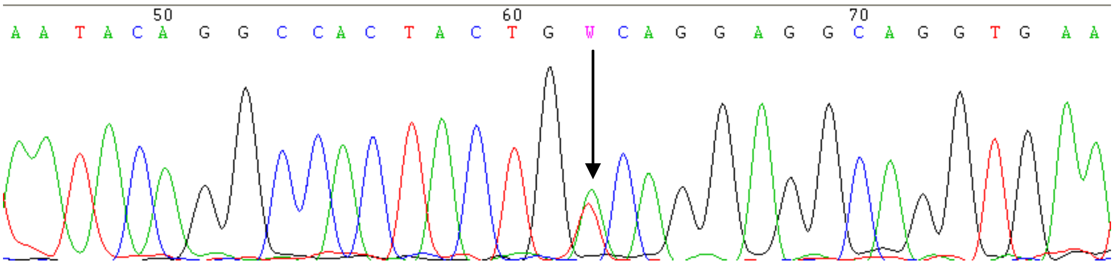

A42

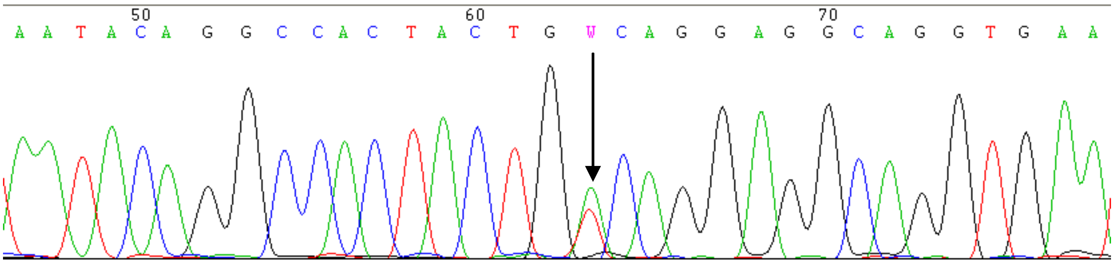

A43

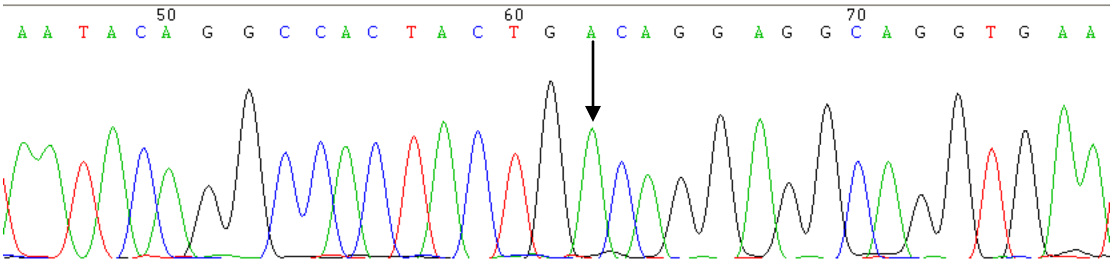

C02

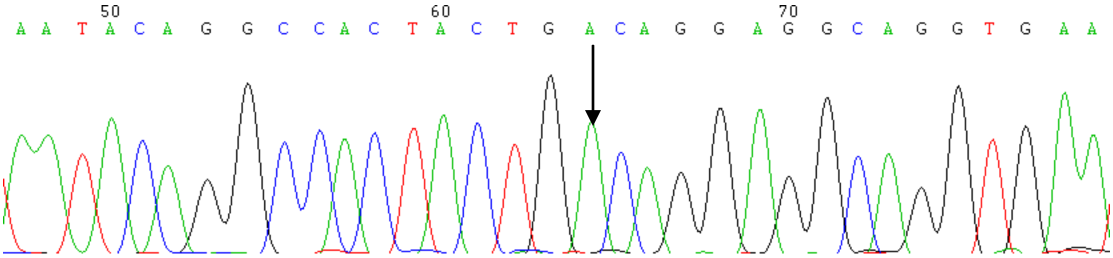

C03

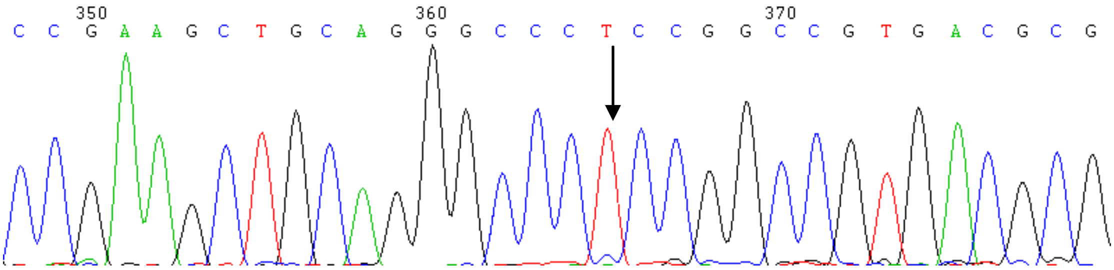

C8

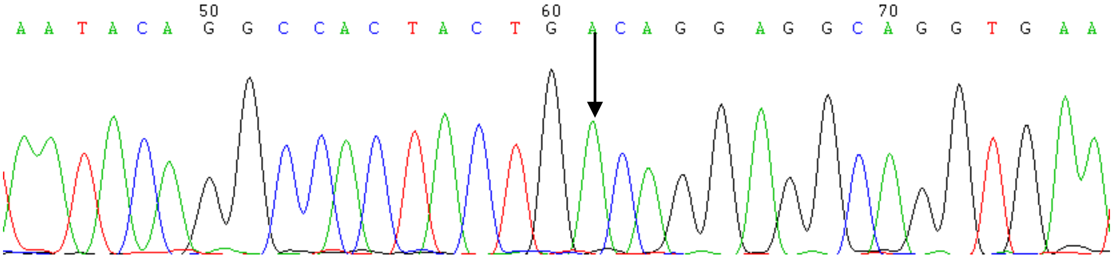

③rs1044006

A8

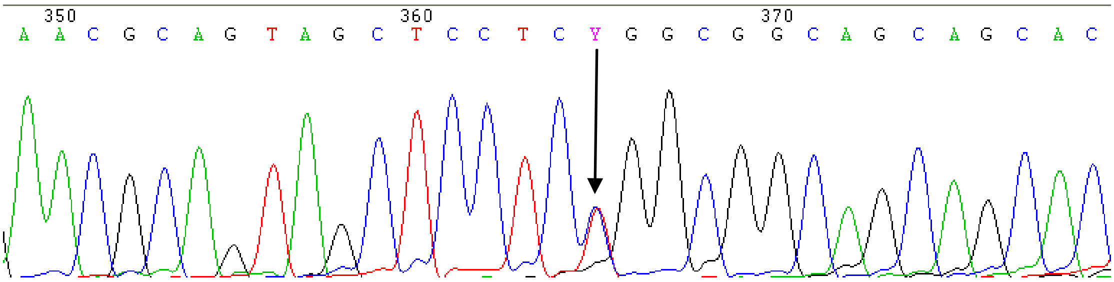

A42

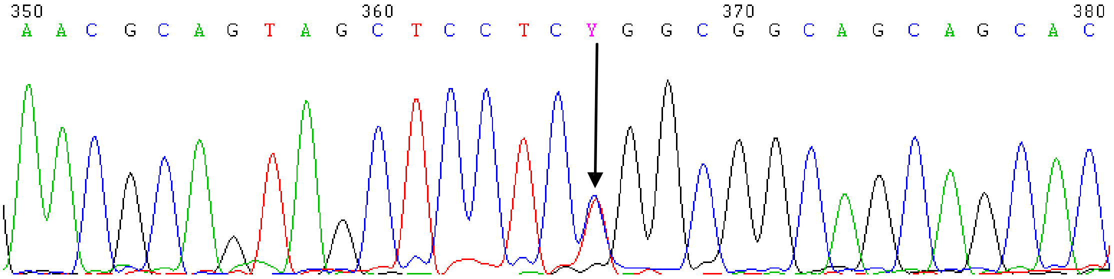

A43

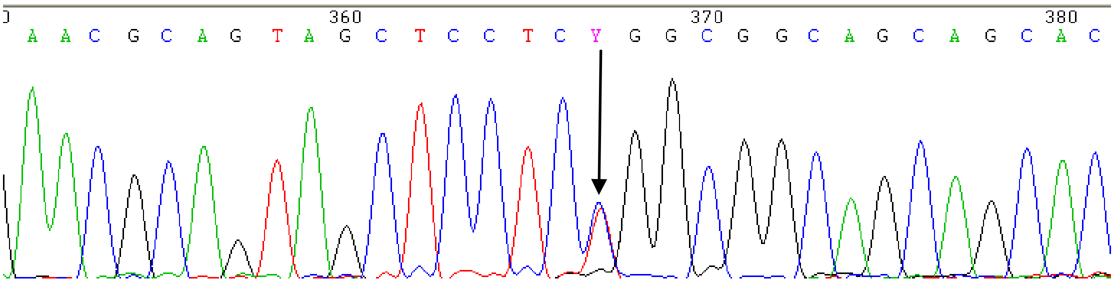

C02

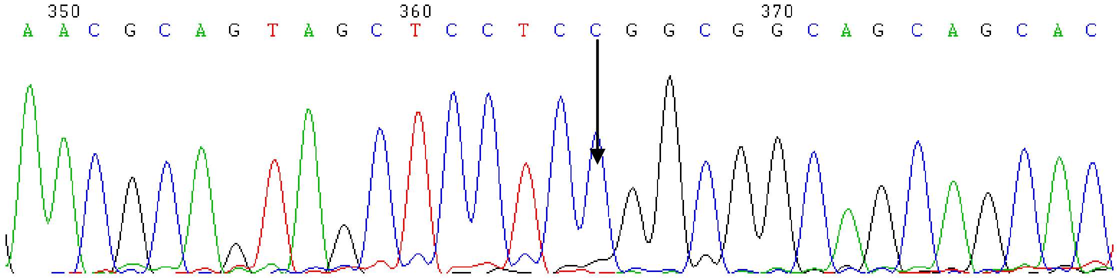
350 360 370
A A C G C A G T A G C T C C T C C G G C G G C A G C A G C A C

C03

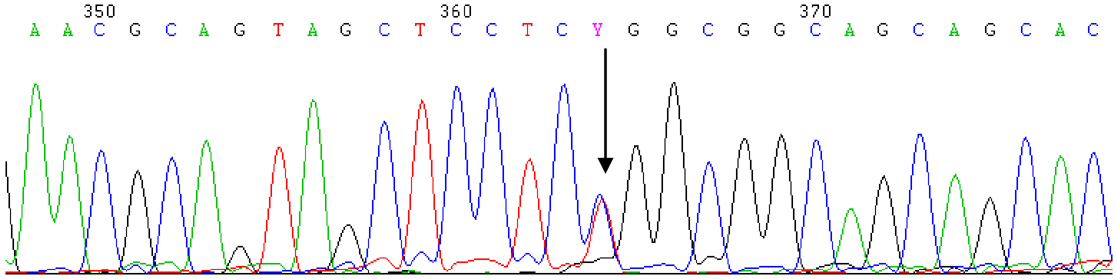
350 360 370
A A C G C A G T A G C T C C T C Y G G C G G C A G C A G C A C

C8

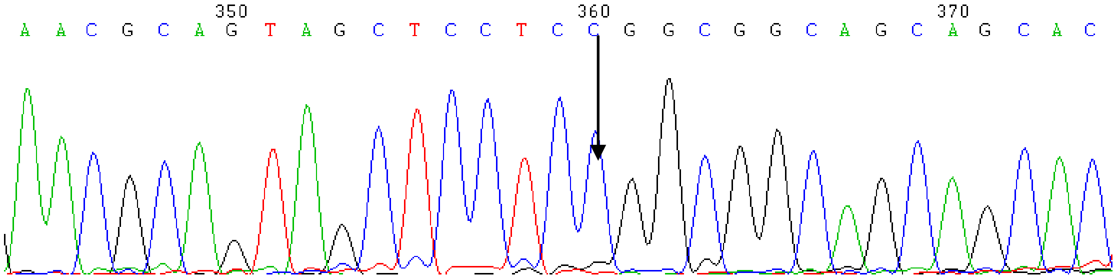
350 360 370
A A C G C A G T A G C T C C T C C G G C G G C A G C A G C A C
